# Investigating the impact of long-term bristlegrass coverage on rhizosphere microbiota, soil metabolites, and carbon–nitrogen dynamics for pear agronomic traits in orchards

**DOI:** 10.3389/fmicb.2024.1461254

**Published:** 2024-09-05

**Authors:** Chunhui Shi, Xiaoqing Wang, Shuang Jiang, Jianfeng Xu, Jun Luo

**Affiliations:** ^1^Forest & Fruit Tree Research Institute, Shanghai Academy of Agriculture Sciences, Shanghai, China; ^2^College of Horticulture, Hebei Agricultural University, Baoding, Hebei, China; ^3^Shanghai Key Laboratory of Facility Horticulture Technology, Shanghai Academy of Agriculture Sciences, Shanghai, China

**Keywords:** grass coverage, carbon sequestration, mineral nutrient, rhizospheric microorganism, PGPRs, metabolomics, yield

## Abstract

**Background:**

Grass coverage (GC) under no-tillage systems in orchards signifcantly infuences underground carbon (C) and nitrogen (N) sequestration, primarily through promoting mineral nutrient utilization by rhizospheric microorganisms. However, the comprehensive impact of GC on microbial communities and plant responses using soil metabolomics remains inadequately recognized.

**Methods:**

We investigated two rhizosphere types established since 2002: bristlegrass (*Setaria viridis* (L.) P. Beauv.) coverage (SC) and clean cultivation (CC) to assess their efects on soil parameters, enzyme activities, and key pear agronomic traits, including yield (single fruit weight (SFW)) and qualities (soluble solids content (SSC), and total soluble sugar (TSS)). We combined microbiological analysis (16S rRNA sequencing) and non-targeted metabolomics (UPLC-MS/MS and GC–MS) to explore how microbial communities infuence fruit agronomic traits and soil nutrient dynamics in pear orchards under SC conditions.

**Results:**

Our fndings indicate that SC signifcantly enhances soil organic carbon (SOC), soil organic nitrogen (SON), the C:N ratio, and available nitrogen (AN). Moreover, SC leads to pronounced increases in soil enzyme activities involved in the C cycle and storage, including soil sucrase, β-glucosidase, polyphenol oxidase and cellulase. Microbiome analysis revealed substantial diferences in microbial community composition and diversity indices between SC and CC rhizosphere soils within the 0–40 cm depth. Metabolomic analysis demonstrated significant alterations in metabolite profiles across both the 0-20 cm and 20-40 cm layers under SC conditions. The identifed metabolites primarily involve sugar and amino acid-related metabolic pathways, refecting perturbations in C and N metabolism consistent with shifts in bacterial community structure. Several plant growth-promoting rhizobacteria (PGPRs) taxa (e.g., *Haliangium*, *Bacteroides*, *mle1-7*, *Subgroup_22*, *Ellin6067*, *MND1*, *Flavobacterium*, and *Cellvibrio*) were enriched under SC, associated with metabolites such as sucrose, N-acetyl-D-glucosamine, N-acetyl-L-glutamic acid, rhamnose, UDP-GlcNAc and D-maltose. These fndings suggest their roles in promoting C and N sequestration processes through sucrose synthesis and glycolytic pathways in the soil, which was signifcantly correlated with the formation of agronomic traits such as fruit yield, SFW SSC and TSS (p<0.05), and SC treatments signifcantly increased yields by 35.40–62.72% and sucrose content in TSS by 2.43–3.96 times than CC treatments.

**Conclusion:**

This study provides valuable insights into the efects of SC on soil microbial communities and plant physiology, enhancing our understanding of their implications for sustainable orchard management.

## 1 Introduction

Grasslands are pivotal ecological systems globally. However, the persistent conversion of grasslands into arable land has resulted in significant degradation of terrestrial ecosystems (Baer et al., 2015; Song et al., 2022). Additionally, to satisfy the increasing fruit demand from expanding populations, orchard expansion has adversely impacted ecosystems worldwide (Bardgett et al., 2021). Clean cultivated (CC) orchards, characterized by clean tillage practices, substantially contribute to soil erosion and degradation (Mikha et al., 2018; Chen et al., 2021). Unfortunately, CC orchards remain prevalent across numerous regions, including North America, the Middle East, and other areas. The substantial global standing of China’s pear industry has drawn researchers’ attention to the issue of soil health within pear orchards. According to the Food and Agriculture Organization’s 2021 statistics, Chinese pear orchards encompass an extensive area of 921,610 hectares, accounting for two-thirds of the planting area and 70% of the total global yield. Notably, CC pear orchards represent over 80% of orchard areas in China, significantly contributing to soil erosion and degradation in these environments (Xiang et al., 2023).

The conversion of grasslands to arable land often results in a reduction in soil organic carbon (SOC) stocks (Oberholzer et al., 2014; Li et al., 2016). Conversely, decreasing tillage intensity and implementing grass cover can mitigate the decline of soil organic matter (SOM) and soil biota, thereby maintaining soil structure and crop yield (Sleiderink et al., 2024). The depletion of SOC in orchards is more pronounced compared to that in cereal production, attributable to suboptimal management practices (Seitz et al., 2023). Long-term clean cultivation has been linked to a rapid loss of SOC (Xiang et al., 2023). Sugiura et al. (2017) demonstrated that even with the application of chemical fertilizers, CC orchards still experienced significant carbon loss compared to orchards with mulching grass. Furthermore, CC practices result in greater runoff losses of available nitrogen (AN) and available phosphorus (AP) compared to conditions where grass is allowed to grow and mulch naturally (Wang et al., 2016).

Grasslands play a crucial role in carbon sequestration, and the reintroduction of grasses has been widely adopted as an effective soil management practice in pear orchards across Europe, America, Japan, and other countries (Wang et al., 2017; Peters et al., 2019; Yang et al., 2022). Numerous studies have confirmed that grasses contribute to the restoration of soil health by enhancing nutrient cycling within the soil. This is particularly evident when perennial cover plants are grown between rows or in orchards without soil tillage (Qian et al., 2015). Converting CC orchards to grass-covered ones alters the abundance and composition of soil microbial communities in various orchards, including those for pears, apples, and jujubes (Li et al., 2022; Wang et al., 2020). Extensive research has examined changes in microbial biomass and diversity following the enhancement of soil carbon stocks with different grass species (Qian et al., 2015; Li et al., 2022; Xiang et al., 2023). However, the microbial mechanisms underlying soil organic mineralization and fruit tree response mechanisms remain unclear. It has been shown that restoring plant growth-promoting microorganisms (PGPMs) in orchards through the use of locally dominant natural grasses is vital for sustainable agriculture (Debasis et al., 2019). Several studies have demonstrated that grass coverage serves as a protective layer, accelerating soil nutrient cycling and facilitating SOC sequestration by enhancing microbial activity, ultimately improving plant and fruit growth (Fang et al., 2021; Gómez et al., 2022).

In recent years, 16S rRNA gene sequencing has been utilized to quickly and accurately identify the diversity of bacterial communities, including previously unknown bacterial species (Zakrzewski et al., 2012). This technique has been employed to analyze the carbon source utilization characteristics of microbial communities following intercropping, resulting in significant increases in microbial diversity indices and enhanced utilization of carboxylic acids, phenolic acids, amines, carbohydrates, amino acids, and other carbon substrates (Peng et al., 2017). Soil enzymes also play pivotal roles in the soil carbon cycle and serve as indicators of soil health. Soil microorganisms facilitate organic matter decomposition through the secretion of diverse enzymes, thereby influencing mineralization processes and impacting plant growth either positively or negatively (Fan et al., 2019). Soil cellulases (S-CL), hydrolytic enzymes responsible for breaking down simple substrates, and soil polyphenol oxidase (S-PPO), which decomposes recalcitrant substrates, are integral components of this cycle. Differences in soil enzyme activities between clean and sod cultivation modes were observed, with the highest invertase activity (including alkaline phosphatase, invertase, and catalase) in the 0–40 cm soil layer of orchards (Xiang et al., 2023). Researchers found that covering orchards with different grass seeds affected the proportion of soil fungi and bacteria (Wu et al., 2010; Wang et al., 2020). In a walnut orchard with grass cover, notable outcomes were observed compared to clean cultivation tillage. Soil samples under grass cover exhibited a significant enrichment of PGPRs, including notable genera such as Pseudomonas, Paraburkholderia, Burkholderia, Agrobacterium, and Flavobacterium (Wang et al., 2023). Specific PGPR genera were identified in apple orchard soils under varying long-term grass cover patterns. In addition, Clostridium, known for promoting soil nitrogen accumulation, was prevalent in the grass-covered model (Qian et al., 2015; Zhang et al., 2023).

Natural grass confers several adaptive advantages over artificial grasslands, enhancing soil physical properties in pear orchard cultivation layers and increasing microbial biomass C and N (Wang et al., 2016). However, the relationship between increased C and N and the emergence of new bacterial species in cultivated soil warrants further investigation. Green bristlegrass (*Setaria viridis* (L.) P. Beauv.) coverage (SC) is widely distributed across temperate and subtropical regions in Asia, Europe, America, and Australia. It is one of the most advantageous grass varieties. Due to its strong adaptability and favorable growing season aligned with soil moisture conservation for orchard grasses, it is the preferred natural grass variety. During the dry season of pear growth, there was an increase in grass varieties and yield, with dry grass yielding 6,360 kg·ha^−1^. This yield advantage compared to artificial turf also results in significant advantages in soil nutrient nitrogen, phosphorus, and potassium for regressing orchard areas (Liu et al., 2017). In addition, the average growth rate of SOC and total nitrogen (TN) in natural SC mode was higher than that in artificial grass growing mode in the orchard. SOC and TN had positive effect load and weight on yield and quality of fruit agronomic traits, especially soluble solid content (SSC) in fruit (Aghajani et al., 2008; Ku et al., 2018). Two genotypes of green bristlegrass (*Salvia*) accessions on poor soil were found to alter the abundance of the soil microbial community, particularly the group of deformable bacteria, compared to the bare soil, the rhizosphere soil of *Setaria italica* exhibited significantly higher values of organic carbon and total nitrogen, and the nitrogen-containing metabolites tyrosine-derived alkaloids, serotonin, and synephrine were also significantly increased in the rhizosphere (Peterson et al., 2021). Yet, studies on dominant PGPMs in natural grass and their correlations with pear fruit agronomic traits remain limited. Specifically, deeper exploration into the biological mechanisms of plant–microbe interactions, soil–microbe interactions, and their metabolic cycles is crucial. We speculate that intercropping pear trees with green grass and other crops may disrupt the rhizosphere microbial population and secretions of pear trees, thereby altering the enzyme activity released into the soil by microorganisms and further regulating C and N sources. Further experiments are needed to verify this hypothesis. We employed MiSeq sequencing of 16S rRNA genes alongside non-targeted metabolomics (ultra-performance liquid chromatography–tandem mass spectrometry (UPLC-MS/MS) and gas chromatography–mass spectrometry (GC–MS/MS)) to analyze the impact of SC versus CC in two soil layers (0–20 cm and 20–40 cm). Our study aimed to (1) evaluate the influence of natural green bristlegrass on carbon sequestration in degraded orchard soil; (2) investigate how rhizosphere soil microbial communities and secreted metabolites influence the C and N cycles and the biochar restoration mechanism in SC; and (3) explore the network between PGPMs and pear fruit development and quality through C and N cycles. Capturing beneficial PGPMs from SC and reintroducing them into pear orchards prove effective in sequestering biological C and N, offering a theoretical basis for developing specialized microbial fertilizers for pears as an alternative to excessive mineral fertilization.

## 2 Materials and methods

### 2.1 Site description

**Figure 1:**
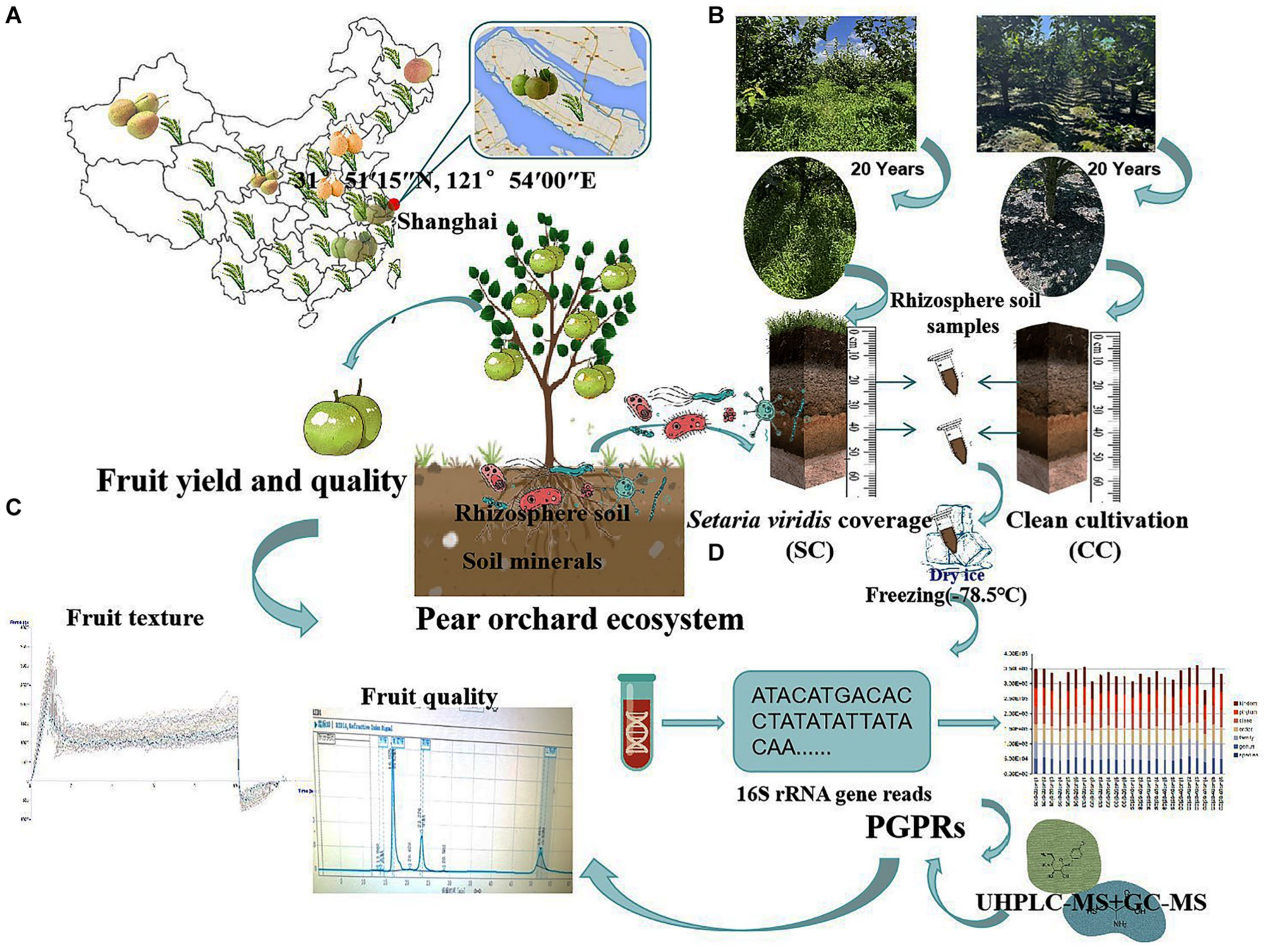
Sampling flow chart, evaluation of the agronomic traits contribution of pear, the soil nutrition, and the vertical distribution patterns of rhizosphere soil bacteria communities between SC mode and CC mode in different rhizosphere soil layers. **(A)** The geographic locations of the sampling site. **(B)** The schematic for collecting and preparing vertical rhizosphere soil samples in a pear orchards. **(C)** The schematic for measuring fruit yield and quality of aboveground pear trees in pear orchard. **(D)** The schematic for high-throughput sequencing and metabolite analysis of bacteria community in SC and CC rhizosphere soil at ASV level.

This study was conducted on the Chongming island of Shanghai, in the eastern region of China (31°51′15″N, 121°54′00″E), south of the Yangtze River, in an area suitable for the growth of sand pear (*Pyrus pyrifolia* (Burm. f.) Nakai) (Figure 1A). The orchard soil was originally saline-alkaline (pH = 7.70) and primarily sandy loam, which is ideal for tillage. The primary root system of the pear trees was concentrated at a depth of approximately 40 cm.

### 2.2 Experimental design and sampling

‘Cuiguan’ pear (*Pyrus pyrifolia* (Burm. f.)) trees, 20 years old and planted at a density of 1,005 plants/ha, were established in mid-February 2003. Two treatment groups were established: clean cultivated rhizosphere soil (CC) as a control and green bristlegrass (*Setaria viridis* (L.) P. Beauv.) coverage (SC) of rhizosphere soil for over 20 years. A random-arrangement design was adopted, with 20 trees per experimental group. Traditional management approaches were used for cultivation and pest control.

Rhizosphere soil samples were collected during the most active growth period of pear tree roots (October 12) using a soil sampler from two vertical soil layers (0–20 cm and 20–40 cm) within a 10 cm radius around each pear tree. Three replicates were selected for each sample using the ‘S’ shape sampling method (Zeng et al., 2021). After removing the rubble and soil impurities, the remaining soil was stored in sterile plastic containers. The collected soil samples were divided into two parts: One part was kept at −80°C for microbial community and non-target metabolomics analysis, while the other part was stored at −20°C for soil physicochemical parameter and enzyme activity evaluations (Figure 1B).

### 2.3 Soil physicochemical parameters and enzyme activity analysis

Electrical conductivity (EC values), potential of hydrogen (pH), and extractable medium/microelement contents (Ca, Mg, Cu, Fe, Mn, Zn, and B) were determined according to the protocol of Shi et al. (2023). The rhizosphere soil samples were analyzed for total carbon (TC), total organic carbon (SOC), total nitrogen (TN), total organic nitrogen (SON), and carbon-to-nitrogen ratio (C:N) using an automatic elemental analyzer (Primacs™ SNC-100; Skalar, China) (Dobosy et al., 2016). AN, AP, and available potassium (AK) in the pear root-zone soil under SC and CC treatments were determined according to the procedure outlined by Ma et al. (2021, 2022). Soil microbial biomass carbon (MBC) and soil microbial biomass nitrogen (MBN) were determined using the chloroform fumigation K_2_SO_4_ extraction method (Kumar et al., 2021). Soil sucrase (S-SC), β-glucosidase (S-β-GC), soil polyphenol oxidase (S-PPO), soil peroxidase (S-POD), and soil cellulase (S-CL) were determined using the visible spectrophotometric method, and kits were acquired from Shanghai Enzyme Linked Biotechnology Co., Ltd.

### 2.4 Agronomic contribution to pear fruit yield, quality, and nutritive value

The ‘Cuiguan’ pears reached maturity on 28 July from 2022 to 2023. For each treatment, a single plant was considered as an experimental unit for statistical evaluation, with three replicates for each treatment. Twenty fruits from each of the five pear trees were randomly selected from the upper, middle, and lower parts of the canopy. Single fruit weight (SFW) was weighed using an electronic balance (ME-T; Thermo Fisher, USA). Fruit number per plant (FNP) was determined using a counter. The lateral diameter (LD) and vertical diameter (VD) of the fruits were measured using an electronic caliper (505–732, Mitutoyo, Japan). During the harvest period, the middle portion of the soluble solids content (SSC) was measured using a handheld refractometer (Master-20a; ATAGO, Japan). To determine the total soluble sugar (TSS) content of pear fruits, a protocol previously described by Shi et al. (2023) was used with minor modifications. Fresh pear juice was extracted from the top, middle, and bottom of 20 randomly selected fruits from each treatment group using a juicer (Avance Collection HR1922/20; Philips, Netherlands). Subsequently, 2 mL of raw juice was diluted four times. Sucrose, glucose, fructose, and sorbitol contents were calculated using the external standard method, and the sum of these four sugars was considered as the TSS content. For further analysis, 1 mL of the supernatant was used for sugar and acid composition analyses by high-performance liquid chromatography (Waters e2695, USA). SS content was determined using a Ca-type sugar column (CARBOSep CHO-620; Transgenomic, USA) detected by a Water 2,414 Refractive Index Detector at 90°C, with a flow rate of 0.5 mL.min-1. The organic acid (OA) composition and content were determined using a C18 column (CNW Athena C18-WP; CNW, Germany) detected by Water 2,998 Photodiode Array Detector, with 0.2% metaphosphate as the mobile phase at a flow rate of 0.5 mL.min-1 and a temperature of 37°C. The sugar and acid standards used were of chromatographic purity provided by Sigma Corporation (USA). TSS and OA were mixed with different concentration gradients, and standard curves were drawn to measure their respective levels. The OA in the pears mainly consisted of tartaric acid, quinic acid, malic acid, and citric acid. Fruit texture was determined using a texture analyzer (TA-XT plus C; Stable Micro Systems, UK). Three test points were evenly spaced at the equatorial part of the fruit. A test probe P/5, cylindrical with a 5 mm diameter, was used with a test speed of 1 mm/s, test depth of 8 mm, and trigger value of 0.05 N (Figure 1C).

### 2.5 Soil DNA extraction and high-throughput sequencing of rhizosphere soil microbial communities

Pear tree rhizosphere soil samples from the SC and CC groups for two vertical soil layers (0–20 cm and 20–40 cm) were collected, resulting in a total of four treatments. Each treatment consisted of six replicates per rhizosphere soil sample (Figures 1B,D). After collection, the samples were snap-frozen and stored at −80°C for further analysis. Bacterial DNA was isolated from the SC- and CC-treated rhizosphere soil samples using a MagPure Soil DNA LQ Kit (Magen, Guangdong, China) following the protocol described by Shi et al. (2023). Sextuplicate SC- and CC-treated rhizosphere soil samples of the hypervariable region V3–V4 of the bacterial 16S rRNA genes were amplified using universal primer pairs (343F: 5′-TACGGRAGGCAGCAG-3′; 798R: 5′-AGGGTATCTAATCCT-3′). Sequencing was performed on an Illumina NovaSeq6000 with two paired-end read cycles of 250 bases each (Illumina Inc., San Diego, CA, USA), following the protocol described by Shi et al. (2023).

Microbial diversity in the 24 pear tree rhizosphere soil samples was estimated using alpha diversity, which included the Chao1 and Shannon indices (Chao and Bunge, 2002; Hill et al., 2003). The UniFrac distance matrix generated using QIIME2 software (version 2018.2) was used for unweighted UniFrac principal coordinate analysis (PCoA) and phylogenetic tree construction. Sequencing and analysis of the 16S RNA gene amplicons were performed by OE Biotech Co., Ltd. (Shanghai, China). Based on the relative abundance of the soil samples, Spearman’s correlation coefficient was calculated to determine the relationship between bacteria, physicochemical parameters, and metabolites in the sample group at the phylum and genus levels. By default, species with | Spearman Coef | > 0.8 and *p* < 0.01 were displayed on the map. If no matching results were found, a correlation network map was not provided.

### 2.6 UHPLC–MS and data analysis

Pear rhizosphere soil samples (500 mg) under the SC and CC treatments were analyzed using the ACQUITY UPLC I-Class system (Waters Corporation, Milford, USA) combined with a VION IMS QTOF mass spectrometer (Waters Corporation, Milford, USA). The metabolic spectra of the SC- and CC-treated soil samples were analyzed in the ESI positive ion and ESI negative ion modes. An ACQUITY UPLC HSS T3 column (100 mm × 2.1 mm, 1.8 μm) was employed in both positive and negative modes. Water containing 0.1% formic acid and acetonitrile containing 0.1% formic acid were used as mobile agents in Stage A and Stage B. The chromatographic gradient elution procedures were as follows: 0 min, 1% B; 1 min, 30% B; 2.5 min, 60% B; 6.5 min, 90% B; 8.5 min, 100% B; 10.7 min, 100% B; 10.8 min, 1% B; and 13 min, 1% B. The flow rate was 0.35 mL/min, and the column temperature was 45°C. Molecular masses of ion peaks were calculated for all extracts, and data analyses were conducted following the methods outlined by Hu et al. (2018).

### 2.7 GC–MS and data analysis

Rhizosphere soil samples (500 mg) under SC and CC treatments were analyzed using an Agilent 7,890 B gas chromatography system coupled with an Agilent 5,977 B MSD system (Agilent Technologies Inc., CA, USA). A DB-5MSfused-silica capillary column (30 m × 0.25 mm × 0.25 μm, Agilent J & W Scientific, Folsom, CA, USA) was utilized to separate the derivatives. Details of the GC–MS raw data, including chemical derivatization and gas chromatography–mass spectrometry (GC–MS) analyses, were carried out according to previous protocols (Kim et al., 2015).

### 2.8 Data preprocessing and statistical analysis

The matrix was imported into R to perform partial least squares discriminant analysis (PLS-DA) to observe the overall distribution among the soil samples and the stability of the entire analysis process. The metabolites were annotated using the Kyoto Encyclopedia of Genes and Genomes (KEGG) database^1^ (Hill et al., 2003). Univariate analysis (*t*-test) was used to assess statistical significance (*p*-value). Metabolites that differed significantly between any two of the four treated groups were defined as those with a variable importance in the projection value >1.0, *p*-value <0.05, and fold change (FC) ≥ 1.5 or ≤ 0.667. Correlations between differential metabolites (DMs) were analyzed using the Pearson method in R.

## 3 Results

### 3.1 Impact of SC and CC on physicochemical rhizosphere soil parameters and enzyme activity

**Figure 2:**
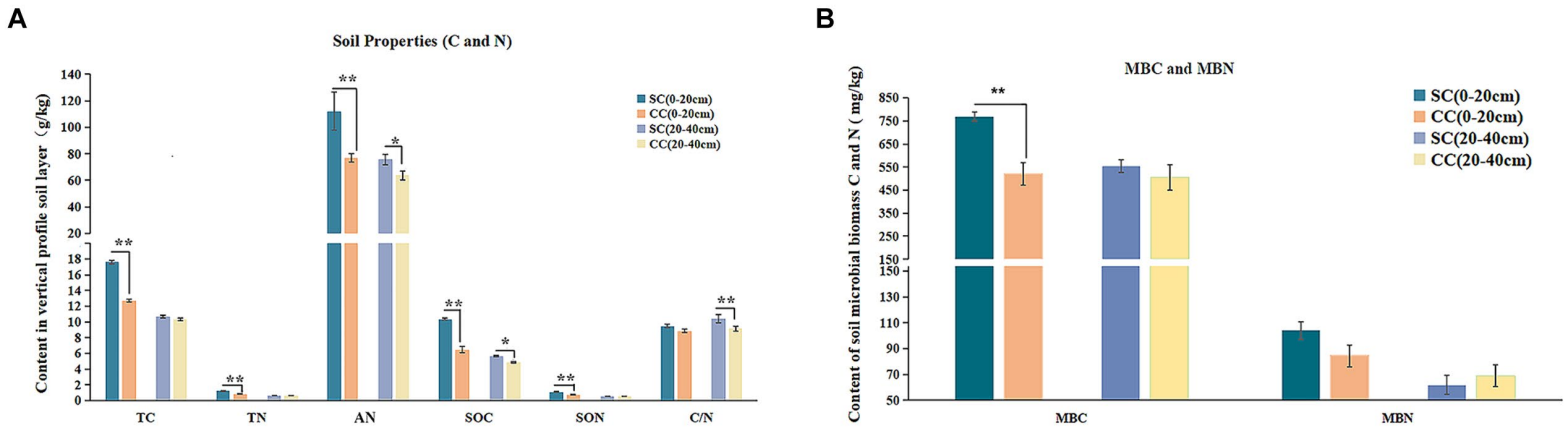
Physicochemical properties, enzyme activities, and microbial biomass between green bristlegrass coverage (SC) mode and clean cultivated mode (CC) in different rhizosphere soil layers in pear orchard. **(A)** The physicochemical properties of carbon and nitrogen. **(B)** The microbial biomass of carbon and nitrogen. Values are the means (*n* = 3) ± the standard deviation of the mean, * indicates statistical differences at *p* < 0.05 and ** indicates statistical extremely differences at *p* < 0.01.

The physicochemical parameters of rhizosphere soil, encompassing EC, pH, TC, TN, SOC, SON, AN, and C:N ratio, were evaluated after a 20-year period in both SC and CC modes within pear orchards. The results demonstrated that SC treatment led to a significant increase in TC and TN levels, elevating from 12.70 g/kg to 17.65 g/kg and from 0.81 g/kg to 1.21 g/kg, respectively, in the 0–20 cm rhizosphere layer compared to CC treatment (Figure 2A) (*p* < 0.001). Moreover, SOC and SON content exhibited substantial increments under SC treatment, with a rise of 59.9 and 16.52% across both the 0–20 cm and 20–40 cm rhizosphere layers, while SON content specifically increased by 49.58% in the 0–20 cm rhizosphere layer. In addition, the C:N ratio in the SC group displayed an increasing trend in both the 0–20 cm and 20–40 cm layers, with a growth rate of 13.84% in the 20–40 cm soil layer (*p* < 0.001). Furthermore, AN content was significantly higher in SC groups compared to CC groups (*p* < 0.05), and SC treatment also resulted in increased AK content in the 0–20 cm rhizosphere layer (Supplementary Table S1).

The application of SC treatments led to a substantial decrease in soil EC, reducing from 669 μs/cm to 481 μs/cm in the 0–20 cm rhizosphere layer and from 666 μs/cm to 541 μs/cm in the 20–40 cm rhizosphere layer. Although SC treatment slightly lowered soil pH, this difference was not statistically significant (Figure 2B). In the 0–20 cm rhizosphere soil layer, the bioavailable fraction of AP experienced a significant long-term decrease under SC treatment. Medium elements such as calcium (Ca) and magnesium (Mg), as well as macroelements including iron (Fe), copper (Cu), manganese (Mn), zinc (Zn), and boron (B), showed significantly higher concentrations under SC treatment (*p* < 0.05) (Supplementary Table S1).

The activities of five soil enzymes (S-SC, S-β-GC, S-PPO, S-POD, and S-CL) in SC and CC soil samples from pear orchards were measured. Notably, both S-β-GC and S-CL are critical enzymes for cellulose degradation. In the SC group, the activity of S-β-GC ranged from 43.11 U/g to 43.31 U/g in the 0–20 cm and 20–40 cm layers, representing increases of 16.20 and 18.41% compared to the CC group, respectively (*p* < 0.001). Similarly, S-CL activity showed increases of 4.02 and 62.49% in the same layers, respectively, compared to the CC group (*p* < 0.001) (Table 1). In addition, S-SC activity, responsible for sucrose breakdown into glucose and fructose, exhibited a similar rising trend as S-β-GC and S-CL activities (*p* < 0.001). Furthermore, SC treatment significantly enhanced S-PPO activity (increasing by 46.49 and 18.84%) during decomposition within the 0–40 cm rhizosphere of pear trees. However, S-POD activity in the SC group was notably decreased by 19.66% in the 20–40 cm rhizosphere layer compared to the CC group (*p* < 0.001). Pearson’s correlation analysis revealed a significant negative correlation between S-β-GC activity and EC value (*r* = 0.956; *p* < 0.05) (Supplementary Figure S3).

**Table 1:** Soil enzyme activities between SC mode and CC mode in different rhizosphere soil layers in pear orchard.

Treatments	Vertical profile (cm)	SC (U/g)	β-GC (U/g)	CL (U/g)	S-PPO (U/g)	POD (U/g)
SC	0–20	119.67 ± 2.02 **	43.11 ± 2.53 *	3441.34 ± 152.40	73.44 ± 1.82 **	26.64 ± 0.68
CC		104.89 ± 7.24	37.10 ± 1.08	3308.23 ± 190.23	50.13 ± 3.06	27.64 ± 0.36
SC	20–40	129.08 ± 2.88 **	43.32 ± 0.82 *	4033.42 ± 131.59 **	73.01 ± 2.80 **	25.41 ± 0.40
CC		97.87 ± 0.90	36.58 ± 1.05	2482.27 ± 46.76	61.44 ± 3.12	30.40 ± 0.59**

S-SC, Soil sucrase; S-β-GC, soil-β-glucosidase; S-CL, soil cellulase; S-PPO, soil polyphenoloxidase; S-POD, soil peroxidase.The table in the same column *indicates significant differences in independent sample t-test results between SC and CC treatments of 0–20 cm soil layer and 20–40 cm soil layer (*p* < 0.05), while **indicates extremely significant differences in independent sample t-test results between different treatments (*p* < 0.01).

### 3.2 Soil microbial biomass and soil microbial community response

In comparison with CC, SC significantly increased MBC and MBN by 47.78 and 23.33% in the 0–20 cm rhizosphere layer; however, no significant differences were observed in the 20–40 cm rhizosphere layer (Figure 2B). Pearson’s correlation analysis revealed a significant positive correlation between MBC and SOC content, as well as between TC, TN, and SON contents (R > 0.95; *p* < 0.05) (Supplementary Figure S3).

**Figure 3:**
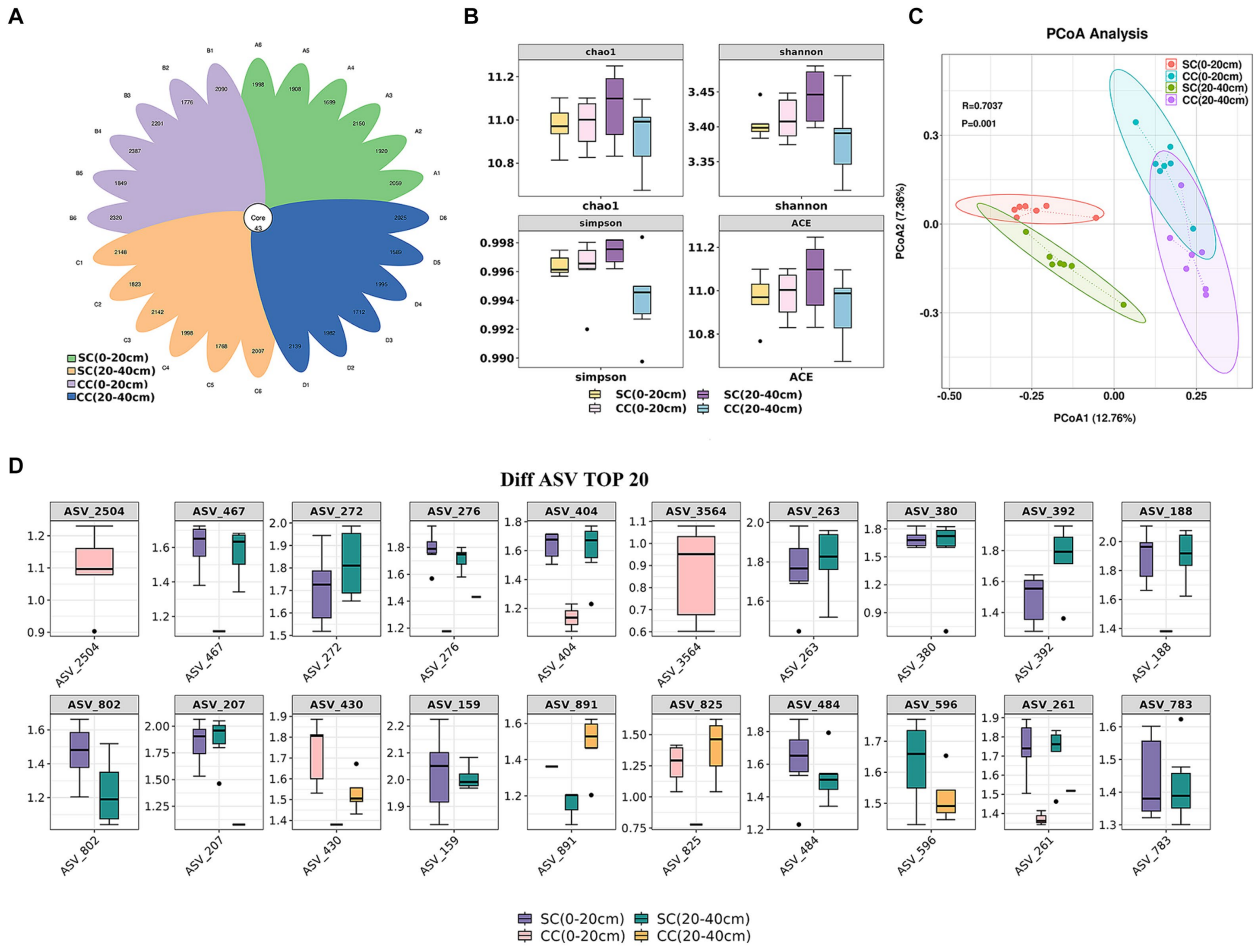
Vertical distribution of rhizosphere soil microbe diversity index between SC mode and CC in different rhizosphere soil layers in pear orchard. **(A)** The Venn diagram of difference AVS with each rhizosphere soil sample between SC mode and CC mode. **(B)** The boxplot of microbe diversity index at the AVS level. **(C)** Principal coordinate analysis (PCoA) diagram of SC and CC based on the Bray–Curtis distance matrix at the AVS level. **(D)** The box plot of the top 20 difference AVS with each rhizosphere soil sample at the AVS level.

Alpha diversity was assessed to analyze bacterial community complexity. Rarefaction curves for Chao and Shannon diversity per compartment reached a saturation plateau, indicating sufficient sequencing depth and reliable data across all samples (Supplementary Figures S1A,B). A total of 78,161–81,596 raw reads were obtained from 16S rRNA gene sequencing. After quality filtering and chimera elimination, the valid tags obtained ranged from 49,309 to 65,053, with 1,632–2,430 amplicon sequence variants (ASVs) per sample (Supplementary Table S2). The flower plot of ASV clustering analysis revealed that the SC (20–40 cm) group exhibited the highest number of ASVs (2,147), followed by the CC (0–20 cm) and SC (0–20 cm) groups, with the CC (20–40 cm) group showing the lowest (1,095 ASVs). The number of common and unique ASVs in different samples is shown in Figure 3A. Forty-three core ASVs were common among rhizosphere soils in both SC- and CC-treated groups. The Simpson index trended as SC (20–40 cm) > SC (0–20 cm) > CC (0–20 cm) > CC (20–40 cm), with Chao1, Shannon, and Simpson indices of SC in the 20–40 cm vertical rhizosphere soil layer significantly higher than those in the other three treatment groups (Figure 3B).

Differences within and between the CC- (control) and SC-treated groups across two vertical distributions were assessed using supervised discriminant analysis via PCoA based on Bray–Curtis distance. PC1 explained 12.76% of the variation, and PC2 explained 7.36%. Bacterial communities in SC-treated soils were distinctly separated from those in CC in both the 0–20 cm and 20–40 cm soil layers (R^2 = 0.52, *p* = 0.001). The disparity in bacterial communities between treatments exceeded that between vertical layers (Figure 3C). The top 20 ASVs with differing abundances, identified via Kruskal–Wallis algorithm analysis (Figure 3D), indicated significant advantages in the CC-treated group for ASV_2504 and ASV_3546 (belonging to Burkholderiales and Candidatus Jorgensen bacteria, respectively) in the 0–20 cm rhizosphere; ASV_272, ASV_404, and ASV_188 (belonging to Gemmatimonadaceae) were more abundant in both the 0–20 cm and 20–40 cm vertical soil layers of the SC treatment group. ASV_276, ASV_263, and ASV_392 (belonging to Bacteroidia) also demonstrated a significant advantage in SC. Moreover, the relative abundances of ASV_159 and ASV_261 (belonging to CCD24) were significantly higher in SC than in CC.

### 3.3 Bacterial community composition and relationships with soil chemical properties at the phylum and genus levels

**Figure 4:**
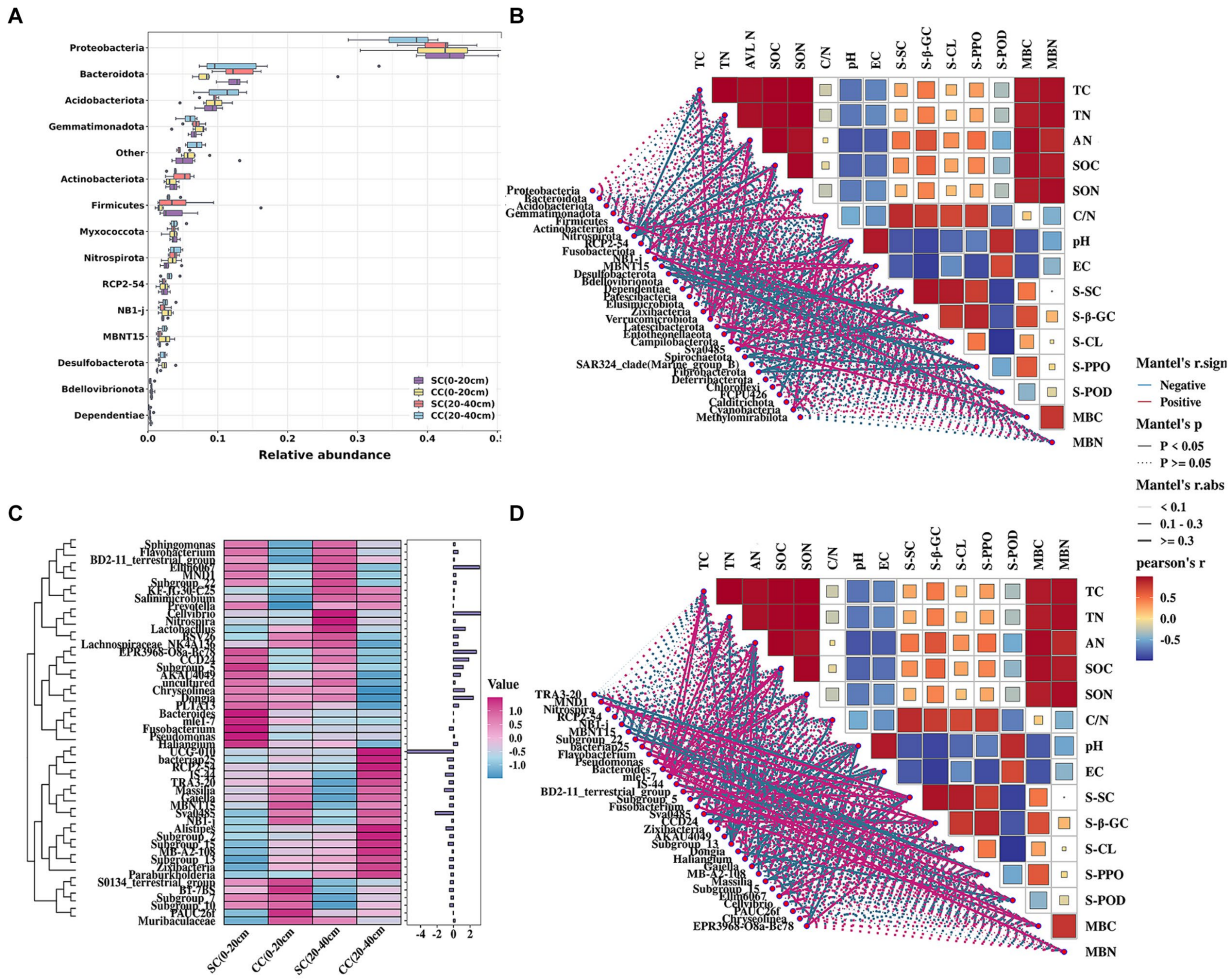
Relative abundance and the correlation between the soil bacterial community structure and physicochemical properties of pear rhizosphere soil between SC mode and CC mode in different rhizosphere soil layers in pear orchard. **(A)** The relative abundance of the top 15 dominant bacteria in phylum level. **(B)** The Spearman correlation heatmap of soil bacteria in phylum level and physicochemical properties. **(C)** The heatmap of relative expression of the top 50 dominant bacteria in genus levels. **(D)** The Spearman correlation heatmap of soil bacteria in genus level and physicochemical properties.

The relative abundance of bacterial communities exhibited similarity to that of dominant bacterial species; however, these dominant bacteria demonstrated distinct preferences in the SC- and CC-treated groups. The bacterial sequences were distributed across 35 phyla, 559 families, and 307 genera. At the phylum level, the dominant bacteria (relative abundance>2%) included Proteobacteria (37–43%), Bacteroidota (11–13%), Acidobacteriota (9–11%), Gemmatimonadota (6–7%), Firmicutes (3–5%), Actinobacteriota (3–5%), Myxococcota (3–4%), Nitrospirota (3–4%), RCP2-54 (2–3%), NB1-j (2–3%), and MBNT15 (2–3%) (Figure 4A). Pear orchards covered by SC exhibited a significant increase in Proteobacteria, Bacteroidetes, Gemmatimonadota, Firmicutes, Actinobacteria, and Fusobacteria and a decrease in Acidobacteriota, Nitrospirota, NB1-j, MBNT15, and Desulfobacterota compared to CC-treated orchards (Figure 4A). At the genus level, analysis of the top 50 genera revealed that SC treatment led to increased relative abundances of *MND1*, *Subgroup_22*, *Pseudomonas*, *mle1-7*, *Sphingomonas*, *BD2-11_terrestrial_group*, *Subgroup_5*, *CCD24*, *AKAU4049*, *Haliangium*, *Dongia*, *Ellin6067*, and *EPR3968-O8a-Bc78* in both rhizosphere layers (0–20 cm and 20–40 cm). Moreover, the relative abundances of *TRA3-20*, *NB1-j*, *bacteriap25*, *IS-44*, *Sva0485*, *Zixibacteria*, *Subgroup_13*, *MB-A2-108*, *Gaiella*, *Massilia*, *Subgroup_15*, and *Cellvibrio* differed in the CC group (Figure 4C).

Pearson’s correlation analysis was conducted to define the correlation between chemical properties factors and the dominant phyla and genera of the bacterial communities between SC and CC soils (|R| > 0.80 and *p* < 0.05). At the phylum level, Nitrospirota, Verrucomicrobiota, Chloroflexi, and Methylomirabilota were the key taxa highly related to soil properties, including TC, SOC, TN, SON, and AN. In addition, the activity of S-PPO was highly associated with Flavobacterium (*r* = 0.967), while S-SC was significantly positively associated with *MND1*, *Subgroup_22*, *BD2-11_terrestrial_group*, and *Ellin6067*. Soil salinization indicators, EC, and pH were significantly correlated with Sva0485 (*r* = 0.982) and Dependentiae (*r* = 0.817) (Figure 4B). Twenty-five genera were highly associated with soil physicochemical parameters (Figure 4D; Supplementary Table S3), soil enzyme activity, and microbial biomass.

**Figure 5:**
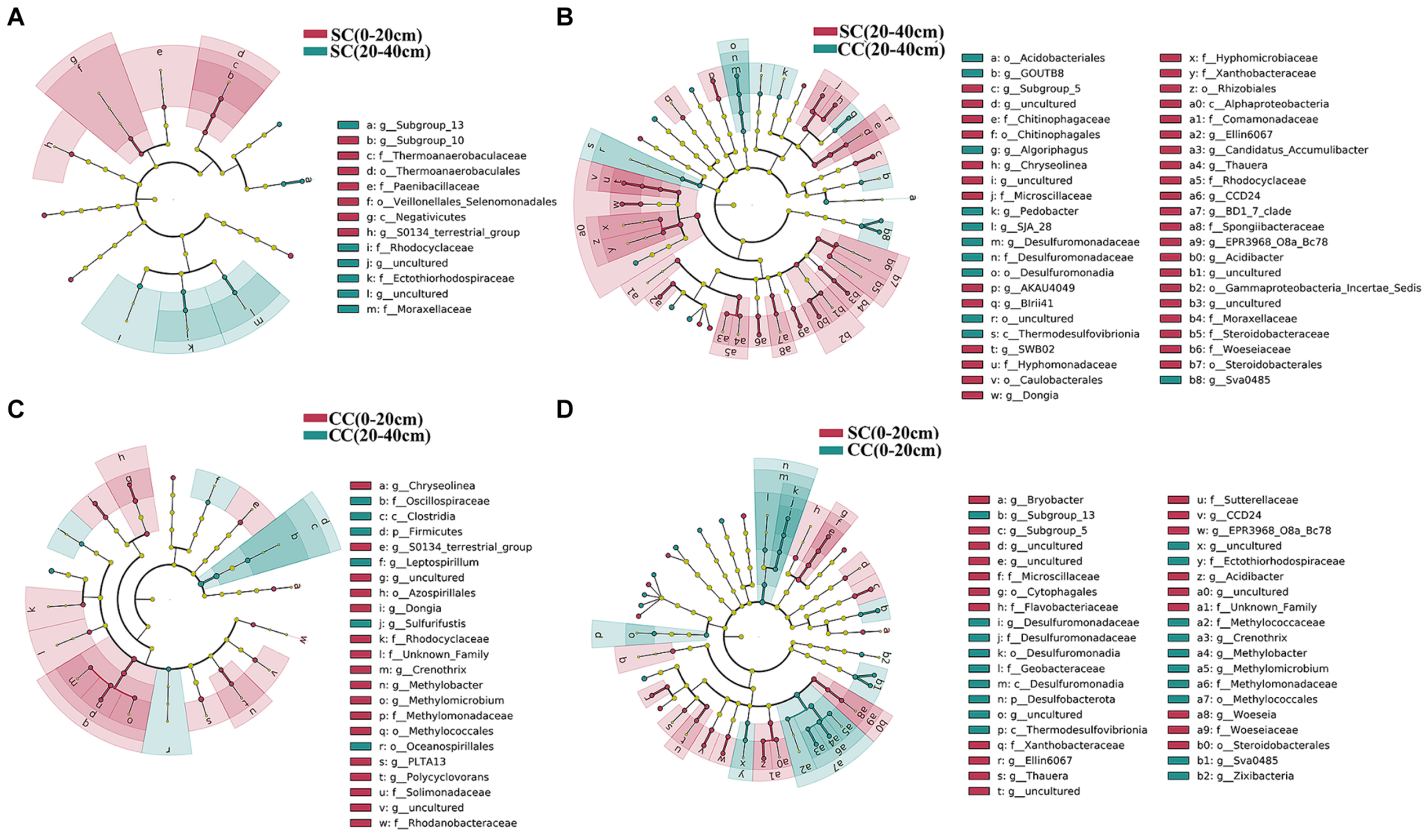
Cladogram plotted from LEfSe comparison analysis indicating the taxonomic representation of statistically and biologically consistent differences of identified biomarkers between SC and CC treatments in different rhizosphere soil layers. **(A)** SC (0–20 cm) vs. SC (20–40 cm); **(B)** SC (0–20 cm) vs. CC (0–20 cm); **(C)** CC (0–20 cm) vs. CC (20–40 cm); **(D)** SC (20–40 cm) vs. CC (20–40 cm). The colored shadows represent trends of the significantly different taxa. The red or blue shading depicts bacterial taxa that were significantly higher or lower in each proportioning of SC and CC treatment whereas species with no significant difference are uniformly colored black.

**Figure 6:**
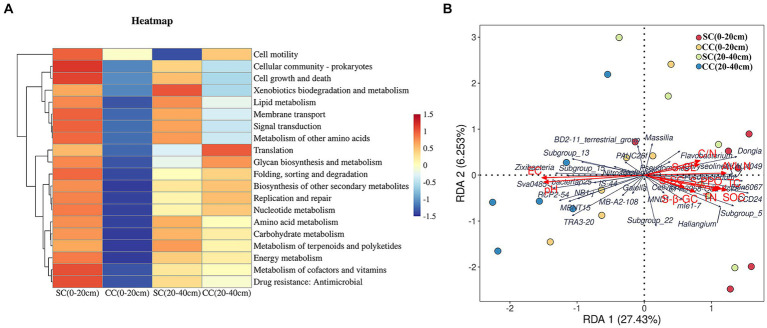
KEGG pathway heatmap of dominant soil bacteria and its relationship with soil properties and dominant soil in genus level between SC and CC treatments in different rhizosphere soil layers. **(A)** KEGG pathway heatmap of dominant soil bacterial in the genus level. **(B)** Redundancy analysis (RDA) of soil physicochemical properties with dominant soil bacterial in the genus level.

We utilized linear discriminant analysis effect size (LEfSe) to identify biomarkers with significant differences between the treated groups (LDA > 2; *p* < 0.05) (Figure 5). This analysis revealed that the minimum number of discriminant clades was associated with soil organic carbon (SC) between the 0–20 cm (seven biomarkers) and 20–40 cm (six biomarkers) soil layers, while the maximum number was found in the SC (49) and conventional carbon (CC) groups (20) at the 20–40 cm soil depth (Figures 5A,B). The orders Veillonellales_Selenomonadales (LDA = 3.1446, *p* = 0.022) and Paenibacillaceae (LDA = 3.020, *p* = 0.037), belonging to Firmicutes, were significantly enriched in the SC (0–20 cm) group. In addition, several genera such as Candidatus_Accumulibacter (LDA = 3.525, *p* = 0.022), *Chryseolinea* (LDA = 3.018, *p* = 0.004), *AKAU4049* (LDA = 3.128, *p* = 0.037), *CCD24* (LDA = 3.492, *p* = 0.004), *Subgroup_5* (LDA = 3.244, *p* = 0.010), *Dongia* (LDA = 3.308, *p* = 0.004), *Ellin6067* (LDA = 3.348, *p* = 0.004), *Acidibacter* (LDA = 3.187, *p* = 0.003), *SWB02* (LDA = 3.024, *p* = 0.010), *Flavobacterium* (LDA = 3.013, *p* = 0.004), and *MND1* (LDA = 3.112, *p* = 0.025) were significantly enriched under the SC (20–40 cm) treatment. In the SC-treated group within the 0–20 cm soil layer, a significant increase in the abundance of 32 biomarkers was observed compared to the CC treatment. Notably, genera such as *Ellin6067*, *Acidibacter*, *Bacteroides*, *EPR3968_O8a_Bc78*, *CCD24*, and *MND1* belonging to the Proteobacteria phylum exhibited higher abundance in SC-treated soils (Figure 5D). Conversely, in the CC-treated group within the 20–40 cm soil layer, biomarkers from the Bacteroidota phylum, including MND1 and IS_44, as well as Sva0485, bacteriap25, and IS_44, were identified as highly abundant (Figure 5B). The KEGG pathway heatmap indicated a greater metabolic potential in SC-treated soils compared to CC soils across both 0–20 cm and 20–40 cm soil layers. This enhanced potential encompassed cellular immunity, cell growth and death, xenobiotic biodegradation and metabolism, lipid metabolism, amino acid metabolism, carbohydrate metabolism, cofactor and vitamin metabolism, and energy metabolism (Figure 6A).

Redundancy analysis (RDA) was conducted to investigate the impact of soil physicochemical factors on bacterial communities. Eleven major environmental factors were found to significantly affect the distribution of rhizosphere bacterial communities in the SC and CC soil samples across both 0–20 cm and 20–40 cm soil layers (*p* = 0.032) (Figure 6B). The eigenvalues of the first two ranking axes of RDA explained 27.68 and 6.40% of bacterial community changes, respectively, which were positively correlated with the activity of S-PPO and the C:N ratio. This suggests that the observed change in microbial community structure may be attributed to the long-term application of SC treatment, leading to alterations in soil physicochemical factors. Furthermore, the C:N ratio and PPO activity emerged as the main factors affecting rhizosphere bacterial community structure (*F* = 1.459, *p* = 0.032), with Ellin6067 showing a positive correlation with S-PPO activity (*r* = 0.60, *p* = 0.001).

### 3.4 Response of soil metabolites to SC and CC in pear orchard rhizosphere

**Figure 7:**
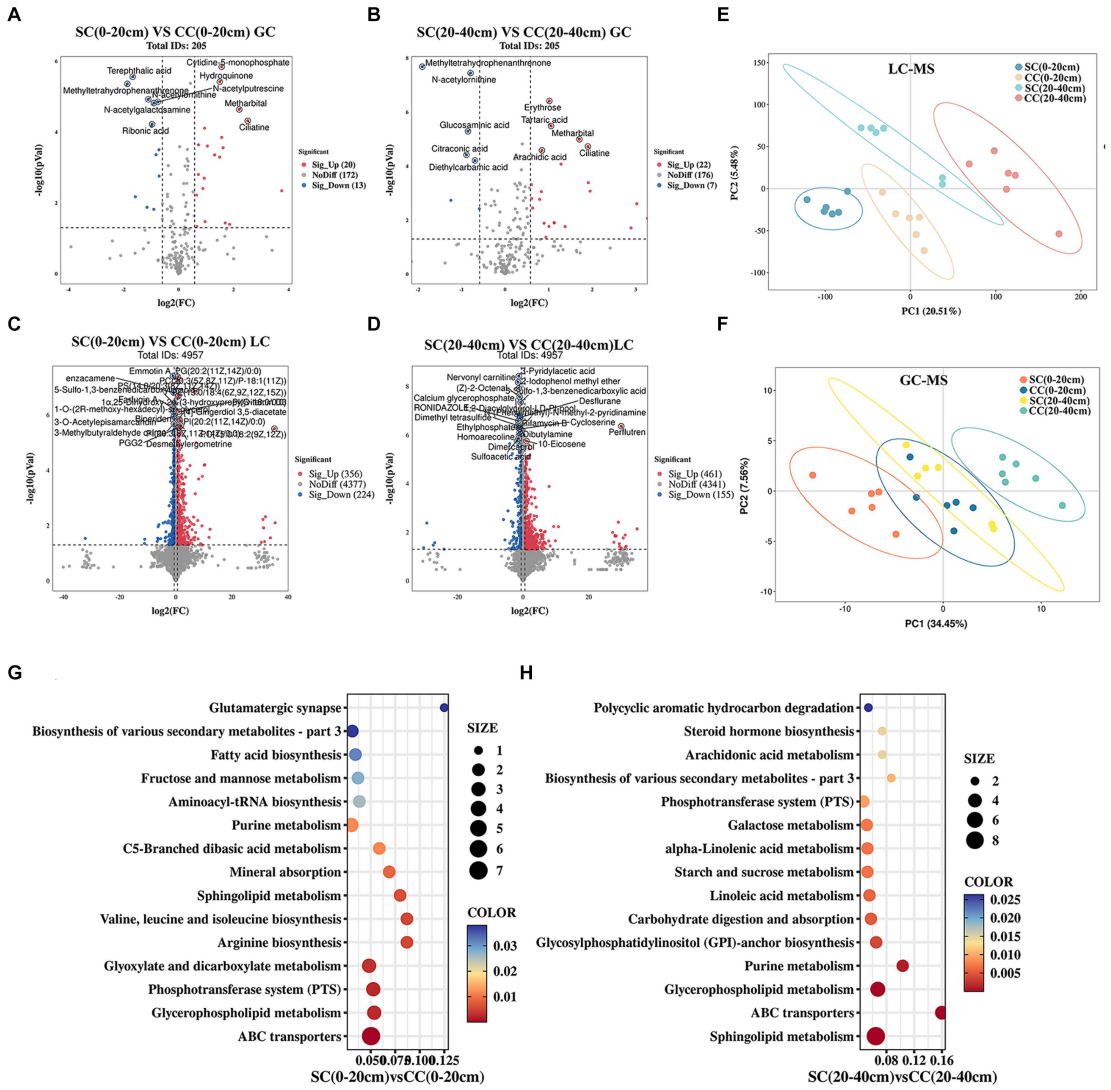
Metabolomic analysis of soil SC and CC treated in different rhizosphere soil layers. **(A,B)** The expression volcano map of DMs up- and downregulate between SC and CC in 0–20 cm and 20–40 cm soil layer by GC–MS. **(C,D)** The expression volcano map of DMs up- and downregulate between SC and CC in 0–20 cm and 20–40 cm soil layer by LC–MS. Green dots represent downregulated metabolites, red dots represent upregulated metabolites, and gray dots represent no-differential metabolites. **(E,F)** Principal coordinate analysis (PCoA) diagram of SC and CC based on the Bray–Curtis distance matrix based on the DMs by LC–MS and GC–MS. **(G,H)** Bubble diagram of the KEGG enrichment of DEMs from SC- and CC-treated groups in 0–20 cm and 20–40 cm soil layer.

To investigate the impact of soil amendments on rhizosphere soil metabolites in pear orchards, we conducted a comprehensive metabolomic analysis using UHPLC–MS/MS and GC–MS/MS platforms. A total of 5,107 known metabolites were identified across two soil layers in both SC and CC treatment groups. Partial least squares discriminant analysis (PLS-DA) highlighted distinct metabolite profiles between SC and CC treatments across different soil layers. The model demonstrated robustness in discerning discriminant metabolites (DMs) from LC–MS and GC–MS data (R2Y = 0.97, Q2Y = 0.69 for LC–MS; R2Y = 0.90, Q2Y = 0.66 for GC–MS), indicating significant differences among treatment groups and soil depths (Figures 7E,F). Volcano plots identified 613 DMs (33 from GC; 580 from LC) and 645 DMs (29 from GC; 616 from LC) between SC and CC groups across soil metabolites (Figures 7A,B,D). Specifically, within the 0–20 cm soil layer, 612 DMs were observed in SC (0–20 cm) vs. CC (0–20 cm), predominantly consisting of fatty acyls (10.93%), glycerophosphoglycerols (7.18%), carboxylic acids and derivatives (6.53%), phenols and prenol lipids (5.71%), amino acids, peptides, and analogs (5.55%), as well as carbohydrates and carbohydrate conjugates (3.92%). In the 20–40 cm soil layer, 645 DMs were identified in SC (20–40 cm) vs. CC (20–40 cm), primarily including amino acids, peptides, and analogs (12.71%), fatty acyls (9.30%), carboxylic acids and derivatives (13.18%), carbohydrates and carbohydrate conjugates (5.89%), and phenols and prenol lipids (2.48%) (Supplementary Tables S5, S6).

KEGG pathway enrichment analysis revealed 50 DMs enriched in pathways related to sphingolipid metabolism, ABC transporters, glycerophospholipid metabolism, purine metabolism, phosphotransferase system, biosynthesis of secondary metabolites, fructose and mannose metabolism, and carbohydrate digestion and absorption (Figures 7G,H; Supplementary Table S7). Compared to CC treatment, SC treatment resulted in significant upregulation of several metabolites, including sucrose (1.48- and 2.52-fold), rhamnose (3.21- and 1.77-fold), N-acetyl-D-glucosamine (0.75- and 1.81-fold), D-(+)-raffinose (2.23- and 0.99-fold), stachyose (2.07- and 0.84-fold), D-mannitol (2.23- and 3.01-fold), D-maltose (4.28- and 2.29-fold), and beta-D-fructose 2,6-bisphosphate (3.42- and 0.53-fold), all belonging to carbohydrates and carbohydrate conjugates. In addition, OAs and derivatives such as pyrrolidonecarboxylic acid (4.59- and 27.70-fold), 2-keto-3-deoxy-D-gluconic acid (4.70- and 5.45-fold), and N-acetyl-L-glutamic acid (2.21- and 3.72-fold) were increased in SC-treated soils across both 0–20 cm and 20–40 cm soil layers. Conversely, organic nitrogen compounds including sphinganine, phytosphingosine, and diethanolamine were significantly downregulated in SC treatments (*p* < 0.05) (Supplementary Table S7).

### 3.5 Assessment of agronomic traits of pear trees (2022–2023): impacts of long-term SC and CC management

Based on the comprehensive evaluation of agronomic traits of pear trees conducted in 2022 and 2023, significant alterations were observed in both the rhizosphere environment and the agronomic characteristics of pear fruits following 20 years of natural grass management (Table 2; Supplementary Table S4). The study revealed that the yield in the SC group was markedly higher than in the CC group, attributable to increased SFW and a higher number of FNP, resulting in yield increments of 62.72 and 35.40%, respectively. Specifically, the average SFW in the SC treatment surpassed that in the CC treatment by 10.01 and 10.34%. In 2022, the significant difference (*p*<0.05) in fruit size between the SC and CC treatments was primarily reflected in the VD, with the SC treatment showing a significant increase of 5.06%, while the difference in LD was not statistically significant (*p* ≥ 0.05). In 2023, the extremely significant difference (*p* < 0.01) in fruit size between the SC and CC treatments was reflected in the LD of fruit size, while there was no significant difference in VD between SC and CC groups (*p* ≥ 0.05).

**Table 2:** Fruit qualities and yields between SC mode and CC mode in different rhizosphere soil layer in pear orchard (2022–2023).

Treatment	Year	SFW (kg)	LD (mm)	VD (mm)	SSC (%)	FNP	Yield (kg)
SC	2023	423.00 ± 66.17 *	91.30 ± 7.28 **	94.00 ± 8.37	12.37 ± 0.59 *	122.10 ± 11.40 **	46247.00 ± 4316.54 **
CC		384.49 ± 30.20	79.92 ± 3.12	89.57 ± 2.58	11.70 ± 0.72	103.90 ± 9.10	34155.98 ± 2990.74
SC	2022	420.89 ± 6.70 *	95.13 ± 6.70	100.59 ± 6.55 *	12.61 ± 0.58 *	114.90 ± 10.65	41348.02 ± 3832.70 **
CC		381.42 ± 40.37	92.97 ± 3.91	95.75 ± 3.20	11.59 ± 0.57	105.60 ± 12.22	25410.84 ± 2941.02

SFW, Single fruit weight; LD, Lateral diameter; VD, Vertical diameter; SSC, Soluble solid content; FNP, Fruit number per plant.The table in the same column *indicates significant differences in independent sample t-test results between SC and CC treatments of 0–20 cm soil layer and 20–40 cm soil layer (*p* < 0.05), while **indicates extremely significant differences in independent sample t-test results between different treatments (*p* < 0.01).

**Figure 8:**
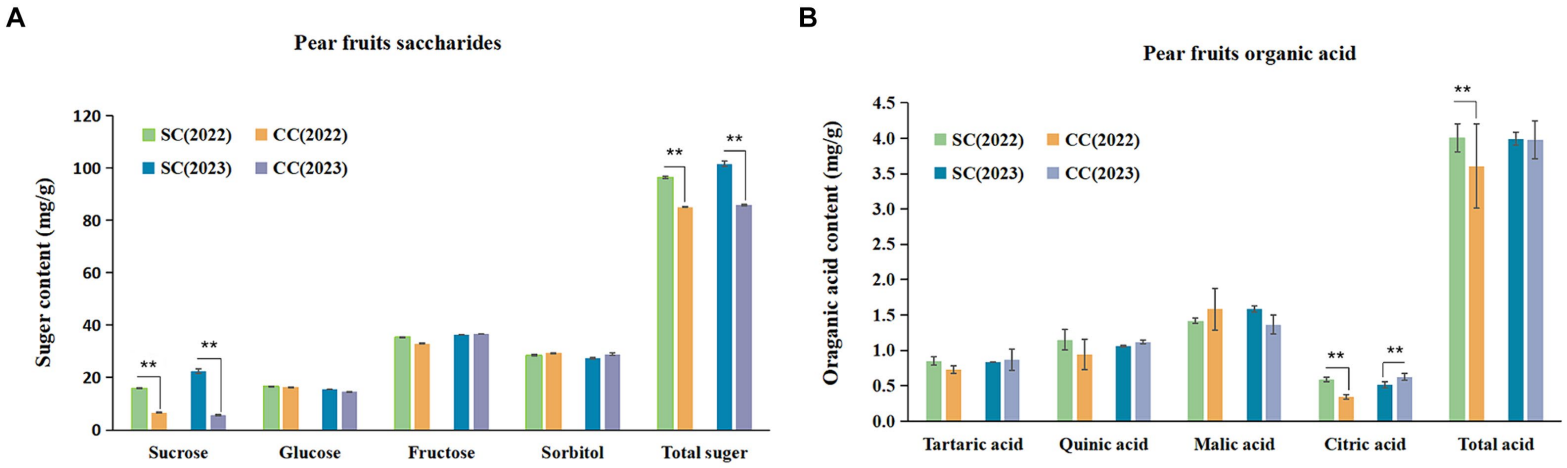
Soluble sugar (SS) composition and organic acid (OA) of pear fruits between SC and CC treatments in different rhizosphere soil layers. **(A)** The soluble sugar (SS) composition and organic acid (OA) of pear fruit. **(B)** The organic acid (OA) of pear fruits. Values are the means (*n* = 3) ± the standard deviation of the mean, * indicates statistical differences at *p* < 0.05, and ** indicates statistical extremely differences at *p* < 0.01.

SSC is a critical indicator of fruit flavor quality and maturity. During the period from 2022 to 2023, the SSC in fruits from the SC treatment exhibited a substantial increase of 8.86 and 5.75% compared to the control (Table 2). SSC primarily comprises TSS and OAs. This study further analyzed specific sugars and acids based on SSC variations induced by SC treatment. The sum of sucrose, glucose, fructose, and sorbitol constituted the TSS content. The ‘Cuiguan’ pear fruits reached maturity, and the SC treatment significantly elevated TSS and sucrose content (*p* < 0.001). The sucrose content in SC-treated pear fruits was approximately 2.43–3.96 times higher than that in CC-treated fruits, with no significant differences observed for other sugars. The advantage in TSS content under SC treatment primarily stemmed from the increased sucrose content (Figure 8A). In 2022, the OA content of fruits in the SC group showed a significant increase of 11.12% compared to the CC group (*p* < 0.001), although this difference was not significant in 2023. Similar to TSS, malic acid was the primary organic acid contributing to the OA content difference (Figure 8B). The results of Pearson’s correlation cluster analysis revealed a positive correlation between indicators such as fruit number per plant, vertical and lateral diameter, soil organic nitrogen (SON), soil organic carbon (SOC), available nitrogen in soil (AN), soil carbon content (C), microbial biomass carbon (MBC), and microbial biomass nitrogen (MBN) and a negative correlation with electrical conductivity (EC) and pH. Moreover, regarding fruit quality, TSS including sucrose and glucose, as well as titratable acidity in OA represented by quinic acid, exhibited a significant correlations with SON, SOC, AN, TC, MBC, MBN, and soluble polyphenol oxidase (S-PPO) in the SC-treated groups (Supplementary Figure S2) (R > 0.9; *p* < 0.001).

### 3.6 Interactions between metabolites and rhizosphere soil PGPMs contributing to agronomic traits in pear fruit

To further investigate PGPMs associated with pear fruit development and nutrient accumulation, we analyzed the correlation between carbon and nitrogen sources in SC and CC rhizosphere soils using Pearson’s correlation. Soil microorganisms and their metabolites were primary sources of these elements. According to Pearson’s correlation cluster analysis, fruit yield indicators, such as fruit number per plant, and vertical and lateral diameter, showed significant positive correlations with TC, TN, SON, SOC, AN, C:N, MBC, and MBN and negative correlations with EC and pH. In addition, in terms of quality, TTS (sucrose, glucose) and OA (quinic acid), which determine the intrinsic quality of fruit, were significantly correlated with TC, TN, SON, SOC, AN, C:N, MBC, MBN, and S-PPO and negatively correlated with EC and pH in SC-treated groups (Supplementary Figure S2) (R > 0.9; *p* < 0.001).

**Figure 9:**
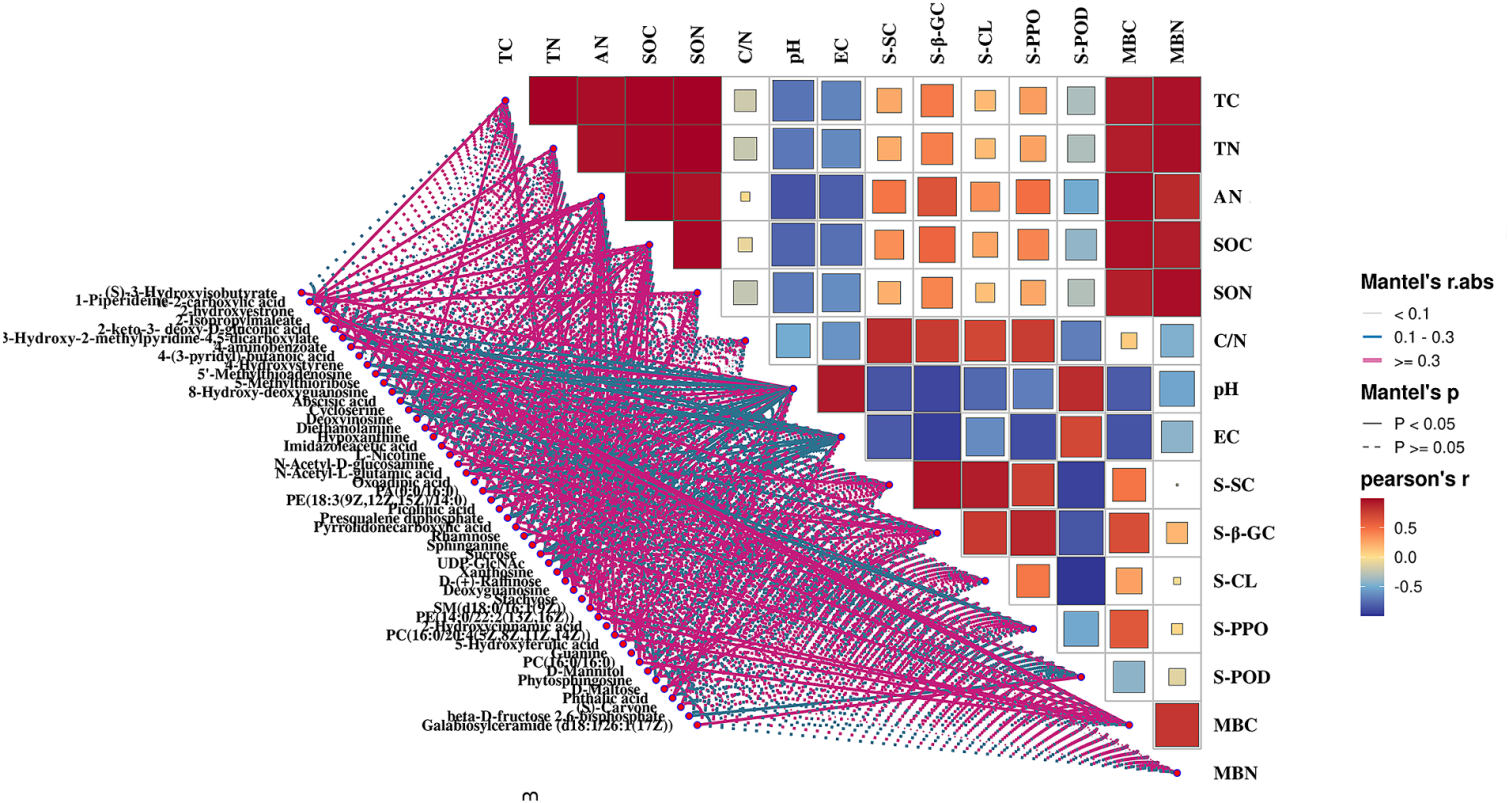
Spearman’s correlation heatmap of soil bacteria in genus level and soil DMs.

To optimize the presence of PGPMs for maximizing both pear yield and quality, we utilized the Mantel test (*p* < 0.05) to examine correlations between microorganisms and their metabolites (Figure 9). The results revealed significant correlations: diethanolamine (*r* = 0.862), N-acetyl-d-glucosamine (*r* = 0.862), D-maltose (*r* = 0.849), and galabiosylceramide (d18:1/26:1(17Z)) (*r* = 0.618) with the bacteria MND1, which also correlated with soil enzyme activities S-β-GC, S-CL, and S-POD. D-mannitol (*r* = 0.876) exhibited a significant correlation with the bacteria NB1-j, which further correlated with S-β-GC activity (*r* = 0.977). Galabiosylceramide (d18:1/26:1(17Z)) (*r* = 0.811), N-acetyl-d-glucosamine (*r* = 0.801), diethanolamine (*r* = 0.680), and D-maltose (*r* = 0.520) correlated with the bacteria IS-44 and significantly correlated with S-SC activity (*r* = 0.742). In addition, metabolites such as 2-hydroxyestrone, 4-hydroxystyrene, imidazoleacetic acid, L-nicotine, presqualene diphosphate, sphinganine, SM (d18:0/16:1(9Z)), PE (14:0/22:2(13Z,16Z)), PC (16:0/20:4 (5Z,8Z,11Z,14Z)), PC (16:0/16:0), phytosphingosine, and phthalic acid showed significant correlations with Subgroup_5 and Sva0485, which in turn significantly correlated with MBC content (*r* = 0.572; *r* = 0.583). The bacteria from Subgroup_15 were found to positively correlate with deoxyinosine (*r* = 0.977), hypoxanthine (*r* = 0.989), oxoadipic acid (*r* = 0.972), D-(+)-raffinose (*r* = 0.929), deoxyguanosine (*r* = 0.977), and guanine (*r* = 0.979), as well as with TC (*r* = 0.640), TN (*r* = 0.618), SOC (*r* = 0.761), SON (*r* = 0.592), and AN (*r* = 0.854). Dongia bacteria showed positive correlations with S-CL (*r* = 0.747) and S-POD (*r* = 0.872) activities, diethanolamine (*r* = 0.836), N-acetyl-d-glucosamine (*r* = 0.728), D-maltose (*r* = 0.712), and galactosylceramide (d18:1/26:1(17Z)) (*r* = 0.990).

## 4 Discussion

### 4.1 Remediation effect of returning natural grass on soil carbon and nitrogen stocks and utilization in pear orchard

CC, where grass is removed through clean tillage, is the main reason for exacerbated soil degradation (Novikova et al., 2013). This study highlights that topsoil degradation in cleared orchards primarily manifests through chemical and biological processes. Over a 20-year period, CC-treated soil exhibited an EC value of 667 μs/cm, categorizing it under moderate saline-alkali conditions (400–800). Conversely, continuous SC treatment effectively mitigated soil salinization by reducing EC values and lowering alkaline soil pH. This suggests that SC application can alleviate the adverse effects of excessive chemical fertilizers in pear orchards. Significant reductions in rhizosphere and bulk soil pH were observed following ryegrass coverage (Wang et al., 2023; Zhou et al., 2023), likely attributed to OA secreted by grass roots (Matocha et al., 2018). In SC-treated groups, OA levels were notably higher compared to CC-treated soil. Further analysis of rhizosphere metabolites indicated substantial upregulation of OAs such as oxoadipic acid, N-acetyl-L-glutamic acid, 2-keto-3-deoxy-D-gluconic acid, and pyrrolidonecarboxylic acid post-SC treatment, suggesting their role in pH reduction. Zhalnina et al. (2018) demonstrated that plant rhizosphere bacteria preferentially utilize aromatic organic acids secreted by plants.

Numerous studies have illustrated that grass roots significantly influence SOC stocks. Water-soluble small molecules from SOC can be absorbed by roots, promoting their growth (Gregory et al.,2022; Gómez et al., 2022). Continuous grass coverage enhances TC and SOC content, contributing to orchard economic benefits (Liu et al., 2021). Despite a decrease in TC to 10.32 g/kg and SOC to 6.46 g/kg after over 20 years of pear fruit harvesting and pruning, SC-treated soil exhibited significant increases in TC, SOC, and C:N ratio by 38.9, 58.9, and 13.84%, respectively. This enhancement correlated significantly with TSS accumulation, fruit size, and yield, underscoring the positive impact of grass coverage on plant productivity. Grass mulching over 2 years improved soil nutrients and organic carbon, thereby enhancing total soluble fruit quality (Lienhard et al., 2013; Dong et al., 2021), a finding corroborated by Homma et al. (2012). While much research has focused on SOC’s impact on soluble solids in fruits, investigations into soluble sugars and OA remain limited. In SC treatment group, the contents of sucrose and glucose in TSS and quinic acid in OA, which determine fruit quality, were significantly correlated with SON, SOC, and C: N ratio in soil. Moreover, fruit yield, linked to soil C, echoed findings from studies on organic carbon addition (Shi et al., 2023).

Research confirms that orchards emit significantly more carbon than other arable lands. Grass coverage enhances soil carbon storage and reduces carbon emissions (Zhao et al., 2022). Decomposing grass, as green manure, releases soil nutrients and undergoes mineralization into inorganic nitrogen by the soil microbiome (Daly et al., 2021). SC treatment increased soil TN and available nitrogen in soil AN, consistent with findings by Zhou et al. (2023) and Wang et al. (2023). Interestingly, different grass varieties have varied effects on AP values in orchards. Unlike CC, hairy vetch (*Vicia villosa* Roth.) coverage notably increased available phosphorus content, while ryegrass (*Lolium perenne* L.) had no significant effect. Green bristlegrass coverage, conversely, reduced available phosphorus levels. The experimental site exhibited high AP, exceeding the normal range (10–20 mg/g), as previously documented. High-phosphorus treatments enhance grass phosphorus absorption and accumulation, potentially mitigating AP accumulation in orchard soils (Kaneko et al., 2008). Therefore, SC emerges as a promising strategy to alleviate soil AP risks in the orchard.

### 4.2 Variations in the diversity and dynamics of rhizosphere soil communities

In comparing microbial diversity between SC and CC treatments across different soil layers, we observed that the highest species richness (ACE, Chao1) and microbial community diversity (Shannon diversity index, *p* < 0.05) occurred in SC-treated soil at the 20–40 cm layer. LEfSe analysis showed that the SC (49) and CC (20) groups in this layer had the highest number of discriminant clades, confirming greater bacterial community diversity in SC treatments. According to β-multivariate statistical analysis, the impact of grass growth on microbial diversity was significantly greater than the effect of vertical distribution within the same treatment. SC treatment increased bacterial population size and altered community composition, consistent with the results of Zhang et al. (2018). At the phylum level, Proteobacteria, Bacteroidetes, Acidobacteria, Gemmatimonadota, Firmicutes, Actinobacteria, Myxococcus, Nitrospirota, RCP2-54, NB1-j, and MBNT15 were dominant bacteria (relative abundance >2%) in pear orchards. SC-treated orchards showed significant increases in Proteobacteria, Bacteroidetes, Gemmatimonadota, Firmicutes, Actinobacteria, and Fusobacteria compared to CC-treated orchards. Firmicutes were highly correlated with soil nutrient content, as noted by Zhao et al. (2022) and Zhang et al. (2022).

The priming effect (PE) of grassland orchards returning ecosystems to exposed topsoil accelerates the process of soil SOC and SON input and decomposition into micromolecular monomers over a short period of time, with soil enzymes and microorganisms playing crucial roles (Guenet et al., 2010). On a large scale, SC treatment positively impacted MBC and MBN, particularly enhancing soil microbial diversity in pear orchards. The regulation of MBC and MBN was observed to be influenced by TC, TN, SOC, SON, and available nitrogen (AN) content (R > 0.95, *p* < 0.05). Gregory et al. (2022) and Xiang et al. (2023) reported that, compared with CC treatment, SC generally increased soil MBC and bacterial abundance irrespective of orchard age. On a smaller scale, the analysis focused on the correlation between the dominant bacteria among the top 30 in soil microbial relative abundance and carbon and nitrogen utilization under SC treatment. Correlation analyses indicated significant positive associations between the relative abundances of Nitrospirota, Verrucomicrobiota, Chloroflexi, Actinobacteria, Cyanobacteria, and Methylomirabilota with the accumulation of TC, TN, SOC, SON, and the C:N ratio. Specifically, the phylum Nitrospirota (Nitrospirae) exhibited notable increases with SOC elevation under ryegrass coverage (Rodríguez-Loinaz et al., 2008). Bacteroides, mle1-7, and Fusobacterium showed positive correlations with SOC, SON, and AN, while MND1, Subgroup_22, BD2-11_terrestrial_group, Ellin6067, and Flavobacterium were associated with S-SC enzymes involved in soil carbon cycling. Prior research highlighted the significant relationship between MND1 and mle1-7 and increased organic carbon in soils (Shi et al., 2023). MND1 plays a critical role in soil carbon and nitrogen cycles, linked to enhanced recalcitrant SOC storage (Hu et al., 2018). Cellvibrio exhibited a significant association with the C:N ratio. Grass, acting as a carbon source, secretes cellulose that facilitates its decomposition into organic carbon sources, thereby enhancing soil carbon levels (Yu et al., 2019). The application of SC treatment resulted in an increased relative abundance of AKAU4049 within both rhizosphere layers (0–20 cm and 20–40 cm), accompanied by a decrease in soil pH. Conversely, a decline in the AKAU4049 genus of the Gemmatimonadota phylum following the introduction of ryegrass suggested optimal soil entropy and nutrient enrichment (Zhou et al., 2023). Variations in AKAU4049 levels may be attributed to differences in soil conditions and grass species utilized.

In the rhizosphere soil treated with SC, the activities of S-SC, S-CL, and S-β-GC enzymes showed significant increases compared to CC treatment. Particularly, S-CL activity exhibited positive correlations with the relative abundance of Firmicutes and Latescibacterota. In agricultural fields practicing multiple crop intercropping, S-CL levels were notably elevated, driven by Firmicutes, Latescibacteria, Gemmatimonadetes, and Acidobacteria, crucial for cellulose decomposition (Zhang et al., 2018). Moreover, S-SC enzyme activity demonstrated correlations with cyanobacteria. Research by Xu et al. (2023) conducted topsoil treatments with artificial cyanobacterial crusts, directly confirming their role in accelerating S-SC enzyme activity recovery.

### 4.3 Integrated analysis of microbial communities and metabolites reveals responses of soil microorganisms

The presence of active microbial communities in the rhizosphere significantly influences soil metabolites, thereby impacting the circulation and metabolism of exogenous nutrients (Cheng et al., 2022). This study examines the interplay between soil metabolites and bacterial communities. Treatment with SC (presumably a soil conditioner or similar) alters the synthesis of various compounds including carbohydrates, phenols, lipids, amino acids, peptides, fatty alcohols, carboxylic acids, and their derivatives. Sucrose, N-acetyl-D-glucosamine, and beta-D-fructose 2,6-bisphosphate are enzymatically catalyzed by NAGase to release nitrogen from the soil, enhancing carbon and nitrogen utilization (Omari et al., 2012). These compounds, synthesized via glycolysis, play crucial roles in plant growth and development (Cheng et al., 2022).

**Figure 10:**
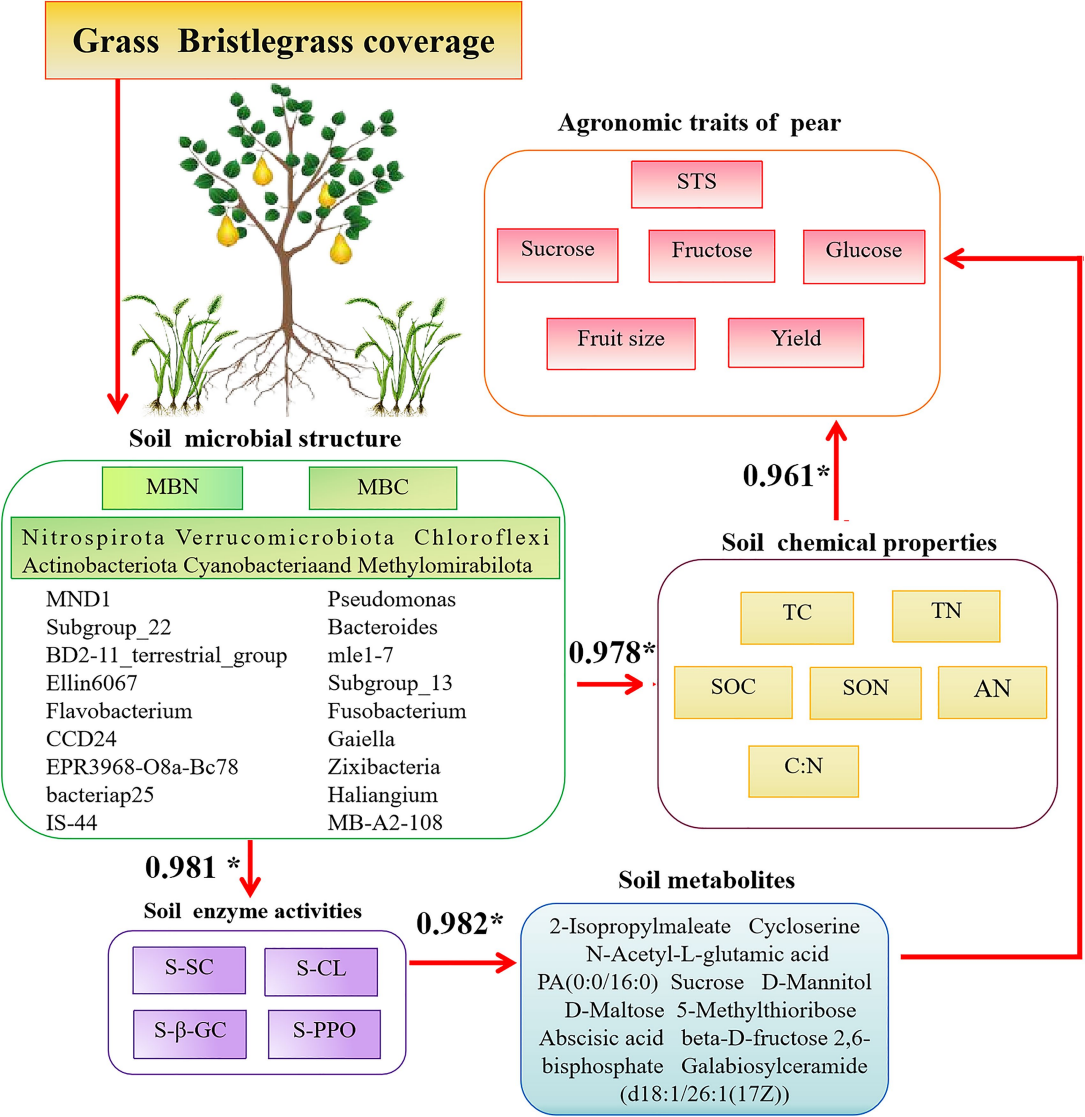
Conceptual diagram of associations among the aboveground part—fruit productivity (yield and quality) and the underground part—the soil physicochemical parameters, enzymes, and microbial community in pear. The values show the R-value by Pearson’s correlation analysis. Up in red arrows indicate increasing and decreasing trends, and only the effects significant at *p* < 0.05 are presented.

Of particular interest, SC promotes the sequestration of organic carbon in soil, leading to increased sucrose accumulation in aboveground pear fruits and rhizospheric secretion. This phenomenon suggests that Bacteroides mle1-7 and Fusobacterium accelerate organic carbon decomposition, thereby enhancing plant carbon absorption, utilization, and synthesis. D-(+)-raffinose, correlated significantly with SOC stock, is part of carbon allocation strategies and may signal stress responses (Li et al., 2020). Conversely, sphinganine, an organic nitrogen compound, is notably downregulated in SC-treated soils. Yang et al. (2021) identified a higher abundance of *Sphingomonas* genus in soil with ryegrass compared to soil without, with SC-treated rhizosphere soils showing significantly elevated Sphingomonas levels. The decrease in sphingosine levels could be attributed to sphingomonas utilizing sphingosine as a carbon and energy source, necessitating its decomposition. Abscisic acid (ABA), a key phytohormone regulating stress responses, is significantly downregulated in SC treatments, potentially promoting pear fruit growth. Studies by Li et al. (2019) suggest that ABA impacts rhizosphere microbial communities and plant physiology, influencing growth characteristics such as height in peanut plants. The relationship between soil bacterial communities and aboveground pear trees is progressively elucidated, highlighting their associations with soil properties and rhizosphere-secreted factors such as metabolites and plant hormones (Figure 10). This study underscores the importance of understanding soil microbial dynamics under prolonged grass cover, although it acknowledges the need for broader geographic observation data to strengthen findings. Future research endeavors should focus on expanding these datasets to enhance their comprehensiveness and robustness.

## 5 Conclusion

Long-term coverage of green bristlegrass in pear orchards has significant potential to enhance carbon and nitrogen storage, thereby improving overall soil nutrient levels, including TC, TN, SOC, SON, and C:N. This practice also promotes increased enzyme activity, such as that of S-SC, S-CL, and S-β-GC. The microbial community within these orchards, encompassing taxa such as Haliangium, Bacteroides, mle1-7, Subgroup_22, Ellin6067, MND1, and Cellvibrio, has exhibited enhanced diversity and functionality. These microorganisms, along with critical metabolites such as sucrose, N-acetyl-D-glucosamine, N-acetyl-L-glutamic acid, rhamnose, UDP-N-acetylglucosamine (UDP-GlcNAc), and D-maltose, play pivotal roles in carbon and nitrogen sequestration. They do so through various metabolic processes centered on carbon, including the synthesis, degradation, and transformation of sucrose and glycolysis during respiration (Figure 10). Furthermore, the coverage of bristlegrass contributes to the distribution of abscisic acid signal molecules in the pear rhizosphere soil. This distribution is primarily driven by the availability of carbon and nitrogen sources in the soil, which can be utilized by the pear fruit. The composition of crop species covered by natural grasses and different soil types also affects the structural distribution of rhizosphere microbial communities, soil metabolites, and enzyme activities, which in turn impact soil carbon sequestration capacity. In future, representative areas with different soil types should be selected for sampling on a broader spatial–temporal scale to comprehensively capture the impact of natural grass cover on the soil microbial environment. This will help to more accurately assess its impact on biodiversity, water conservation, and the carbon and nitrogen cycle of the entire soil ecosystem, leading to more reliable conclusions. Furthermore, based on beneficial bacterial strains related to carbon fixation identified in this study, specific biological functions and mechanisms of action in increasing soil organic carbon were further explored.

## Data Availability

The original contributions presented in the study are publicly available. This data can be found here: https://www.ncbi.nlm.nih.gov/sra/?term=PRJNA1151655.

## Footnotes

^1^ https://www.kegg.jp/

## References

[ref1] Aghajani M. A. Alizadeh A. Rahimian H. (2008). First report of brown patch on bristle basket grass in Iran. Plant Pathol. 57:384. doi: 10.1111/j.1365-3059.2007.01741.x

[ref2] Baer S. G. Bach E. M. Meyer C. K. Du Preez C. C. Six J. (2015). Belowground ecosystem recovery during grassland restoration: South African highveld compared to US tallgrass prairie. Ecosystems 18, 390–403. doi: 10.1007/s10021-014-9833-x

[ref3] Bardgett R. D. Bullock J. M. Lavorel S. Manning P. Schaffner U. Ostle N.. (2021). Combatting global grassland degradation. Nat. Rev. Earth Environ. 2, 720–735. doi: 10.1038/s43017-021-00207-2

[ref4] Chao A. Bunge J. (2002). Estimating the number of species in a stochastic abundance model. Biometrics 58, 531–539. doi: 10.1111/j.0006-341X.2002.00531.x, PMID: 12229987

[ref5] Chen D. Wang Y. Zhang X. Wei X. Duan X. Muhammad S. (2021). Understory mowing controls soil drying in a rainfed jujube agroforestry system in the loess plateau. Agric. Water Manag. 246:106703. doi: 10.1016/j.agwat.2020.106703

[ref6] Cheng H. Yuan M. Tang L. Shen Y. Yu Q. Li S. (2022). Integrated microbiology and metabolomics analysis reveal responses of soil microorganisms and metabolic functions to phosphorus fertilizer on semiarid farm. Sci. Total Environ. 817:152878. doi: 10.1016/j.scitotenv.2021.152878, PMID: 34998744

[ref9004] Daly A. B. Jilling A. Bowles T. M. Buchkowski R. W. Frey S. D. Kallenbach C. M.. (2021). A holistic framework integrating plant-microbe-mineral regulation of soil bioavailable nitrogen. Biogeochemistry 154, 211–229. doi: 10.1007/s10533-021-00793-9 34759436 PMC8570341

[ref7] Debasis M. Snežana A. Panneerselvam P. Manisha C. Ansuman S. Vasić T. (2019). Plant growth promoting microorganisms (PGPMs) helping in sustainable agriculture: current perspective. Int. J. Agric. Vet. Sci. 7, 50–74.

[ref8] Dobosy P. Sávoly Z. Óvári M. Mádl-Szőnyi J. Záray G. (2016). Microchemical characterization of biogeochemical samples collected from the Buda thermal karst system, Hungary. Microchem. J. 124, 116–120. doi: 10.1016/j.microc.2015.08.004

[ref9] Dong Z. Xue Z. Y. Chen Q. Srivas A. K. Hu C. X. (2021). Grass and plastic film mulching pattern improve soil organic carbon Pool, Physical Properties, Fertility and Fruit Quality of Ponkan Orchards. Preprint. doi: 10.21203/rs.3.rs-308925/v1

[ref10] Fan F. Yu B. Wang B. George T. S. Yin H. Xu D.. (2019). Microbial mechanisms of the contrast residue decomposition and priming effect in soils with different organic and chemical fertilization histories. Soil Biol. Biochem. 135, 213–221. doi: 10.1016/j.soilbio.2019.05.001

[ref11] Fang L. F. Shi X. J. Zhang Y. Yang Y. H. Zhang X. L. Wang X. Z.. (2021). The effects of ground cover management on fruit yield and quality: a meta-analysis. Arch. Agron. Soil Sci. 68, 1890–1902. doi: 10.1080/03650340.2021.1937607

[ref12] Gómez J. A. Reyna-Bowen L. Rebollo P. F. Soriano M. A. (2022). Comparison of soil organic carbon stocks evolution in two olive orchards with different planting Systems in Southern Spain. Agriculture 12:432. doi: 10.3390/agriculture12030432

[ref13] Gregory A. S. Joynes A. Dixon E. R. Beaumont D. A. Murray P. J. Humphreys M. W.. (2022). High-yielding forage grass cultivars increase root biomass and soil organic carbon stocks compared with mixed-species permanent pasture in temperate soil. Eur. J. Soil Sci. 73:e13160. doi: 10.1111/ejss.13160

[ref14] Guenet B. Danger M. Abbadie L. Lacroix G. (2010). Priming effect: bridging the gap between terrestrial and aquatic ecology. Ecology 91, 2850–2861. doi: 10.1890/09-1968.1, PMID: 21058546

[ref15] Hill T. C. Walsh K. A. Harris J. A. Moffett B. F. (2003). Using ecological diversity measures with bacterial communities. FEMS Microbiol. Ecol. 43, 1–11. doi: 10.1111/j.1574-6941.2003.tb01040.x 19719691

[ref9002] Homma S. K. Tokeshi H. Mendes L. W. Tsai S. M. (2012). Long-term application of biomass and reduced use of chemicals alleviate soil compaction and improve soil quality. Soil & Tillage Research 120, 147–153. doi: 10.1016/j.still.2012.01.001

[ref16] Hu D. Chen Y. Sun C. Jin T. Fan G. Liao Q.. (2018). Genome guided investigation of antibiotics producing actinomycetales strain isolated from a Macau mangrove ecosystem. Sci. Rep. 8:14271. doi: 10.1038/s41598-018-32076-z, PMID: 30250135 PMC6155160

[ref17] Kaneko M. Kurokawa Y. Qing H. Tanaka H. Suzuki S. Kamata T. (2008). Phosphorus accumulation in soil surface under Japanese lawngrass (*Zoysia japonica* Steud.) pasture. XXI International Grassland Congress & the VIII International Rangeland Congress.

[ref18] Kim Y. M. Nowack S. Olsen M. T. Becraft E. D. Wood J. M. Thiel V.. (2015). Diel metabolomics analysis of a hot spring chlorophototrophic microbial mat leads to new hypotheses of community member metabolisms. Front. Microbiol. 6:132364. doi: 10.3389/fmicb.2015.00209, PMID: 25941514 PMC4400912

[ref19] Ku Y. L. Xu G. Y. Zhao H. Dong T. W. Cao C. L. (2018). Effects of microbial fertilizer on soil improvement and fruit quality of kiwifruit in old orchard. Chin. J. Appl. Ecol. 29, 2532–2540. doi: 10.13287/j.1001-9332.201808.025, PMID: 30182592

[ref20] Kumar B. Dhar S. Paul S. Paramesh V. Dass A. Upadhyay P. K.. (2021). Microbial biomass carbon, activity of soil enzymes, nutrient availability, root growth, and total biomass production in wheat cultivars under variable irrigation and nutrient management. Agronomy 11:669. doi: 10.3390/agronomy11040669

[ref21] Li X. Chu Y. Jia Y. Yue H. Han Z. Wang Y. (2022). Changes to bacterial communities and soil metabolites in an apple orchard as a legacy effect of different intercropping plants and soil management practices. Front. Microbiol. 13:956840. doi: 10.3389/fmicb.2022.956840, PMID: 36003931 PMC9393497

[ref22] Li X. Jousset A. de Boer W. Carrión V. J. Zhang T. Wang X.. (2019). Legacy of land use history determines reprogramming of plant physiology by soil microbiome. ISME J. 13, 738–751. doi: 10.1038/s41396-018-0300-0, PMID: 30368524 PMC6461838

[ref23] Li Q. Yu P. Li G. D. Zhou D. (2016). Grass-legume ratio can change soil carbon and nitrogen storage in a temperate steppe grassland. Soil Tillage Res. 157, 23–31. doi: 10.1016/j.still.2015.08.021

[ref24] Li T. Zhang Y. Liu Y. Li X. Hao G. Han Q. (2020). Raffinose synthase enhances drought tolerance through raffinose synthesis or galactinol hydrolysis in maize and Arabidopsis plants. J. Biol. Chem. 295, 8064–8077. doi: 10.1074/jbc.RA120.013948, PMID: 32366461 PMC7278351

[ref25] Lienhard P. Tivet F. Chabanne A. Dequiedt S. Lelièvre M. Sayphoummie S.. (2013). No-till and cover crops shift soil microbial abundance and diversity in Laos tropical grasslands. Agron. Sustain. Dev. 33, 375–384. doi: 10.1007/s13593-012-0099-4

[ref26] Liu J. F. Li H. F. Yu X. M. Miao A. (2017). Advantageous natural grasses and their nutrition pattern in apple orchards in Taiyi mountainous area. Shandong Agric. Sci. 49, 120–124.

[ref27] Liu Y. P. Mao Y. F. Hu Y. L. Zhang L. L. Yin Y. J. Pang H. L.. (2021). Effects of grass planting in apple orchard on soil microbial diversity, enzyme activities and carbon components. J. Plant Nutr. Fertiliz. 27, 1792–1805. doi: 10.11674/zwyf.2021140

[ref28] Ma X. Chao L. Li J. Ding Z. Wang S. Li F.. (2021). The distribution and turnover of bacterial communities in the root zone of seven Stipa species across an arid and semi-arid steppe. Front. Microbiol. 12:782621. doi: 10.3389/fmicb.2021.782621, PMID: 35003012 PMC8741278

[ref29] Ma X. Li J. Ding F. Zheng Y. Chao L. Liu H.. (2022). Changes of arbuscular mycorrhizal fungal community and Glomalin in the rhizosphere along the distribution gradient of zonal Stipa populations across the arid and semiarid steppe. Microbiol. Spect. 10:e0148922. doi: 10.1128/spectrum.01489-22, PMID: 36214678 PMC9602637

[ref30] Matocha C. J. Karathanasis T. D. Murdock L. W. Grove J. H. Goodman J. Call D. (2018). Influence of ryegrass on physico-chemical properties of a fragipan soil. Geoderma 317, 32–38. doi: 10.1016/j.geoderma.2017.12.004

[ref31] Mikha M. M. Obour A. K. Holman J. D. (2018). Soil nutrients status after fifty years of tillage and nitrogen fertilization. Commun. Soil Sci. Plant Anal. 49, 1953–1975. doi: 10.1080/00103624.2018.1492599

[ref32] Novikova A. F. Konyushkova M. V. (2013). Anthropogenic transformation of soils in the northern Ergeni upland (studies at the first experimental plot of the Arshan’-Zelmen Research Station). Eurasian Soil Sci. 46, 241–253. doi: 10.1134/S106422931303006X

[ref33] Oberholzer H. R. Leifeld J. Mayer J. (2014). Changes in soil carbon and crop yield over 60 years in the Zurich organic fertilization experiment, following land-use change from grassland to cropland. J. Plant Nutr. Soil Sci. 177, 696–704. doi: 10.1002/jpln.201300385

[ref34] Omari K. W. Dodot L. Kerton F. M. (2012). A simple one-pot dehydration process to convert N-acetyl-D-glucosamine into a nitrogen-containing compound, 3acetamido-5-acetylfuran. ChemSusChem 5, 1767–1772. doi: 10.1002/cssc.201200113, PMID: 22887942

[ref9001] Peng S. I. Hui-Li Y. U. Deng-Tao G. Wei S. Xian-Sheng Q. Jin-Yong C.. (2017). Effects of intercropping herbages on carbon source metabolism of soil microbial community in sandy vineyard. Agricultural Research in the Arid Areas 35, 247–254. doi: 10.7606/j.issn.1000-7601.2017.02.40

[ref35] Peters M. K. Hemp A. Appelhans T. Becker J. N. Behler C. Classen A.. (2019). Climate–land-use interactions shape tropical mountain biodiversity and ecosystem functions. Nature 568, 88–92. doi: 10.1038/s41586-019-1048-z, PMID: 30918402

[ref9003] Peterson M. J. Handakumbura P. Thompson A. M. Callister S. J. (2021). Deciphering the microbial and molecular responses of geographically diverse setaria accessions grown in a nutrient-poor soil. PloS one 16:e0259937. doi: 10.1371/journal.pone.0259937 34879068 PMC8654227

[ref36] Qian X. Gu J. Pan H. J. Zhang K. Y. Sun W. Wang X. J.. (2015). Effects of living mulches on the soil nutrient contents, enzyme activities, and bacterial community diversities of apple orchard soils. Eur. J. Soil Biol. 70, 23–30. doi: 10.1016/j.ejsobi.2015.06.005

[ref37] Rodríguez-Loinaz G. Onaindia M. Amezaga I. Mijangos I. Garbisu C. (2008). Relationship between vegetation diversity and soil functional diversity in native mixed-oak forests. Soil Biol. Biochem. 40, 49–60. doi: 10.1016/j.soilbio.2007.04.015

[ref38] Seitz V. A. Chaparro J. M. Schipanski M. E. Wrighton K. C. Prenni J. E. (2023). Cover crop cultivar, species, and functional diversity is reflected in variable root exudation composition. J. Agric. Food Chem. 71, 11373–11385. doi: 10.1021/acs.jafc.3c02912, PMID: 37477948

[ref39] Shi C. H. Wang X. Q. Jiang S. Zhang L. Q. Luo J. (2023). Revealing the role of the rhizosphere microbiota in reproductive growth for fruit productivity when inorganic fertilizer is partially replaced by organic fertilizer in pear orchard fields. Microb. Biotechnol. 16, 1373–1392. doi: 10.1111/1751-7915.14253, PMID: 36965164 PMC10221533

[ref40] Sleiderink J. Deru J. G. C. Weide R. V. D. Eekeren N. V. (2024). Effects of reduced tillage and prolonged cover cropping in maize on soil quality and yield. Soil Tillage Res. 244:106196. doi: 10.1016/j.still.2024.106196

[ref41] Song S. Xiong K. Chi Y. (2022). Grassland ecosystem service and its enlightenment on the revitalization of rural ecological animal husbandry in the rocky desertification area: a literature review. Pol. J. Environ. Stud. 31, 4499–4510. doi: 10.15244/pjoes/149742

[ref42] Sugiura H. Inoue H. Kato O. Tezuka T. Furuya S. Furukawa T. (2017). Changes in soil organic carbon after more than ten years of continuous organic matter application in orchards in Japan. J. Agric. Meteorol. 73, 68–72. doi: 10.2480/agrmet.D-16-00006

[ref43] Wang R. Cao B. Sun Q. Song L. (2020). Response of grass interplanting on bacterial and fungal communities in a jujube orchard in Ningxia, Northwest China. Heliyon 6:e03489. doi: 10.1016/j.heliyon.2020.e03489 32154422 PMC7052399

[ref44] Wang J. Huang J. Zhao X. Wu P. Horwath W. R. Li H.. (2016). Simulated study on effects of ground managements on soil water and available nutrients in jujube orchards. Land Degrad. Dev. 27, 35–42. doi: 10.1002/ldr.2334

[ref45] Wang C. Liang Q. Liu J. Zhou R. Lang X. Xu S.. (2023). Impact of intercropping grass on the soil rhizosphere microbial community and soil ecosystem function in a walnut orchard. Front. Microbiol. 14:1137590. doi: 10.3389/fmicb.2023.1137590, PMID: 36998393 PMC10046309

[ref46] Wang Y. Weng B. Ye J. Wang C. Liu C. Li Y. (2017). Soil organic carbon sequestration potential in nectarine orchards under different reclamation systems. Agric. Sci. Technol. 18, 1192–1195. doi: 10.16175/j.cnki.1009-4229.2017.07.010

[ref047] Wu H. Kong Y. Yao Y. Bi N. Qi L. Fu Z.. (2010). Effects of intercropping aromatic plants on soil microbial quantity and soil nutrients in pear orchard. Scientia Agricultura Sinica 43, 140–150. doi: 10.3864/j.issn.0578-1752.2010.01.017

[ref47] Xiang Y. Chang S. X. Shen Y. Chen G. Liu Y. Yao B.. (2023). Grass cover increases soil microbial abundance and diversity and extracellular enzyme activities in orchards: a synthesis across China. Appl. Soil Ecol. 182:104720. doi: 10.1016/j.apsoil.2022.104720

[ref48] Xu W. W. Zhao Y. Q. Wang N. Zhao Y. (2023). Effects of artificial cyanobacterial crusts on enzyme activities and recovery rate of surface soil in sandy areas. Acta Ecol. Sin. 43, 2856–2864. doi: 10.5846/stxb202204201075

[ref49] Yang Y. Dou Y. Wang B. Wang Y. Liang C. An S.. (2022). Increasing contribution of microbial residues to soil organic carbon in grassland restoration chronosequence. Soil Biol. Biochem. 170:108688. doi: 10.1016/j.soilbio.2022.108688

[ref50] Yang Y. Tong Y. A. Liang L. Y. Li H. C. Han W. S. (2021). Dynamics of soil bacteria and fungi communities of dry land for 8 years with soil conservation management. J. Environ. Manag. 299:113544. doi: 10.1016/j.jenvman.2021.113544, PMID: 34467869

[ref51] Yu X. Borjigin Q. Julin G. Zhigang W. Shuping H. Borjigin N.. (2019). Exploration of the key microbes and composition stability of microbial consortium GF-20 with efficiently decomposes corn Stover at low temperatures. J. Integr. Agric. 18, 1893–1904. doi: 10.1016/s2095-3119(19)62609-2

[ref52] Zakrzewski M. Goesmann A. Jaenicke S. Jünemann S. Eikmeyer F. Szczepanowski R.. (2012). Profiling of the metabolically active community from a production-scale biogas plant by means of high-throughput metatranscriptome sequencing. J. Biotechnol. 158, 248–258. doi: 10.1016/j.jbiotec.2012.01.020, PMID: 22342600

[ref53] Zeng F. Zuo Z. Mo J. Chen C. Yang X. Wang J.. (2021). Runoff losses in nitrogen and phosphorus from paddy and maize cropping systems: a field study in dongjiang basin, South China. Front. Plant Sci. 12:1675121. doi: 10.3389/fpls.2021.675121, PMID: 34447399 PMC8384078

[ref54] Zhalnina K. Louie K. B. Hao Z. Mansoori N. Da Rocha U. N. Shi S.. (2018). Dynamic root exudate chemistry and microbial substrate preferences drive patterns in rhizosphere microbial community assembly. Nat. Microbiol. 3, 470–480. doi: 10.1038/s41564-018-0129-3, PMID: 29556109

[ref55] Zhang Y. Bo G. Shen M. Shen G. Yang J. Dong S.. (2022). Differences in microbial diversity and environmental factors in ploughing-treated tobacco soil. Front. Microbiol. 13:924137. doi: 10.3389/fmicb.2022.924137, PMID: 36171748 PMC9511222

[ref56] Zhang L. Huang W. Xiao W. Hu D. Shao J. Yao B. (2018). Comparison of soil enzyme activity and microbial community structure between rapeseed-rice and rice-rice plantings. Int. J. Agric. Biol. 20, 1801–1808. doi: 10.17957/IJAB/15.0692

[ref57] Zhang C. Zhao X. Liang A. Li Y. Song Q. Li X.. (2023). Insight into the soil aggregate-mediated restoration mechanism of degraded black soil via biochar addition: emphasizing the driving role of core microbial communities and nutrient cycling. Environ. Res. 228:115895. doi: 10.1016/j.envres.2023.115895, PMID: 37054835

[ref58] Zhao Z. Ma Y. Feng T. Kong X. Wang Z. Zheng W.. (2022). Assembly processes of abundant and rare microbial communities in orchard soil under a cover crop at different periods. Geoderma 406:115543. doi: 10.1016/j.geoderma.2021.115543

[ref59] Zhou H. Zhang M. Yang J. Wang J. Chen Y. Ye X. (2023). Returning ryegrass to continuous cropping soil improves soil nutrients and soil microbiome, producing good-quality flue-cured tobacco. Front. Microbiol. 14:1257924. doi: 10.3389/fmicb.2023.1257924, PMID: 37876786 PMC10591219

