# Supplementary material for: Investigating the impact of long-term bristlegrass coverage on rhizosphere microbiota, soil metabolites, and carbon–nitrogen dynamics for pear agronomic traits in orchards
Source: Front Microbiol. 2024 Sep 5;15:1461254. doi: 10.3389/fmicb.2024.1461254 (PMC11411186; doi:10.3389/fmicb.2024.1461254)
Supplement: Supplementary file 1 [file Table_1.docx]

**Table S1.** Soil chemical properties in long term green bristle grass (*Setaria viridis* (L.) P. Beauv.）coverage（SC）mode and clean cultivated (CC) mode in different rhizosphere soil layer.

| Treatments | Vertical profile  (cm) | pH | EC | A P  (mg/kg) | A K  (mg/kg) | EB Ca  (g/kg) | EB Mg  (mg/kg) | A Cu (mg/kg) | A Fe (mg/kg) | A Mn (mg/kg) | A Zn (mg/kg) | A B  (mg/kg) |
| --- | --- | --- | --- | --- | --- | --- | --- | --- | --- | --- | --- | --- |
| SC | 0-20 | 7.83+0.01 | 481.10+8.26 | 50.27+8.47 | 99.64+2.81* | 3.72+0.28* | 372.10+28.30* | 2.36+0.10* | 19.31+0.90* | 12.41+0.51* | 2.42+0.08* | 0.13+0.005* |
| CC |  | 7.89+0.02 | 669.99+28.62** | 75.55+3.35* | 73.76+2.58 | 3.20+0.45 | 319+4.59 | 1.55+0.31 | 15.72+1.827 | 10.33+0.46 | 0.57+0.04 | 0.11+0.005 |
| SC | 20-40 | 7.86+0.02 | 541.40+71.11 | 16.33+1.40 | 85.09+4.20 | 3.35+0.58 | 335.23+5.89 | 1.18+0.05 | 19.29+0.69 | 9.82+0.40 | 1.62+0.02* | 0.08+0.007 |
| CC |  | 7.93+0.06 | 666.00+15.87** | 31.57+1.78* | 88.61+0.21 | 3.42+0.57 | 342.33+5.71 | 1.76+0.35 | 19.99+4.61 | 10.16+0.24 | 0.77+0.02 | 0.09+0.005 |

Note: The values are means ± standard deviations (n = 3). Values followed by different letters differ significantly (Duncan test, p-value <0.05).Abbreviations: potential of hydrogen (pH); electrical conductivity value (EC); A Cu, available copper; A Fe, available iron; AVL K, available potassium; A Mn, available nitrogen; A P, available phosphorus; A Zn, available zinc; EB Ca, exchangeable calcium; EB Mg, exchangeable magnesium; EC, electrical conductivity.
