# Supplementary material for: Investigating the impact of long-term bristlegrass coverage on rhizosphere microbiota, soil metabolites, and carbon–nitrogen dynamics for pear agronomic traits in orchards
Source: Front Microbiol. 2024 Sep 5;15:1461254. doi: 10.3389/fmicb.2024.1461254 (PMC11411186; doi:10.3389/fmicb.2024.1461254)
Supplement: Supplementary file 2 [file Table_2.docx]

| SampleID | Raw_reads | filtered | percentage of input passed filter/% | denoised | merged | non_chimeric | percentage of input non-chimeric | ASV_counts | Total_ASVs |
| --- | --- | --- | --- | --- | --- | --- | --- | --- | --- |
| SC(0-20cm) | 79842 | 73738 | 92 | 70496 | 60848 | 59525 | 75 | 1999 | 21205 |
| CC(0-20cm) | 79506 | 73537 | 92 | 69700 | 58973 | 57559 | 72 | 2024 | 21205 |
| SC(20-40cm) | 80086 | 73783 | 92 | 70270 | 60049 | 58312 | 73 | 2147 | 21205 |
| CC(20-40cm) | 79865 | 73442 | 92 | 70435 | 61673 | 60030 | 75 | 1950 | 21205 |

**Table S2.** Rhizosphere soil microbe diversity index between SC mode and CC mode in different rhizosphere soil

layer in pear orchard.

Note: ASVs, Amplicon Sequence Variants
