# Supplementary material for: Investigating the impact of long-term bristlegrass coverage on rhizosphere microbiota, soil metabolites, and carbon–nitrogen dynamics for pear agronomic traits in orchards
Source: Front Microbiol. 2024 Sep 5;15:1461254. doi: 10.3389/fmicb.2024.1461254 (PMC11411186; doi:10.3389/fmicb.2024.1461254)
Supplement: Supplementary file 3 [file Table_3.docx]

**Table S3.** 25 genus of the bacterial communities significantly correlation between the chemical properties between SC- and CC- soils

| spec | env | r | p | rd | r.sign | pd |
| --- | --- | --- | --- | --- | --- | --- |
| TRA3-20 | S-SC | 0.81031096 | 0.041666667 | >= 0.3 | Positive | P < 0.05 |
| MND1 | S-β-GC | 0.911223879 | 0.041666667 | >= 0.3 | Positive | P < 0.05 |
| NB1-j | S-PPO | 0.976849533 | 0.041666667 | >= 0.3 | Positive | P < 0.05 |
| Subgroup_22 | S-SC | 0.99317693 | 0.041666667 | >= 0.3 | Positive | P < 0.05 |
| bacteriap25 | pH | 0.83195062 | 0.041666667 | >= 0.3 | Positive | P < 0.05 |
| Pseudomonas | EC | 0.511690146 | 0.041666667 | >= 0.3 | Positive | P < 0.05 |
| Bacteroides | MBN | 0.943490265 | 0.041666667 | >= 0.3 | Positive | P < 0.05 |
| mle1-7 | TC | 0.934613331 | 0.041666667 | >= 0.3 | Positive | P < 0.05 |
| IS-44 | S-SC | 0.742175469 | 0.041666667 | >= 0.3 | Positive | P < 0.05 |
| BD2-11_terrestrial_group | C/N | 0.758931559 | 0.041666667 | >= 0.3 | Positive | P < 0.05 |
| Subgroup_5 | MBC | 0.571502684 | 0.041666667 | >= 0.3 | Positive | P < 0.05 |
| Sva0485 | MBC | 0.583260169 | 0.041666667 | >= 0.3 | Positive | P < 0.05 |
| CCD24 | pH | 0.848278003 | 0.041666667 | >= 0.3 | Positive | P < 0.05 |
| Zixibacteria | MBC | 0.887560839 | 0.041666667 | >= 0.3 | Positive | P < 0.05 |
| Cellvibrio | S-CL | 0.136862435 | 0.416666667 | 0.1 - 0.3 | Positive | P ＜ 0.05 |
| AKAU4049 | pH | 0.822992051 | 0.041666667 | >= 0.3 | Positive | P < 0.05 |
| Subgroup_13 | TC | 0.943992325 | 0.041666667 | >= 0.3 | Positive | P < 0.05 |
| Dongia | S-β-GC | 0.182100722 | 0.041666667 | 0.1 - 0.3 | Positive | P < 0.05 |
| Haliangium | pH | 0.810447085 | 0.041666667 | >= 0.3 | Positive | P < 0.05 |
| Gaiella | C/N | 0.86895022 | 0.041666667 | >= 0.3 | Positive | P < 0.05 |
| MB-A2-108 | TC | 0.769350385 | 0.041666667 | >= 0.3 | Positive | P < 0.05 |
| Massilia | S-SC | 0.872454342 | 0.041666667 | >= 0.3 | Positive | P < 0.05 |
| Subgroup_15 | TC | 0.639398351 | 0.041666667 | >= 0.3 | Positive | P < 0.05 |
| Ellin6067 | S-β-GC | 0.916689567 | 0.041666667 | >= 0.3 | Positive | P < 0.05 |
| EPR3968-O8a-Bc78 | pH | 0.825137389 | 0.041666667 | >= 0.3 | Positive | P < 0.05 |
