# Supplementary material for: Investigating the impact of long-term bristlegrass coverage on rhizosphere microbiota, soil metabolites, and carbon–nitrogen dynamics for pear agronomic traits in orchards
Source: Front Microbiol. 2024 Sep 5;15:1461254. doi: 10.3389/fmicb.2024.1461254 (PMC11411186; doi:10.3389/fmicb.2024.1461254)
Supplement: Supplementary file 4 [file Table_4.docx]

**Table S4.** Fruit texture between SC mode and CC mode in different rhizosphere soil layer in pear orchard (2022-2023).

| **Treatment** | **Year** | **Peel breaking force**  **/g** | **Peel strength**  **/g·mm-2** | **Peel toughness**  **/g·s** | **Peel flesh drop brittleness /g·s-1** | **Average pulp firmness/g** | **Pulp fiber index** | **Compactness**  **/g.sec** |
| --- | --- | --- | --- | --- | --- | --- | --- | --- |
| **SC** | 2022 | 2833.92+373.30* | 144.37+19.02* | 1421.01+324.07 | 17061.78+2086.22* | 1117.87+96.83 | 58.70+7.26 | -509.39+158.71 |
| **CC** |  | 2576.25+476.19 | 131.24+24.26 | 1133.97+379.71 | 15614.03+2906.88 | 1296.17+135.59 * | 62.50+3.24 | -592.50+148.94* |
| **SC** | 2023 | 2955.13+274.90 | 150.5414.00 | 1344.91+209.63 | 18001.97+1800.86 | 1208.43+191.04 | 59.10+7.17 | -451.17+155.43 |
| **CC** |  | 2938.75+226.45 | 149.71+11.54 | 1335.97+238.18 | 17818.42+1115.69 | 1345.42+175.33 * | 61.50+5.62 | -610.52+176.93* |

Note: The table in the same column * indicates significant differences in independent sample t-test results between SC- and CC- treatments of 0-20 cm soil layer and 20-40 cm soil layer (P<0.05), while ** indicates extremely significant differences in independent sample t-test results between different treatments (P<0.01).
