# Supplementary material for: Investigating the impact of long-term bristlegrass coverage on rhizosphere microbiota, soil metabolites, and carbon–nitrogen dynamics for pear agronomic traits in orchards
Source: Front Microbiol. 2024 Sep 5;15:1461254. doi: 10.3389/fmicb.2024.1461254 (PMC11411186; doi:10.3389/fmicb.2024.1461254)
Supplement: Supplementary file 5 [file Table_5.docx]

**Table S5.** DMs of between SC mode and CC mode in different rhizosphere soil layer in pear orchard by UHPLC-MS and GC-MS.

| **ID** | **Metabolite name** | **Class** | **P-value** | **log2(FCSC0-20vsCC0-20)** |
| --- | --- | --- | --- | --- |
| 11.20_876.5594m/z | PI(18:1(11Z)/18:3(6Z,9Z,12Z)) | Glycerophospholipids | 7.92E-03 | 35.35 |
| 0.54_743.5470n | PC(15:0/18:2(9Z,12Z)) | Glycerophospholipids | 3.09E-06 | 35.10 |
| 13.53_812.5193m/z | PS(O-16:0/20:2(11Z,14Z)) | Glycerophospholipids | 2.77E-02 | 33.68 |
| 10.03_473.1605m/z | N-Glycolyl-D-glucosamine | Unclassified | 4.78E-03 | 31.91 |
| 11.34_469.3324m/z | alpha-Tocotrienoxyl radical | Prenol lipids | 3.79E-02 | 31.59 |
| 8.58_489.1559m/z | 5'-Deoxy-5-fluorocytidine | 5'-deoxyribonucleosides | 1.20E-02 | 31.26 |
| 10.73_367.2457m/z | DG(8:0/0:0/8:0) | Glycerolipids | 6.00E-03 | 31.02 |
| 5.00_448.2424m/z | PC(11:0/0:0)[U] | Unclassified | 4.05E-02 | 30.45 |
| 5.23_331.1078m/z | Camptothecin | Unclassified | 1.35E-02 | 12.20 |
| 0.54_759.5754n | PE(15:0/22:1(13Z)) | Glycerophospholipids | 2.66E-02 | 11.84 |
| 13.32_755.5443n | PC(16:0/18:3(6Z,9Z,12Z)) | Glycerophospholipids | 6.27E-05 | 10.30 |
| 12.97_744.5531m/z | PE(14:0/22:2(13Z,16Z)) | Glycerophospholipids | 6.45E-05 | 10.15 |
| 12.92_752.5581m/z | PE(18:0/20:3(5Z,8Z,11Z)) | Glycerophospholipids | 1.65E-04 | 9.44 |
| 15.35_910.2380m/z | Malvidin 3-O-(6-O-(4-O-alpha-rhamnopyranosyl-beta-glucopyranoside)-5-O-(6-O-malonyl-beta-glucopyranoside) | Unclassified | 1.02E-02 | 9.31 |
| 9.29_449.2133m/z | alpha-[3-[(Hydroxymethyl)nitrosoamino]propyl]-3-pyridinemethanol | Pyridines and derivatives | 4.83E-02 | 9.26 |
| 9.29_381.2259m/z | CAY10412 | Unclassified | 4.50E-02 | 8.81 |
| 13.01_824.5378m/z | PS(22:2(13Z,16Z)/15:0) | Glycerophospholipids | 2.06E-04 | 8.14 |
| 8.52_437.1637m/z | Sparfloxacin | Quinolines and derivatives | 3.01E-02 | 7.94 |
| 11.24_599.5034m/z | DG(16:0/20:4(8Z,11Z,14Z,17Z)/0:0) | Glycerolipids | 6.51E-03 | 7.84 |
| 11.82_773.5334m/z | PA(14:1(9Z)/24:1(15Z)) | Glycerophospholipids | 9.93E-06 | 7.22 |
| 12.92_793.5968n | PC(20:3(5Z,8Z,11Z)/P-18:1(11Z)) | Glycerophospholipids | 1.38E-07 | 7.15 |
| 10.14_184.0731m/z | L-Alanine n-butyl ester | Unclassified | 1.78E-02 | 7.06 |
| 12.94_734.5690m/z | PC(16:0/16:0) | Glycerophospholipids | 7.08E-04 | 6.93 |
| 11.89_738.5425m/z | PC(18:4(6Z,9Z,12Z,15Z)/P-16:0) | Glycerophospholipids | 2.64E-02 | 6.81 |
| 5.88_255.0661m/z | Phenanthrene-3,4-diol | Unclassified | 3.29E-02 | 6.61 |
| 13.20_790.5367m/z | PE(18:0/22:6(4Z,7Z,10Z,12E,16Z,19Z)(14OH)) | Glycerophospholipids | 9.15E-03 | 6.47 |
| 5.94_227.1181m/z | Metyrapone | Organooxygen compounds | 2.77E-02 | 6.35 |
| 13.32_816.5853m/z | PC(20:3(5Z,8Z,11Z)/P-18:1(9Z)) | Glycerophospholipids | 4.90E-03 | 6.34 |
| 6.75_232.1367m/z | DIHYDROFLAVOPEREIRINE | Unclassified | 1.41E-02 | 5.79 |
| 12.67_601.5187m/z | DG(14:1(9Z)/22:2(13Z,16Z)/0:0) | Glycerolipids | 4.34E-02 | 5.71 |
| 13.32_790.5706m/z | PE(19:1(9Z)/22:4(7Z,10Z,13Z,16Z)) | Glycerophospholipids | 8.58E-03 | 5.70 |
| 4.27_506.1193m/z | Glucoarabin | Unclassified | 3.23E-02 | 5.68 |
| 4.82_213.1024m/z | Reduced pyocyanine | Unclassified | 3.39E-02 | 5.28 |
| 1.29_213.0488m/z | Metsulfovax | Unclassified | 7.36E-03 | 5.09 |
| 12.94_810.5994m/z | PC(20:3(6,8,11)-OH(5)/P-18:1(9Z)) | Unclassified | 6.71E-05 | 5.04 |
| 4.04_212.1184m/z | Trp-P-1 | Indoles and derivatives | 4.05E-02 | 4.93 |
| 0.52_273.1824m/z | 5E,8E,11E-hexadecatrienoic acid | Fatty Acyls | 1.04E-02 | 4.89 |
| 10.10_496.3397m/z | PC(0:0/16:0)[U] | Unclassified | 4.54E-02 | 4.87 |
| 0.77_177.0396m/z | 2-Keto-3-deoxy-D-gluconic acid | Keto acids and derivatives | 6.41E-03 | 4.70 |
| 6.75_279.1328n | N-(1-Deoxy-1-fructosyl)valine | Carboxylic acids and derivatives | 1.07E-02 | 4.69 |
| 1.28_147.0767m/z | Pyrrolidonecarboxylic acid | Carboxylic acids and derivatives | 2.59E-02 | 4.59 |
| 10.33_485.3274m/z | Stoloniferone D | Sterol Lipids | 4.92E-02 | 4.58 |
| 10.87_607.3232m/z | PI(20:1(11Z)/0:0) | Glycerophospholipids | 4.71E-02 | 4.57 |
| 9.88_485.3274m/z | Stoloniferone Q | Sterol Lipids | 4.63E-02 | 4.45 |
| 7.69_318.1170m/z | N1-Amidinostreptamine 6-phosphate | Unclassified | 2.90E-02 | 4.45 |
| 4.88_233.1452n | Hexahomomethionine | Unclassified | 4.67E-02 | 4.37 |
| 1.19_237.0626m/z | Xanthopterin-B2 | Unclassified | 2.11E-02 | 4.32 |
| 6.11_286.1449m/z | 5-Hydroxymethylcimetidine | Unclassified | 2.26E-02 | 4.29 |
| 6.32_215.1347n | Cycloate | Unclassified | 4.30E-02 | 4.28 |
| 4.25_195.0293m/z | Phenylglyoxylic acid | Benzene and substituted derivatives | 1.21E-02 | 4.18 |
| 8.52_313.2293m/z | 4-oxo 2-Nonenal-d3 | Unclassified | 5.24E-03 | 4.10 |
| 9.29_313.2386m/z | (±)12,13-DiHOME | Unclassified | 3.99E-02 | 4.08 |
| 0.79_523.0893m/z | METHYL 7-DESHYDROXYPYROGALLIN-4-CARBOXYLATE | Unclassified | 1.70E-02 | 4.07 |
| 12.97_785.5919n | PE(17:0/22:2(13Z,16Z)) | Glycerophospholipids | 9.26E-05 | 4.03 |
| 8.22_256.0801m/z | Hirsutin | Sulfoxides | 1.81E-02 | 3.94 |
| 9.76_264.1253m/z | Prometryn | Unclassified | 4.63E-02 | 3.91 |
| 291 | D-fructose-1-phosphate | Organooxygen compounds | 4.53E-03 | 3.75 |
| 0.79_208.1005m/z | Ethyl 4-(acetylthio)butyrate | Fatty Acyls | 1.58E-03 | 3.60 |
| 9.74_895.2671m/z | 3'-Hydroxy-3,5,6,7,8,4',5'-heptamethoxyflavone | Unclassified | 4.81E-02 | 3.60 |
| 1.16_485.1515m/z | Maltotriose | Organooxygen compounds | 4.08E-02 | 3.58 |
| 9.13_247.1066n | Malonylcarnitine | Fatty Acyls | 2.51E-02 | 3.49 |
| 11.74_776.5619m/z | Cerebroside C | Unclassified | 1.75E-04 | 3.46 |
| 0.52_126.1026m/z | Phenylhydrazine | Unclassified | 1.07E-02 | 3.42 |
| 4.53_305.0643m/z | (-)-Epigallocatechin | Flavonoids | 3.98E-02 | 3.31 |
| 3.49_322.1018m/z | PC(2:0/0:0)[U] | Unclassified | 6.97E-03 | 3.29 |
| 10.24_496.3398m/z | PC(16:0/0:0)[S] | Unclassified | 4.50E-02 | 3.24 |
| 0.72_147.0653m/z | Rhamnose | Organooxygen compounds | 3.34E-02 | 3.21 |
| 7.36_210.0402m/z | N-formylanthranilic acid | Benzene and substituted derivatives | 1.42E-02 | 3.19 |
| 8.51_312.2299n | (±)13-HpODE | Unclassified | 5.71E-03 | 3.15 |
| 9.07_517.3171m/z | (3beta,17alpha,23R)-17,23-Epoxy-3,29-dihydroxy-27-norlanost-8-ene-15,24-dione | Prenol lipids | 4.30E-02 | 3.14 |
| 5.76_259.1232m/z | Sudan II | Unclassified | 3.02E-02 | 3.13 |
| 9.74_640.3083m/z | POV-PS | Glycerophospholipids | 4.30E-02 | 3.12 |
| 11.91_395.2204m/z | 13,14-dihydro-15-keto-PGF1α | Unclassified | 7.81E-04 | 3.06 |
| 4.60_392.1829m/z | PE(6:0/6:0)[U] | Unclassified | 1.43E-02 | 3.04 |
| 9.80_502.2928m/z | PE(0:0/20:4(5Z,8Z,11Z,14Z)) | Glycerophospholipids | 2.35E-02 | 3.03 |
| 0.52_153.0902n | 4-(beta-Acetylaminoethyl)imidazole | Carboxylic acids and derivatives | 2.69E-03 | 3.03 |
| 11.53_618.3998m/z | Phorbol 12-tiglate 13-decanoate | Unclassified | 3.17E-04 | 2.93 |
| 7.33_318.0620m/z | Adefovir | Imidazopyrimidines | 7.61E-03 | 2.91 |
| 0.72_366.1132n | 2-hydroxyestrone | Steroids and steroid derivatives | 1.88E-02 | 2.89 |
| 1.53_280.0687m/z | 6-Succinoaminopurine | Imidazopyrimidines | 2.12E-03 | 2.85 |
| 1.05_199.0217m/z | cis-1,2-Dihydroxy-1,2-dihydrodibenzothiophene | Unclassified | 2.77E-02 | 2.85 |
| 10.87_483.3120m/z | Propapyriogenin A2 | Unclassified | 4.81E-02 | 2.84 |
| 6.10_329.0999m/z | Cimifugin | Unclassified | 5.55E-04 | 2.79 |
| 1.57_267.0726m/z | Xanthosine | Purine nucleosides | 1.58E-03 | 2.79 |
| 1.65_267.0734m/z | S,S-Dimethyl-beta-propiothetin | Unclassified | 4.90E-03 | 2.77 |
| 9.76_261.1221n | Camelinin | Sulfoxides | 3.95E-02 | 2.77 |
| 1.53_309.0340m/z | Ethidimuron | Unclassified | 4.91E-04 | 2.74 |
| 6.27_364.2263m/z | PC(O-8:0/O-1:0)[U] | Unclassified | 1.35E-03 | 2.71 |
| 11.44_824.5376m/z | PS(15:0/22:2(13Z,16Z)) | Glycerophospholipids | 8.77E-04 | 2.68 |
| 1.73_249.0626m/z | Ara-HX | Purine nucleosides | 4.39E-05 | 2.65 |
| 11.73_395.2207m/z | 13,14-dihydro PGF2α | Unclassified | 8.60E-04 | 2.64 |
| 1.16_233.1132m/z | Succinylproline | Unclassified | 4.44E-02 | 2.63 |
| 3.99_255.0508m/z | 2-(Methylthiomethyl)furan | Heteroaromatic compounds | 5.91E-04 | 2.63 |
| 0.52_111.0919m/z | D-2-Amino-hexano-6-lactam | Unclassified | 3.01E-04 | 2.61 |
| 10.53_621.3020m/z | OHOHA-PA | Glycerophospholipids | 3.83E-02 | 2.61 |
| 2.87_279.0590m/z | Trifluridine | Pyrimidine nucleosides | 6.64E-03 | 2.56 |
| 7.02_281.0997m/z | 1,1'-(Tetrahydro-6a-hydroxy-2,3a,5-trimethylfuro[2,3-d]-1,3-dioxole-2,5-diyl)bis-ethanone | Organooxygen compounds | 1.11E-03 | 2.51 |
| 146 | Ciliatine | Organic phosphonic acids and derivatives | 4.81E-05 | 2.51 |
| 9.75_623.3179m/z | OG-PG | Glycerophospholipids | 4.38E-02 | 2.49 |
| 4.29_285.0615m/z | (1R,6R)-6-hydroxy-2-succinylcyclohexa-2,4-diene-1-carboxylate | Unclassified | 1.73E-02 | 2.40 |
| 4.98_408.2131m/z | PC(5:0/5:0)[U] | Unclassified | 2.61E-02 | 2.40 |
| 0.52_139.1231m/z | 2,6-Dimethylaniline | Benzene and substituted derivatives | 2.03E-04 | 2.39 |
| 12.71_506.1986m/z | O-1,4-α-L-Dihydrostreptosyl-streptidine 6-phosphate | Unclassified | 3.40E-02 | 2.38 |
| 4.57_401.1720m/z | Cortisol | Steroids and steroid derivatives | 2.84E-02 | 2.38 |
| 4.60_394.1977m/z | Diacetylfusarochromanone | Benzopyrans | 2.89E-02 | 2.36 |
| 4.46_181.0134m/z | 4-fumarylacetoacetic acid | Keto acids and derivatives | 6.98E-04 | 2.35 |
| 10.55_451.3215m/z | Isomasticadienonalic acid | Prenol lipids | 4.21E-02 | 2.35 |
| 10.53_553.3149m/z | PG(10:0/10:0)[U] | Unclassified | 3.69E-02 | 2.32 |
| 0.52_145.0971m/z | 1-Piperideine-2-carboxylic acid | Pyridines and derivatives | 1.06E-02 | 2.30 |
| 3.49_343.1728m/z | Istamycin AP | Unclassified | 8.39E-04 | 2.30 |
| 4.51_237.0764m/z | Allyl phenoxyacetate | Benzene and substituted derivatives | 3.82E-02 | 2.29 |
| 3.70_298.0969m/z | 5'-Methylthioadenosine | 5'-deoxyribonucleosides | 1.53E-02 | 2.28 |
| 6.09_313.1259m/z | Glutaminyllysine | Carboxylic acids and derivatives | 7.56E-04 | 2.25 |
| 2.87_257.0769m/z | Glutamyl-Glutamate | Carboxylic acids and derivatives | 3.61E-03 | 2.24 |
| 10.95_469.3325m/z | (22E,24R)-Stigmasta-4,22-diene-3,6-dione | Steroids and steroid derivatives | 3.38E-02 | 2.23 |
| 10.54_423.3258m/z | 5alpha,8alpha-epidioxy-stigmasta-6,9(11),22E-trien-3beta-ol | Unclassified | 4.31E-02 | 2.23 |
| 4.44_314.0215m/z | Liriodenine | Unclassified | 3.95E-02 | 2.21 |
| 8.52_294.2196n | γ- 6(7)-EpODE | Unclassified | 8.74E-03 | 2.21 |
| 1.13_188.0557m/z | N-Acetyl-L-glutamic acid | Carboxylic acids and derivatives | 2.62E-03 | 2.21 |
| 0.52_516.4255m/z | TG(10:0/8:0/8:0) | Glycerolipids | 1.35E-04 | 2.21 |
| 75 | Metharbital | Diazines | 2.31E-05 | 2.21 |
| 10.53_486.3347n | Bassic acid | Steroids and steroid derivatives | 3.76E-02 | 2.19 |
| 10.54_486.3348n | Actinidic acid | Prenol lipids | 2.93E-02 | 2.19 |
| 3.51_296.1000m/z | Cordycepin | Unclassified | 5.56E-04 | 2.18 |
| 1.10_264.0738m/z | 8-hydroxy-2'-deoxy Guanosine | Unclassified | 2.86E-05 | 2.15 |
| 0.52_163.0975m/z | 1-(5-Hydroxy-2-pyrimidinyl)piperazine | Unclassified | 1.46E-02 | 2.14 |
| 2.81_323.0496m/z | Dazomet | Unclassified | 5.71E-03 | 2.12 |
| 0.72_290.0847m/z | Miserotoxin | Unclassified | 1.17E-02 | 2.07 |
| 4.93_374.2288m/z | N-3-oxo-hexadec-11(Z)-enoyl-L-Homoserine lactone | Unclassified | 5.00E-03 | 2.06 |
| 1.61_283.0683m/z | 5-Methylbarbiturate | Unclassified | 1.29E-02 | 2.06 |
| 0.81_130.0977m/z | 4-Guanidinobutanal | Unclassified | 4.03E-02 | 2.04 |
| 2.81_255.0620m/z | N-Carbamoyl-2-amino-2-(4-hydroxyphenyl)acetic acid | Carboxylic acids and derivatives | 5.22E-03 | 2.04 |
| 1.36_264.0738m/z | 8-Hydroxy-deoxyguanosine | Purine nucleosides | 2.88E-03 | 2.03 |
| 0.52_117.0658m/z | N,N'-Diacetylhydrazine | Organonitrogen compounds | 1.87E-02 | 2.03 |
| 10.96_471.3470m/z | Murrayenol | Organooxygen compounds | 3.72E-02 | 2.02 |
| 9.74_355.1959m/z | 1-dimethylarsinoyl-pentadecane | Fatty Acyls | 2.06E-02 | 2.02 |
| 0.72_433.0195m/z | 6-Hydroxyluteolin 6,3'-dimethyl ether 7-sulfate | Polyketides | 1.04E-02 | 2.02 |
| 0.75_190.1075m/z | 3-Isopropenylpentanedioic acid | Fatty Acyls | 1.72E-04 | 2.00 |
| 11.90_451.0865m/z | Chorismate | Carboxylic acids and derivatives | 1.35E-02 | 2.00 |
| 1.44_127.0504m/z | Imidazoleacetic acid | Azoles | 8.28E-03 | 1.99 |
| 15.51_138.0660m/z | 6-Aminonicotinamide | Unclassified | 1.00E-02 | 1.97 |
| 0.52_159.1127m/z | Guvacoline | Unclassified | 9.84E-04 | 1.93 |
| 5.32_211.0355m/z | 4-Hydroxy-3-nitrosobenzamide | Unclassified | 1.41E-02 | 1.91 |
| 0.52_157.0970m/z | Gabaculine | Unclassified | 3.24E-02 | 1.89 |
| 0.52_464.3731m/z | 2β-methoxy-1α,25-dihydroxyvitamin D3 | Unclassified | 1.79E-04 | 1.89 |
| 0.71_559.0255m/z | Endothion | Unclassified | 3.10E-04 | 1.86 |
| 303 | 1,5-anhydroglucitol | Organooxygen compounds | 4.00E-02 | 1.86 |
| 0.72_238.0924m/z | N-Acetyl-D-glucosaminate | Unclassified | 4.22E-02 | 1.85 |
| 3.49_297.1075n | 2-Methylguanosine | Purine nucleosides | 2.04E-03 | 1.85 |
| 12.21_623.3190m/z | PI(20:3(8Z,11Z,14Z)/0:0) | Glycerophospholipids | 2.40E-06 | 1.85 |
| 0.72_349.1318m/z | O-Desmethylquinidine | Unclassified | 3.16E-02 | 1.83 |
| 11.53_632.4157m/z | Synaptolepis factor K1 | Unclassified | 3.49E-05 | 1.81 |
| 12.54_623.3552m/z | Amphibine H | Carboxylic acids and derivatives | 4.57E-06 | 1.75 |
| 10.30_455.2418m/z | PA(0:0/16:0) | Glycerophospholipids | 1.61E-02 | 1.75 |
| 0.79_156.1021m/z | SCOPOLINE | Unclassified | 2.74E-02 | 1.75 |
| 0.75_397.0420m/z | Furo[3,4-b]pyridine-3-carboxylic acid, 5,7-dihydro-2-methyl-4-(3-nitrophenyl)-5-oxo-, 2-hydroxyethyl | Unclassified | 4.14E-02 | 1.74 |
| 1.03_130.0976m/z | (R)-Piperazine-2-carboxamide | Unclassified | 3.84E-02 | 1.73 |
| 1.23_174.1239m/z | Indospicine | Unclassified | 3.32E-03 | 1.73 |
| 5.81_241.1047m/z | 2-Hydroxydecanedioic acid | Hydroxy acids and derivatives | 1.52E-02 | 1.72 |
| 1.13_257.0414m/z | N-phosphocreatinate(2-) | Carboxylic acids and derivatives | 4.40E-02 | 1.72 |
| 0.70_176.0798n | Threonylglycine | Carboxylic acids and derivatives | 2.32E-02 | 1.71 |
| 0.70_452.1377m/z | 6-HydroxyKetanserinol | Unclassified | 2.98E-02 | 1.71 |
| 5.98_153.0057m/z | 1,4-Dithiothreitol | Organooxygen compounds | 1.66E-02 | 1.70 |
| 188 | Uracil | Diazines | 3.68E-02 | 1.70 |
| 0.68_334.0666n | 2-(alpha-D-Galactosyl)-sn-glycerol 3-phosphate | Unclassified | 1.26E-02 | 1.68 |
| 9.27_337.2350m/z | 9,13-dihydroxy-10-octadecenoic acid | Unclassified | 3.31E-02 | 1.65 |
| 0.75_316.1306n | Sorgolactone | Prenol lipids | 2.59E-02 | 1.64 |
| 12.69_298.3469m/z | 8-Isoprostane | Saturated hydrocarbons | 4.63E-05 | 1.62 |
| 407 | Melezitose | Organooxygen compounds | 2.78E-04 | 1.62 |
| 10.43_311.2229m/z | 6-Hydroxy-4,6-dimethyl-3-hepten-2-one | Organooxygen compounds | 1.99E-02 | 1.58 |
| 414 | Alpha tocopherol | Prenol lipids | 1.42E-04 | 1.57 |
| 1.12_212.0531m/z | L-2-Amino-6-oxoheptanedioate | Unclassified | 1.58E-02 | 1.57 |
| 221 | Cytidine-5-monophosphate |  | 1.44E-06 | 1.57 |
| 1.26_281.0527m/z | 9-Riburonosylhypoxanthine | Unclassified | 1.51E-02 | 1.56 |
| 6.70_213.0148m/z | 1,2-Dinitrobenzene | Unclassified | 1.53E-02 | 1.53 |
| 10.61_295.2278m/z | 8,11-Heptadecadienal | Fatty Acyls | 1.01E-02 | 1.52 |
| 0.52_116.1072m/z | Trimethylaminoacetone | Organooxygen compounds | 1.26E-05 | 1.52 |
| 214 | Hydroquinone | Phenols | 3.83E-06 | 1.50 |
| 11.45_623.3189m/z | PKOHA-PG | Glycerophospholipids | 7.96E-05 | 1.50 |
| 0.75_448.1662m/z | METHIONYL-LEUCYLPHENYLALANINE | Unclassified | 2.37E-02 | 1.50 |
| 72 | Benzylalcohol | Benzene and substituted derivatives | 5.10E-04 | 1.49 |
| 6.40_196.0247m/z | 3-Hydroxy-2-methylpyridine-4,5-dicarboxylate | Pyridines and derivatives | 3.02E-03 | 1.49 |
| 0.75_235.0925m/z | 2-(Hydroxymethyl)-3-(acetamidomethylene)succinate | Unclassified | 4.09E-02 | 1.48 |
| 3.94_173.1398m/z | Acetylagmatine | Unclassified | 3.13E-03 | 1.48 |
| 0.72_342.1163n | Sucrose | Organooxygen compounds | 1.33E-02 | 1.48 |
| 0.71_304.0617m/z | Atherospermidine | Unclassified | 4.69E-02 | 1.47 |
| 6.00_313.1259m/z | Lysylglutamine | Carboxylic acids and derivatives | 6.76E-03 | 1.47 |
| 5.27_212.0196m/z | o-Nitrobenzoate | Unclassified | 1.82E-03 | 1.46 |
| 0.52_139.0745n | (Morpholinoimino)acetonitrile | Unclassified | 2.36E-03 | 1.45 |
| 6.06_167.0342m/z | 3-Butynoate | Unclassified | 3.33E-03 | 1.45 |
| 9.29_261.2214m/z | 6-[1]-ladderane hexanol | Fatty Acyls | 4.63E-02 | 1.44 |
| 0.52_457.4360m/z | Pentacosanoylglycine | Carboxylic acids and derivatives | 4.84E-04 | 1.44 |
| 6.09_198.0402m/z | 4-Nitroanisole | Unclassified | 3.98E-03 | 1.43 |
| 4.23_190.1440m/z | 2-Methylpropyl 3-hydroxy-2-methylidenebutanoate | Hydroxy acids and derivatives | 3.33E-02 | 1.43 |
| 11.29_625.3346m/z | PI(20:2(11Z,14Z)/0:0) | Glycerophospholipids | 1.56E-06 | 1.43 |
| 10.26_348.2510m/z | N-pentadecanoyl-L-Homoserine lactone | Unclassified | 3.28E-02 | 1.42 |
| 0.52_116.0819m/z | Dimethylimidazole | Unclassified | 2.08E-02 | 1.41 |
| 3.34_191.0808n | Dihydro-6-isopropyl-2,4-dimethyl-4H-1,3,5-dithiazine | Azacyclic compounds | 2.03E-03 | 1.38 |
| 4.03_334.1124m/z | N2,N2-Dimethylguanosine | Purine nucleosides | 3.47E-03 | 1.37 |
| 11.53_343.1574m/z | CP 339818 | Unclassified | 1.78E-05 | 1.37 |
| 0.52_226.1555m/z | Pilocarpine | Unclassified | 1.98E-02 | 1.36 |
| 0.68_274.0455n | 1-Deoxy-D-altro-heptulose 7-phosphate | Unclassified | 9.90E-03 | 1.35 |
| 10.33_557.2734m/z | PI(15:0/0:0) | Glycerophospholipids | 3.41E-02 | 1.34 |
| 1.04_479.1019m/z | 1,4-DPCA | Unclassified | 6.18E-03 | 1.34 |
| 10.67_657.3249m/z | POB-PI | Glycerophospholipids | 2.55E-04 | 1.32 |
| 0.52_169.0970m/z | Acetaminophen | Phenols | 1.11E-02 | 1.32 |
| 1.57_239.0670m/z | (±)-2-(1-Methylpropyl)-4,6-dinitrophenol | Phenols | 8.56E-04 | 1.32 |
| 10.92_357.1884m/z | trans-p-Menthane-7,8-diol 7-glucoside | Prenol lipids | 8.28E-06 | 1.32 |
| 372 | Elaidic acid | Fatty Acyls | 4.41E-04 | 1.31 |
| 5.18_191.0915m/z | Dambonitol | Organooxygen compounds | 3.34E-02 | 1.31 |
| 5.01_235.0609m/z | Austdiol | Azaphilones | 4.55E-02 | 1.30 |
| 0.71_439.0761m/z | 5'-Butyrylphosphouridine | Unclassified | 3.12E-02 | 1.29 |
| 10.32_299.2228m/z | 2,5-Dimethyl-3-propylpyrazine | Diazines | 4.31E-03 | 1.29 |
| 0.75_231.0978m/z | Droxidopa | Carboxylic acids and derivatives | 3.77E-02 | 1.28 |
| 0.72_425.0609m/z | O-Desmethyloxotolrestat sulfate | Unclassified | 2.63E-02 | 1.28 |
| 11.53_322.1423m/z | Amabiline | Unclassified | 1.04E-02 | 1.28 |
| 1.20_202.0824m/z | Asparaginyl-Serine | Carboxylic acids and derivatives | 3.41E-02 | 1.27 |
| 0.52_125.1074m/z | benzylazanium | Unclassified | 2.44E-03 | 1.26 |
| 0.75_183.0567n | Choline sulfate | Unclassified | 7.53E-03 | 1.26 |
| 8.40_301.2021m/z | 11-keto pentadecanoic acid | Fatty Acyls | 4.38E-02 | 1.26 |
| 6.84_241.1776m/z | Neostigmine | Benzene and substituted derivatives | 1.92E-02 | 1.24 |
| 6.26_374.2548m/z | Undecanoylcarnitine | Fatty Acyls | 4.75E-03 | 1.23 |
| 5.07_119.0706m/z | Methyl(S)-3-hydroxybutyrate | Unclassified | 1.94E-02 | 1.23 |
| 3.48_232.1545m/z | Butyrylcarnitine | Fatty Acyls | 4.53E-02 | 1.23 |
| 10.02_311.2229m/z | 12-heptadecynoic acid | Fatty Acyls | 2.71E-02 | 1.22 |
| 11.53_621.3200m/z | Desmethylergometrine | Unclassified | 2.80E-06 | 1.21 |
| 12.73_436.2856m/z | N-oleoyl methionine | Fatty Acyls | 1.48E-04 | 1.21 |
| 9.27_499.2152m/z | Iridodial glucoside tetraacetate | Prenol lipids | 3.50E-02 | 1.21 |
| 9.94_375.1780m/z | [4]-Gingerdiol 3,5-diacetate | Phenols | 1.41E-06 | 1.20 |
| 4.90_206.0817m/z | 3,4-Dihydro-4-[(5-methyl-2-furanyl)methylene]-2H-pyrrole | Heteroaromatic compounds | 4.72E-02 | 1.20 |
| 0.52_287.1983m/z | alpha-Caryophyllene alcohol acetate | Carboxylic acids and derivatives | 3.93E-02 | 1.19 |
| 11.53_475.2326m/z | rac-5,6-Epoxy-retinoyl-beta-D-glucuronide | Prenol lipids | 5.26E-06 | 1.19 |
| 12.09_298.3466m/z | 10,14-Dimethyl-1-octadecene | Fatty Acyls | 5.78E-06 | 1.17 |
| 8.50_335.2193m/z | 8-HpODE | Fatty Acyls | 9.56E-03 | 1.16 |
| 6.26_294.2430m/z | 19-Noretiocholan-3b-ol-17-one | Unclassified | 9.59E-03 | 1.16 |
| 0.51_152.0564m/z | 3,3-Dimethyl-1,2-dithiolane | Dithiolanes | 6.78E-04 | 1.15 |
| 8.09_413.9748m/z | Quinoline yellow | Indanes | 1.22E-05 | 1.13 |
| 1.35_266.0885m/z | 5'-Dehydroadenosine | Unclassified | 7.09E-03 | 1.13 |
| 5.17_333.0723m/z | Phenylacetothiohydroximate | Unclassified | 3.91E-02 | 1.13 |
| 7.57_646.3790m/z | Hydroxyhomodestruxin B | Peptidomimetics | 2.30E-03 | 1.12 |
| 7.11_350.2173m/z | 1,8-Epoxy-p-menthan-4-ol glucoside | Prenol lipids | 6.64E-03 | 1.12 |
| 6.33_456.2256m/z | NBD-FTY720 phenoxy | Unclassified | 3.71E-04 | 1.10 |
| 0.52_129.1023m/z | L-Lysine 1,6-lactam | Unclassified | 5.54E-03 | 1.10 |
| 3.59_240.0743n | Dinoterb | Unclassified | 9.72E-03 | 1.10 |
| 7.98_585.3532m/z | PA(12:0/14:1(9Z)) | Glycerophospholipids | 5.63E-03 | 1.09 |
| 10.87_611.3185m/z | OS-PG | Glycerophospholipids | 1.69E-03 | 1.08 |
| 7.41_604.3533m/z | Presqualene diphosphate | Prenol lipids | 6.73E-05 | 1.08 |
| 11.53_584.3349n | PI(P-18:0/0:0) | Glycerophospholipids | 8.55E-06 | 1.08 |
| 6.21_299.0261m/z | meso-Tartaric acid | Unclassified | 1.24E-02 | 1.07 |
| 0.52_185.1282m/z | 4-(1-hydroxy-2-(methylamino)ethyl)phenol | Unclassified | 5.88E-03 | 1.07 |
| 10.96_387.1990m/z | Fortimicin B | Unclassified | 1.43E-05 | 1.07 |
| 11.53_531.2953m/z | Physapubescin | Steroids and steroid derivatives | 5.30E-06 | 1.06 |
| 6.17_796.4539m/z | PS(14:0/20:3(8Z,11Z,14Z)) | Glycerophospholipids | 5.48E-08 | 1.06 |
| 11.53_586.3505n | PI(O-18:0/0:0) | Glycerophospholipids | 7.48E-07 | 1.06 |
| 5.17_411.0886m/z | Zileuton O-glucuronide | Organooxygen compounds | 2.28E-02 | 1.06 |
| 7.57_646.0441m/z | UDP-GlcNAc | Pyrimidine nucleotides | 2.12E-04 | 1.05 |
| 155 | Glucosamine | Organooxygen compounds | 2.49E-04 | 1.05 |
| 2.15_265.1176n | N6-Methyl-2'-deoxyadenosine | Unclassified | 6.34E-03 | 1.05 |
| 0.52_144.1383m/z | (Z)-2-Octenal | Unclassified | 7.54E-05 | 1.05 |
| 15.51_115.0867m/z | Ornithine | Carboxylic acids and derivatives | 1.44E-02 | 1.04 |
| 7.54_631.3689m/z | Goshonoside F4 | Prenol lipids | 3.14E-06 | 1.04 |
| 11.53_207.1016m/z | Dhelwangin | Organooxygen compounds | 2.94E-05 | 1.03 |
| 11.54_583.3278m/z | Trichodermin | Unclassified | 4.89E-04 | 1.03 |
| 6.75_542.3218m/z | SLF | Unclassified | 2.41E-02 | 1.03 |
| 1.60_200.0544m/z | Clavulanate | Carboxylic acids and derivatives | 2.94E-03 | 1.02 |
| 11.85_298.3468m/z | 10-Eicosene | Unsaturated hydrocarbons | 4.63E-04 | 1.02 |
| 5.17_275.1137m/z | Ethyl (S)-3-hydroxybutyrate glucoside | Fatty Acyls | 2.67E-02 | 1.01 |
| 0.79_150.0411m/z | 2-Amino-3-hydroxypropanoic acid | Carboxylic acids and derivatives | 2.61E-02 | 1.00 |
| 12.84_298.3469m/z | 9Z-Eicosene | Fatty Acyls | 3.81E-05 | 1.00 |
| 0.54_131.0352m/z | 2-Propenyl propyl disulfide | Allyl sulfur compounds | 2.50E-04 | 0.99 |
| 5.17_479.0761m/z | ALIZARIN | Unclassified | 2.97E-02 | 0.99 |
| 0.69_354.0628m/z | N-(6-Oxo-6H-dibenzo[b,d]pyran-3-yl)maleamic acid | Unclassified | 1.07E-04 | 0.98 |
| 5.98_708.4013m/z | PE(13:0/18:4(6Z,9Z,12Z,15Z)) | Glycerophospholipids | 1.97E-07 | 0.98 |
| 9.99_255.1601m/z | Cyclohexane-1-carboxylate | Carboxylic acids and derivatives | 3.06E-02 | 0.98 |
| 0.51_143.1178m/z | Ne-Methyl-L-lysine | Unclassified | 6.12E-03 | 0.97 |
| 7.67_286.0469m/z | Cyanidin | Flavonoids | 1.73E-02 | 0.97 |
| 10.97_625.3346m/z | Physapruin B | Benzopyrans | 2.81E-05 | 0.97 |
| 11.53_411.0939m/z | Indolylmethylthiohydroximate | Unclassified | 2.19E-02 | 0.97 |
| 353 | Palmitoleic acid | Fatty Acyls | 3.36E-02 | 0.96 |
| 0.72_390.1967m/z | Clavamycin F | Unclassified | 1.75E-02 | 0.96 |
| 7.80_619.1685m/z | Pelargonidin 3-(6''-acetylglucoside)-5-glucoside | Unclassified | 1.41E-04 | 0.96 |
| 8.09_297.0472m/z | Quinalphos | Unclassified | 5.11E-05 | 0.96 |
| 12.65_452.2713m/z | Dibekacin | Unclassified | 3.76E-05 | 0.96 |
| 9.45_327.2179m/z | 9-hydroperoxy-12,13-epoxy-10-octadecenoic acid | Fatty Acyls | 3.04E-02 | 0.95 |
| 5.07_204.1000n | Diethyl (2R,3R)-2-methyl-3-hydroxysuccinate | Unclassified | 1.99E-02 | 0.95 |
| 10.29_502.2927m/z | PE(0:0/20:4(8Z,11Z,14Z,17Z)) | Glycerophospholipids | 1.53E-03 | 0.95 |
| 39 | L-threonine | Carboxylic acids and derivatives | 7.82E-05 | 0.95 |
| 0.77_158.0580n | 2-Isopropylmaleate | Fatty Acyls | 3.34E-03 | 0.94 |
| 262 | N-acetyl-d-mannosamine | Organooxygen compounds | 2.01E-03 | 0.94 |
| 10.98_417.2096m/z | Spironolactone | Steroids and steroid derivatives | 1.02E-04 | 0.93 |
| 0.51_104.1070m/z | Cyclopentanol | Unclassified | 2.64E-03 | 0.92 |
| 6.16_407.2436m/z | Annoglabasin C | Prenol lipids | 4.60E-06 | 0.92 |
| 10.83_313.2386m/z | 11-Cyclohexylundecanoic acid | Fatty Acyls | 9.47E-03 | 0.91 |
| 5.45_470.2366n | (R)-1-O-[b-D-Glucopyranosyl-(1->6)-b-D-glucopyranoside]-1,3-octanediol | Fatty Acyls | 2.25E-05 | 0.91 |
| 11.53_451.0865m/z | Prephenate | Keto acids and derivatives | 4.43E-02 | 0.91 |
| 4.10_153.1155n | 5-Ethyl-2-methyl-4-propyloxazole | Azoles | 3.91E-03 | 0.90 |
| 6.26_352.2460m/z | 4,8 dimethylnonanoyl carnitine | Fatty Acyls | 2.08E-02 | 0.89 |
| 11.02_477.2308m/z | Methyl cellulose | Organooxygen compounds | 1.19E-03 | 0.89 |
| 1.45_136.0386n | Hypoxanthine | Imidazopyrimidines | 1.10E-02 | 0.89 |
| 2 | Butane-2,3-diol | Organooxygen compounds | 3.92E-03 | 0.89 |
| 11.53_263.1641m/z | Methyl (3b,11x)-3-Hydroxy-8-oxo-6-eremophilen-12-oate | Prenol lipids | 6.36E-06 | 0.88 |
| 12.33_464.3347m/z | 3-Hydroxy-11Z-octadecenoylcarnitine | Fatty Acyls | 9.74E-05 | 0.88 |
| 5.48_768.4588m/z | PS(P-16:0/17:2(9Z,12Z)) | Glycerophospholipids | 1.12E-05 | 0.87 |
| 1.45_297.0570m/z | N-Acetyldjenkolic acid | Carboxylic acids and derivatives | 4.57E-02 | 0.87 |
| 9.70_311.2229m/z | (±)9-HpODE | Unclassified | 4.12E-02 | 0.87 |
| 1.46_319.0659m/z | Oxoadipic acid | Keto acids and derivatives | 2.09E-02 | 0.85 |
| 1.46_251.0783m/z | Deoxyinosine | Purine nucleosides | 1.98E-02 | 0.85 |
| 5.49_362.2227m/z | Celabenzine | Unclassified | 5.58E-04 | 0.84 |
| 11.53_585.3435m/z | PG(20:0/0:0) | Glycerophospholipids | 8.64E-06 | 0.84 |
| 8.89_368.2199n | PGG2 | Fatty Acyls | 2.86E-06 | 0.84 |
| 7.34_582.3403m/z | PHOOA-PA | Glycerophospholipids | 3.09E-03 | 0.84 |
| 12.61_374.3029m/z | N-Myristoyl Glutamine | Fatty Acyls | 9.27E-06 | 0.83 |
| 4.46_169.0497m/z | Vanillic acid | Benzene and substituted derivatives | 2.06E-02 | 0.83 |
| 5.61_224.1259m/z | Capryloylglycine | Carboxylic acids and derivatives | 3.79E-03 | 0.82 |
| 10.60_311.2229m/z | (5S,7S)-7-Methyl-1,6-dioxaspiro[4.5]decane | Unclassified | 1.06E-02 | 0.81 |
| 0.79_202.1439m/z | (4S)-7-Hydroxy-4-isopropenyl-7-methyl-2-oxo-oxepanone | Unclassified | 1.74E-02 | 0.80 |
| 8.06_268.1919m/z | (E,E)-2,4-Decadienoic isobutylamide | Fatty Acyls | 4.43E-02 | 0.79 |
| 12.59_178.0630n | 1,2-Dihydroxy-3,4-epoxy-1,2,3,4-tetrahydronaphthalene | Tetralins | 9.06E-05 | 0.79 |
| 9.29_288.1935m/z | Mebeverine metabolite (1-Butanol, 4-[ethyl[2-(4-methoxyphenyl)-1-methylethyl]amino]-) | Unclassified | 1.26E-02 | 0.79 |
| 8.29_636.3856m/z | PHOHA-PC | Glycerophospholipids | 1.80E-02 | 0.78 |
| 13.87_565.4229m/z | DG(14:0/15:0/0:0) | Glycerolipids | 1.63E-04 | 0.76 |
| 4.14_169.0496m/z | PHLORACETOPHENONE | Organooxygen compounds | 1.10E-03 | 0.76 |
| 6.97_621.0304n | ADP-ribose 1″-2″ cyclic phosphate | Unclassified | 8.21E-04 | 0.76 |
| 8.05_614.5369m/z | Glycerol triundecanoate | Glycerolipids | 2.90E-02 | 0.75 |
| 7.80_619.3691m/z | 3'-N-Acetyl-4'-O-(10,12-octadecadienoyl)fusarochromanone | Fatty Acyls | 2.68E-03 | 0.75 |
| 0.72_228.0747n | Zebularine | Unclassified | 2.82E-02 | 0.75 |
| 0.75_221.0901n | N-Acetyl-D-glucosamine | Organooxygen compounds | 4.85E-02 | 0.75 |
| 12.48_497.3602m/z | 1α,25-Dihydroxy-2α-(3-hydroxypropyl)vitamin D3 | Unclassified | 2.10E-07 | 0.75 |
| 1.45_252.0859n | Nebularine | Purine nucleosides | 3.42E-02 | 0.75 |
| 11.31_295.2278m/z | 3,4,7,11-Tetramethyl-6E,10Z-tridecadienal | Fatty Acyls | 1.87E-02 | 0.75 |
| 5.07_243.0632m/z | 2,6-Dioxo-6-phenylhexanoate | Unclassified | 2.93E-02 | 0.75 |
| 0.52_247.1430n | (R)-3-hydroxybutyrylcarnitine | Fatty Acyls | 4.41E-02 | 0.74 |
| 6.84_578.0111m/z | 3-(ADP)-2-phosphoglycerate | Unclassified | 2.23E-05 | 0.74 |
| 10.30_433.1280m/z | (9R,10S)-rel-(-)-9,10-bis(Acetyloxy)-9,10-dihydro-5-methoxy-8,8-dimethyl-2-phenyl-4H,8H-benzo[1,2-b:3,4-b']dipyran-4-one | Unclassified | 1.88E-02 | 0.73 |
| 9.18_311.2462n | N-tetradecanoyl-L-Homoserine lactone | Unclassified | 4.60E-02 | 0.73 |
| 14.27_407.1871m/z | Dimethylenetriurea | Unclassified | 4.23E-02 | 0.73 |
| 1.39_302.0661m/z | N-Benzoyl-4-hydroxyanthranilate | Unclassified | 2.16E-02 | 0.73 |
| 197 | Tartaric acid | Organooxygen compounds | 2.29E-04 | 0.72 |
| 38 | Resorcinol |  | 1.57E-03 | 0.72 |
| 8.27_260.0564m/z | Oxolinic acid | Unclassified | 3.09E-02 | 0.71 |
| 7.46_602.0182m/z | UDP-L-Ara4FN | Unclassified | 2.66E-05 | 0.71 |
| 6.41_462.2595m/z | PC(O-6:0/6:0)[U] | Unclassified | 5.73E-04 | 0.71 |
| 14.04_150.0774m/z | Pteridine | Unclassified | 1.36E-04 | 0.71 |
| 6.52_489.9587m/z | Zalcitabine triphosphate | Unclassified | 1.82E-04 | 0.71 |
| 11.53_177.1273m/z | Rhubafuran | Benzene and substituted derivatives | 3.09E-05 | 0.71 |
| 11.53_121.0650m/z | 4-Hydroxystyrene | Benzene and substituted derivatives | 8.94E-04 | 0.70 |
| 6.64_519.3093m/z | PG(20:2(11Z,14Z)/0:0) | Glycerophospholipids | 4.11E-08 | 0.70 |
| 1.39_267.0966n | Deoxyguanosine | Purine nucleosides | 1.22E-02 | 0.70 |
| 1.39_324.0481m/z | 2-succinyl-5-enolpyruvyl-6-hydroxy-3-cyclohexene-1-carboxylate | Unclassified | 2.81E-02 | 0.69 |
| 1.39_402.0642m/z | Tolrestat | Unclassified | 1.63E-02 | 0.69 |
| 13.45_415.3888m/z | Behenoylglycine | Carboxylic acids and derivatives | 4.96E-05 | 0.69 |
| 1.39_267.0926m/z | 6-(Pentylthio)purine | Unclassified | 2.02E-02 | 0.69 |
| 8.44_288.2898m/z | 14-methyl palmitic acid | Unclassified | 2.80E-04 | 0.68 |
| 4.09_197.0424m/z | (E)-2-(hexa-3,5-dien-1-yn-1-yl)-5-(prop-1-yn-1-yl)thiophene | Fatty Acyls | 3.24E-02 | 0.68 |
| 0.52_399.0083m/z | Erythrulose 1-phosphate | Unclassified | 2.98E-02 | 0.68 |
| 5.93_226.1444m/z | isovaleryl-L-carnitine | Fatty Acyls | 4.88E-02 | 0.68 |
| 8.32_294.2430m/z | α-parinaric acid | Unclassified | 4.45E-02 | 0.68 |
| 1.39_334.0769m/z | N-Succinyl-2-amino-6-ketopimelate | Carboxylic acids and derivatives | 1.69E-02 | 0.68 |
| 6.56_499.2760n | Aconine | Unclassified | 9.41E-06 | 0.67 |
| 48 | Ethanolamine | Organonitrogen compounds | 4.61E-02 | 0.66 |
| 11.56_583.1185m/z | N-Acetylsulfadiazine | Unclassified | 4.97E-02 | 0.66 |
| 5.39_724.4322m/z | PE(18:3(9Z,12Z,15Z)/14:0) | Glycerophospholipids | 4.23E-04 | 0.66 |
| 11.76_410.2668n | PGF2α-11-acetate methyl ester | Unclassified | 1.50E-03 | 0.66 |
| 343 | 1-hexacosanol |  | 6.04E-03 | 0.66 |
| 0.75_211.0847n | 3-Methoxytyrosine | Carboxylic acids and derivatives | 1.96E-02 | 0.65 |
| 0.81_128.0345m/z | 4-Oxoglutaramate | Unclassified | 1.03E-02 | 0.64 |
| 5.39_353.1998n | PIPENZOLATE | Unclassified | 3.14E-04 | 0.63 |
| 10.32_231.1751m/z | 3-Methyl-alpha-ionyl acetate | Prenol lipids | 1.76E-02 | 0.62 |
| 12.74_379.1574m/z | L-365260 | Unclassified | 4.65E-02 | 0.61 |
| 14.13_453.3336m/z | PA(O-20:0/0:0) | Glycerophospholipids | 5.58E-04 | 0.61 |
| 5.87_497.2766m/z | Fumitremorgin B | Indoles and derivatives | 1.31E-02 | 0.60 |
| 12.41_409.2565m/z | 3-methoxy Prostaglandin F1α | Unclassified | 3.58E-02 | 0.60 |
| 10.78_625.3346m/z | PHOHA-PG | Glycerophospholipids | 8.04E-04 | 0.60 |
| 11.07_298.3469m/z | 10S,14S-Dimethyl-1-octadecene | Fatty Acyls | 8.55E-05 | 0.59 |
| 5.35_196.1335m/z | Benzenemethanol, 2-(2-aminopropoxy)-3-methyl- | Unclassified | 1.94E-02 | 0.59 |
| 10.80_204.1384m/z | (all-E)-3,5,7-Tridecatriene-9,11-diyn-1-ol | Fatty Acyls | 5.42E-06 | -0.59 |
| 0.68_106.0867m/z | Diethanolamine | Organonitrogen compounds | 1.54E-02 | -0.59 |
| 11.36_388.3937m/z | Cholest-5-ene | Steroids and steroid derivatives | 1.72E-05 | -0.59 |
| 11.19_280.2635m/z | 4E,14Z-Sphingadiene | Sphingolipids | 1.21E-03 | -0.59 |
| 11.27_317.0795m/z | 2-S-cysteinyl-DOPA | Carboxylic acids and derivatives | 1.40E-04 | -0.59 |
| 5.13_185.1418n | N-methyl-Gabapentin | Unclassified | 3.96E-02 | -0.59 |
| 10.38_200.2010m/z | Cyclododecanone | Organooxygen compounds | 4.84E-03 | -0.59 |
| 3.15_156.0422m/z | Pentanoic acid, 4-amino-, (R)-; Valeric acid, 4-amino-, (R)-(+)-; (R)-(+)-4-Aminovaleric acid; (R)-4-Aminopentanoic acid | Unclassified | 2.42E-02 | -0.59 |
| 0.62_106.0292m/z | Picolinic acid | Pyridines and derivatives | 3.28E-02 | -0.59 |
| 6.78_311.1466m/z | Phenylalanyl-Glutamate | Carboxylic acids and derivatives | 6.41E-03 | -0.60 |
| 0.52_234.0970m/z | N-(1-Deoxy-1-fructosyl)alanine | Organooxygen compounds | 1.85E-05 | -0.60 |
| 0.51_187.1260m/z | 5-Butyl-4-ethylthiazole | Azoles | 1.78E-04 | -0.60 |
| 8.90_172.1697m/z | Eucalyptol | Oxanes | 3.00E-02 | -0.60 |
| 0.52_151.1228m/z | 2-AI | Unclassified | 2.81E-04 | -0.61 |
| 7.05_301.1987m/z | Lysyl-Histidine | Carboxylic acids and derivatives | 2.79E-03 | -0.61 |
| 0.52_130.1589m/z | Dibutylamine | Carboxylic acids and derivatives | 3.13E-06 | -0.61 |
| 11.06_207.1380m/z | Eremopetasinorone A | Organooxygen compounds | 1.47E-03 | -0.61 |
| 11.66_463.3426m/z | 3α,7α,12α,24-tetrahydroxy-24-methyl-5β-cholestan-26-oic acid | Unclassified | 1.37E-04 | -0.61 |
| 8.54_361.2998n | R-1 Methanandamide | Unclassified | 1.43E-03 | -0.62 |
| 10.49_659.0148m/z | PQQ | Quinolines and derivatives | 1.99E-02 | -0.62 |
| 10.57_276.1595m/z | HOMATROPINE | Unclassified | 4.05E-02 | -0.62 |
| 9.78_198.1853m/z | cis-Quinceoxepane | Oxepanes | 3.13E-02 | -0.62 |
| 7.08_299.1104m/z | Lysyl-Asparagine | Carboxylic acids and derivatives | 1.09E-02 | -0.62 |
| 4.83_283.1186m/z | 4-Oxocyclohexanecarboxylate | Unclassified | 2.39E-02 | -0.63 |
| 11.43_336.3261m/z | N-isobutyl-2E,4Z-octadecadienoyl amine | Fatty Acyls | 1.45E-03 | -0.63 |
| 12.25_499.0203m/z | 3,4-Dihydroxyphenylglycol O-sulfate | Organic sulfuric acids and derivatives | 3.11E-02 | -0.63 |
| 11.13_259.1907m/z | 2-Hydroxypropyl 2-isopropyl-5-methylcyclohexyl carbonate | Prenol lipids | 1.63E-02 | -0.63 |
| 11.93_196.1697m/z | 4-Ethyl-2-hexyl-5-methyloxazole | Azoles | 2.32E-03 | -0.63 |
| 10.06_460.2694m/z | 3-O-Acetylepisamarcandin | Coumarins and derivatives | 1.09E-06 | -0.63 |
| 7.95_158.1541m/z | 2-Nonenal | Organooxygen compounds | 4.29E-02 | -0.63 |
| 5.01_303.1780m/z | Arginylglutamine | Carboxylic acids and derivatives | 3.13E-02 | -0.63 |
| 8.54_317.2931n | Phytosphingosine | Organonitrogen compounds | 1.74E-03 | -0.63 |
| 11.56_648.4316m/z | Kurilensoside F | Sterol Lipids | 3.52E-05 | -0.63 |
| 4.91_209.0923m/z | xi-2,3-Dihydro-2-oxo-1H-indole-3-acetic acid | Indoles and derivatives | 6.01E-04 | -0.63 |
| 11.27_261.0169m/z | Sphagnum acid | Unclassified | 7.84E-04 | -0.64 |
| 0.88_226.9655m/z | 5-Sulfo-1,3-benzenedicarboxylic acid | Benzene and substituted derivatives | 3.79E-07 | -0.64 |
| 10.81_196.1696m/z | 7-Isopropyl-5-methylbicyclo[2.2.2]oct-5-en-2-one | Organooxygen compounds | 1.75E-03 | -0.64 |
| 5.78_199.1574n | 11-nitro-1-undecene | Allyl-type 1,3-dipolar organic compounds | 3.25E-02 | -0.64 |
| 0.52_136.0215m/z | Benzothiazole | Benzothiazoles | 6.68E-05 | -0.64 |
| 9.80_531.2742m/z | PG(20:5(5Z,8Z,11Z,14Z,17Z)/0:0) | Glycerophospholipids | 1.02E-02 | -0.64 |
| 11.68_175.1481m/z | 1-Methyl-4-(1-methyl-2-propenyl)-benzene | Prenol lipids | 2.15E-04 | -0.64 |
| 10.22_563.0332m/z | UDP-alpha-D-galactose | Pyrimidine nucleotides | 3.27E-02 | -0.64 |
| 5.78_130.1229m/z | trans-2-trans-4-Heptadien-1-ol | Fatty Acyls | 4.05E-02 | -0.65 |
| 11.72_261.2213m/z | Elaidolinoleic acid | Unclassified | 4.33E-03 | -0.65 |
| 3.79_283.0102m/z | Thien-2-ylacetate | Heteroaromatic compounds | 2.00E-02 | -0.65 |
| 8.56_290.2690m/z | 9-methoxy-pentadecanoic acid | Fatty Acyls | 5.01E-05 | -0.65 |
| 3.85_133.0860m/z | Leucinic acid | Fatty Acyls | 1.83E-04 | -0.65 |
| 13.20_222.0615m/z | N-(2-Hydroxyethyl)iminodiacetic acid | Unclassified | 3.88E-02 | -0.65 |
| 3.85_208.1312n | 5S,6S-epoxy-15R-hydroxy-ETE | Organooxygen compounds | 9.08E-04 | -0.65 |
| 10.88_214.2166m/z | 2-Methylcyclododecanone | Organooxygen compounds | 7.17E-05 | -0.65 |
| 8.42_273.2668n | C16 Sphinganine | Sphingolipids | 6.89E-03 | -0.66 |
| 0.62_143.0108m/z | (S)-3-Hydroxyisobutyrate | Hydroxy acids and derivatives | 1.30E-02 | -0.66 |
| 0.51_199.1801m/z | 2-Butyl-4,5-diethyloxazole | Azoles | 2.48E-05 | -0.66 |
| 0.62_175.0027m/z | 4-aminobenzoate | Benzene and substituted derivatives | 1.49E-02 | -0.66 |
| 11.27_276.0528m/z | Mebendazole metabolite (2-Amino-5-benzoylbenzimidazole) | Unclassified | 3.24E-03 | -0.66 |
| 11.10_312.2322m/z | Biperiden | Organonitrogen compounds | 1.14E-06 | -0.66 |
| 5.29_267.0998m/z | 1,2-Bis(4-hydroxyphenyl)-2-propanol | Unclassified | 1.21E-02 | -0.66 |
| 4.90_131.1068m/z | α,α-dimethyl valeric acid | Unclassified | 2.72E-02 | -0.67 |
| 9.87_288.2534m/z | 6-Hexadecenoic acid, 16-hydroxy-; Delta6-Isoambrettolic acid | Unclassified | 4.30E-02 | -0.67 |
| 11.10_102.0918m/z | 3-Methylbutyraldehyde oxime | Unclassified | 2.05E-06 | -0.67 |
| 13.34_116.1073m/z | Bis (2-hydroxypropyl) amine | Unclassified | 2.40E-05 | -0.68 |
| 0.54_122.0964m/z | Styrene | Benzene and substituted derivatives | 2.42E-04 | -0.68 |
| 8.75_244.2637m/z | 7-Ethyltridecan-6-one | Unclassified | 6.67E-05 | -0.68 |
| 11.07_428.3372m/z | 15(S)-15-methyl PGF2α isopropyl ester | Unclassified | 4.07E-02 | -0.68 |
| 10.89_280.2637m/z | Sclareol oxide | Prenol lipids | 1.86E-02 | -0.69 |
| 11.63_560.3793m/z | C-6 NBD-dihydro-Ceramide | Unclassified | 6.48E-05 | -0.69 |
| 10.74_261.2213m/z | Ximenynic acid | Fatty Acyls | 3.23E-02 | -0.69 |
| 8.49_230.2480m/z | Xestoaminol C | Sphingolipids | 1.63E-05 | -0.69 |
| 8.72_272.2585m/z | C16 Sphingosine | Sphingolipids | 6.36E-05 | -0.69 |
| 5.30_213.1127m/z | 5-Hexyltetrahydro-2-oxo-3-furancarboxylic acid | Lactones | 1.03E-02 | -0.69 |
| 14.77_236.1026m/z | (S)-3-[(Cyanophenylmethyl)amino]-3-oxopropanoic acid | Benzene and substituted derivatives | 3.39E-03 | -0.70 |
| 7.94_313.1987m/z | Glycerol 1-(5-hydroxydodecanoate) | Fatty Acyls | 2.18E-02 | -0.70 |
| 5.07_301.1623m/z | Histidinyl-Gamma-glutamate | Carboxylic acids and derivatives | 2.02E-02 | -0.70 |
| 163 | Glucosaminic acid |  | 1.75E-03 | -0.70 |
| 11.12_196.1697m/z | 5-Ethyl-2-hexyl-4-methyloxazole | Azoles | 2.42E-05 | -0.70 |
| 4.91_258.1710m/z | Hexanoylcarnitine | Fatty Acyls | 6.78E-03 | -0.70 |
| 11.02_277.1799m/z | Buddledin A | Unclassified | 4.18E-05 | -0.71 |
| 8.55_475.1577m/z | 21-dimethylarsinoyl-(7Z, 10Z,13Z,16Z,19Z)-heneicosapentaenoic acid | Fatty Acyls | 4.37E-02 | -0.71 |
| 178 | Citraconic acid | Fatty Acyls | 3.16E-04 | -0.71 |
| 11.35_228.2322m/z | 9S-(2-cyclopentenyl)-1-nonanol | Fatty Acyls | 7.69E-06 | -0.72 |
| 11.37_102.0918m/z | 4-Methylaminobutanal | Unclassified | 3.67E-05 | -0.72 |
| 11.35_130.1592m/z | (E)-3-Octene | Unsaturated hydrocarbons | 3.10E-05 | -0.72 |
| 0.62_103.0507m/z | Cycloserine | Azolines | 4.16E-06 | -0.72 |
| 10.78_381.3477m/z | Terminaline | Unclassified | 3.61E-03 | -0.72 |
| 11.58_232.1696m/z | Farfugin A | Prenol lipids | 5.89E-07 | -0.73 |
| 9.63_318.3004m/z | 4-hydroxysphinganine | Sphingolipids | 7.80E-03 | -0.73 |
| 7.47_266.1519m/z | Chalciporone | Azepines | 4.41E-03 | -0.73 |
| 11.07_459.3558m/z | Perindopril erbumine | Carboxylic acids and derivatives | 8.36E-04 | -0.74 |
| 10.60_147.0805m/z | 4-(1-Methylethenyl)benzaldehyde | Benzene and substituted derivatives | 3.34E-03 | -0.75 |
| 7.06_208.1698m/z | 3,5,8-Megastigmatrien-7-one | Organooxygen compounds | 3.15E-04 | -0.75 |
| 14.77_385.2931m/z | 17,20-dimethyl Prostaglandin F1α | Unclassified | 1.20E-03 | -0.75 |
| 10.49_154.9967m/z | Diazenedicarboxamide | Organonitrogen compounds | 2.81E-04 | -0.75 |
| 0.52_418.3078n | (20S)-1α,20,25-trihydroxy-24-norvitamin D3/(20S)-1α,20,25-trihydroxy-24-norcholecalciferol | Unclassified | 1.31E-05 | -0.75 |
| 244 | N-acetylputrescine | Carboximidic acids and derivatives | 1.41E-05 | -0.75 |
| 1.11_177.1023m/z | N-Hydroxy-1-aminonaphthalene | Naphthalenes | 1.14E-02 | -0.76 |
| 0.91_294.9533m/z | (2R)-O-Phospho-3-sulfolactate | Unclassified | 3.51E-06 | -0.76 |
| 11.27_338.0760n | 6-(2-Amino-2-carboxyethyl)-7,8-dioxo-1,2,3,4,7,8-hexahydroquinoline-2,4-dicarboxylate | Unclassified | 1.36E-03 | -0.77 |
| 10.74_206.1671n | Methyl-delta-ionone | Prenol lipids | 2.25E-02 | -0.77 |
| 1.06_158.1541m/z | (E)-2-nonen-1-al | Unclassified | 1.15E-02 | -0.77 |
| 12.05_659.4759m/z | PA(O-16:0/O-16:0)[U] | Unclassified | 8.12E-04 | -0.78 |
| 10.35_212.2010m/z | 4,7-Megastigmadien-9-ol | Prenol lipids | 2.67E-02 | -0.78 |
| 11.87_205.0171m/z | 4-Methylcatechol 1-sulfate | Organic sulfuric acids and derivatives | 8.10E-06 | -0.78 |
| 0.52_270.2787m/z | 13-heptadecyn-1-ol | Fatty Acyls | 1.77E-05 | -0.79 |
| 11.36_414.3577m/z | Wuhanic acid | Fatty Acyls | 4.69E-05 | -0.79 |
| 0.60_1045.7169m/z | NeuAcalpha2-3Galbeta-Cer(d18:1/20:0) | Unclassified | 2.88E-02 | -0.80 |
| 11.07_338.2454n | 5,6-DHET | Fatty Acyls | 4.69E-03 | -0.80 |
| 9.23_304.2848m/z | 3-Hydroxy-palmitic acid methyl ester | Unclassified | 2.34E-04 | -0.80 |
| 11.66_255.1744m/z | enzacamene | Unclassified | 1.53E-07 | -0.81 |
| 8.38_325.1454m/z | 7-(4-Hydroxyphenyl)-1-phenyl-4-hepten-3-one | Diarylheptanoids | 4.19E-02 | -0.81 |
| 10.37_289.1411m/z | Gemfibrozil, metabolite II | Unclassified | 4.63E-02 | -0.81 |
| 11.89_304.2635m/z | 11-cis-Retinol | Prenol lipids | 6.50E-03 | -0.81 |
| 226 | Aconitic acid | Carboxylic acids and derivatives | 4.35E-04 | -0.82 |
| 10.77_343.2243m/z | 11,12-Epoxyeicosatrienoic acid | Fatty Acyls | 4.93E-04 | -0.82 |
| 6.84_144.1384m/z | 2,4,4-Trimethylcyclopentanone | Organooxygen compounds | 4.04E-02 | -0.82 |
| 0.62_233.9540n | 2-Iodophenol methyl ether | Unclassified | 1.58E-03 | -0.82 |
| 9.54_283.1915m/z | 4-Pentenoic acid, 2-propyl- | Unclassified | 2.52E-02 | -0.82 |
| 10.76_309.1673m/z | S-(3-Methylbutanoyl)-dihydrolipoamide-E | Fatty Acyls | 2.54E-02 | -0.83 |
| 10.74_214.2166m/z | 5-Ethyl-2E-undecen-4-one | Unclassified | 8.92E-04 | -0.83 |
| 7.57_347.0936m/z | D-Fructofuranose 1,2':2,3'-dianhydride | Unclassified | 8.70E-04 | -0.84 |
| 5.01_326.2538m/z | 1,2,3-Tris(1-ethoxyethoxy)propane | Glycerolipids | 1.02E-02 | -0.84 |
| 13.85_270.1628m/z | Cyproheptadine | Dibenzocycloheptenes | 2.87E-02 | -0.84 |
| 0.77_430.8284m/z | 3,5-Diiodo-4-hydroxyphenylpyruvate | Benzene and substituted derivatives | 2.03E-03 | -0.84 |
| 10.60_172.1333m/z | 2,6-nonadienoic acid | Fatty Acyls | 5.39E-05 | -0.85 |
| 11.27_230.0846m/z | N-(3-oxo-hexanoyl)-homoserine thiolactone | Fatty Acyls | 9.70E-05 | -0.85 |
| 10.81_351.1779m/z | 7-hydroxygranisetron | Benzopyrazoles | 1.85E-02 | -0.85 |
| 1.35_174.1490m/z | (+)-Muscarine | Organooxygen compounds | 7.09E-03 | -0.85 |
| 4.51_221.1054m/z | N-(Phenylmethyl)-N-methyl-2-pyridinamine | Unclassified | 2.83E-04 | -0.86 |
| 4.70_211.1442m/z | p-Butylaminobenzoic acid | Unclassified | 2.21E-02 | -0.86 |
| 11.02_225.0910m/z | Dibenzoylmethane | Unclassified | 1.30E-04 | -0.86 |
| 1.36_156.1384m/z | 9-amino-nonanoic acid | Fatty Acyls | 4.74E-03 | -0.86 |
| 4.01_225.1098m/z | Sebacic acid | Fatty Acyls | 6.84E-04 | -0.86 |
| 10.11_334.2353m/z | N-tetradecanoyl-homoserine lactone | Fatty Acyls | 1.19E-02 | -0.87 |
| 0.51_172.1115m/z | Biphenyl | Benzene and substituted derivatives | 2.34E-05 | -0.87 |
| 0.52_346.3081n | 1-O-(2R-methoxy-hexadecyl)-sn-glycerol | Glycerolipids | 7.49E-07 | -0.88 |
| 10.84_313.1623m/z | N6,N6-Dimethyladenosine | Unclassified | 2.07E-04 | -0.89 |
| 128 | N-acetylgalactosamine | Organooxygen compounds | 1.53E-05 | -0.89 |
| 11.27_411.0937m/z | cis-3-(6-Hydroxy-7-methoxy-5-benzofuranyl)acrylic acid glucuronide | Unclassified | 1.94E-04 | -0.90 |
| 199 | Digalacturonic acid | Organooxygen compounds | 1.52E-02 | -0.90 |
| 4.19_223.0941m/z | 5-Pentyltetrahydro-2-oxo-3-furancarboxylic acid | Lactones | 1.27E-02 | -0.90 |
| 2.11_187.1442m/z | Homoarecoline | Unclassified | 2.19E-03 | -0.90 |
| 1.06_163.1230m/z | L-Nicotine | Pyridines and derivatives | 2.73E-02 | -0.91 |
| 11.02_262.1438m/z | 5-Hydroxybisphenol A | Unclassified | 2.42E-04 | -0.91 |
| 11.48_349.2350m/z | AVOCADYNE ACETATE | Unclassified | 1.82E-02 | -0.92 |
| 4.07_205.0835m/z | Peperinic acid | Benzofurans | 7.07E-03 | -0.93 |
| 0.72_402.1972m/z | 2,3-Butanediol apiosylglucoside | Organooxygen compounds | 6.11E-03 | -0.93 |
| 7.10_359.0672m/z | Geranyl diphosphate | Prenol lipids | 3.87E-02 | -0.94 |
| 11.06_328.2613n | Avocadene 1-acetate | Fatty Acyls | 1.31E-02 | -0.94 |
| 136 | Ribonic acid | Organooxygen compounds | 6.01E-05 | -0.96 |
| 6.05_195.1019m/z | ORTHOTHYMOTINIC ACID | Unclassified | 2.83E-02 | -0.96 |
| 5.44_283.1154m/z | Glycerol tripropanoate | Glycerolipids | 9.92E-03 | -0.96 |
| 0.88_121.0399m/z | methyl 4-Pyrimidine Carboxylate | Unclassified | 4.27E-02 | -0.96 |
| 11.63_375.2507m/z | Montanol | Unclassified | 1.65E-03 | -0.97 |
| 176 | N-methylalanine | Carboxylic acids and derivatives | 6.96E-05 | -0.98 |
| 11.63_419.2769m/z | PGF2α isopropyl ester | Unclassified | 1.12E-02 | -0.99 |
| 11.58_590.4259m/z | IC202A | Fatty Acyls | 3.45E-02 | -0.99 |
| 10.81_226.2166m/z | 8E,10E-Tetradecadienal | Fatty Acyls | 7.54E-03 | -1.00 |
| 8.80_283.1914m/z | cis-γ-octenoic acid | Unclassified | 5.28E-03 | -1.00 |
| 0.51_301.1404m/z | Emmotin A | Prenol lipids | 3.89E-08 | -1.01 |
| 1.57_190.1440m/z | 5-Pentyl-1,4-dioxan-2-one | Dioxanes | 3.98E-02 | -1.01 |
| 11.21_214.2166m/z | 7-Ethyl-4E-undecen-6-one | Unclassified | 1.23E-04 | -1.02 |
| 5.18_256.0600m/z | Flindersiamine | Unclassified | 2.36E-02 | -1.02 |
| 3.49_187.1442m/z | Piperidione | Unclassified | 1.32E-02 | -1.03 |
| 11.50_205.0172m/z | 3-Methylcatechol 1-sulfate | Organic sulfuric acids and derivatives | 6.00E-04 | -1.06 |
| 11.44_240.2322m/z | Humulol | Prenol lipids | 1.48E-02 | -1.08 |
| 1.06_197.0647m/z | Galactonic acid | Hydroxy acids and derivatives | 4.47E-02 | -1.08 |
| 0.77_130.9910m/z | Potassium sorbate | Fatty Acyls | 1.28E-03 | -1.08 |
| 11.63_463.3032m/z | 3-dehydroecdysone | Sterol Lipids | 2.20E-02 | -1.08 |
| 0.72_208.0422n | (+)-1-Methylpropyl 3-(methylthio)-2-propenyl disulfide | Organic disulfides | 1.66E-02 | -1.08 |
| 1.04_130.0170m/z | Monomethyl sulfate | Unclassified | 2.82E-04 | -1.09 |
| 11.32_240.2321m/z | germacradienol | Prenol lipids | 1.38E-02 | -1.09 |
| 11.63_236.2140n | 4E,6E,10Z-Hexadecatrien-1-ol | Fatty Acyls | 2.33E-02 | -1.10 |
| 239 | N-acetylornithine | Carboxylic acids and derivatives | 1.20E-05 | -1.10 |
| 11.24_351.2506m/z | MG(16:1(9Z)/0:0/0:0) | Glycerolipids | 1.61E-03 | -1.11 |
| 11.63_278.2479m/z | Crucigasterin 277 | Sphingolipids | 3.89E-02 | -1.12 |
| 0.83_102.1282m/z | Hexylamine | Organonitrogen compounds | 8.76E-03 | -1.12 |
| 11.13_234.1984n | 7Z,11Z,13E-Hexadecatrienal | Fatty Acyls | 2.75E-02 | -1.13 |
| 235 | L-glutamine | Carboxylic acids and derivatives | 1.33E-02 | -1.14 |
| 10.96_239.1650m/z | 7,8-Dehydro-3,4-dihydro-beta-ionol | Prenol lipids | 2.09E-02 | -1.16 |
| 9.11_258.2429m/z | Cryptomeridiol | Unclassified | 4.60E-03 | -1.17 |
| 0.72_550.2343m/z | Galactan | Organooxygen compounds | 2.68E-02 | -1.18 |
| 5.38_309.1674m/z | Thonzylamine | Phenol ethers | 2.74E-03 | -1.19 |
| 0.68_239.0184m/z | RONIDAZOLE | Unclassified | 1.56E-02 | -1.22 |
| 0.60_785.3021m/z | PRIEURANIN ACETATE | Unclassified | 1.88E-04 | -1.22 |
| 0.77_127.0157m/z | Ethylphosphate | Organic phosphoric acids and derivatives | 9.32E-03 | -1.29 |
| 5.91_241.1078m/z | 11-oxo-undeca-5,9-dienoic acid | Fatty Acyls | 4.96E-02 | -1.31 |
| 3.89_181.0835m/z | cis-4-Hydroxycyclohexylacetic acid | Organooxygen compounds | 5.42E-03 | -1.32 |
| 6.90_186.2218m/z | (+/-)-N,N-Dimethyl menthyl succinamide | Unsaturated hydrocarbons | 2.55E-02 | -1.33 |
| 12.92_308.2947m/z | ABIENOL | Unclassified | 4.69E-02 | -1.35 |
| 4.35_251.1255m/z | (-)-11-hydroxy-9,10-dihydrojasmonic acid | Fatty Acyls | 7.08E-03 | -1.36 |
| 3.81_217.1077m/z | 4-hydroperoxy 2-Nonenal | Unclassified | 1.82E-02 | -1.39 |
| 5.04_207.0658m/z | 1,2-Dihydronaphthalene-1,2-diol | Naphthalenes | 3.11E-02 | -1.40 |
| 5.54_307.1519m/z | Alpha-dihydroartemisinin | Prenol lipids | 3.48E-03 | -1.45 |
| 11.24_228.2322m/z | 8E,10E-Tetradecadien-1-ol | Fatty Acyls | 4.01E-03 | -1.47 |
| 8.36_267.1956m/z | Juvenile hormone III | Prenol lipids | 2.47E-02 | -1.48 |
| 2.39_196.9098m/z | 3-iodo-2E-acrylic acid | Fatty Acyls | 6.87E-03 | -1.48 |
| 3.66_209.0785m/z | 5-Butyltetrahydro-2-oxo-3-furancarboxylic acid | Lactones | 1.08E-03 | -1.48 |
| 0.86_418.9158m/z | Calcium glycerophosphate | Unclassified | 1.33E-04 | -1.55 |
| 1.06_102.1282m/z | Triethylamine | Organonitrogen compounds | 4.09E-03 | -1.56 |
| 409 | 1-kestose | Organooxygen compounds | 6.70E-03 | -1.57 |
| 305 | Terephthalic acid | Benzene and substituted derivatives | 2.77E-06 | -1.66 |
| 2.97_200.0763m/z | 1,3,7-Trimethyluric Acid - 13C4,15N3 (2,4,5,6-13C4, 1,3,9-15N3) | Unclassified | 2.03E-02 | -1.70 |
| 9.66_390.3578m/z | MG(19:0/0:0/0:0) | Glycerolipids | 1.40E-03 | -1.75 |
| 0.83_142.0351m/z | Dimercaprol | Thiols | 8.02E-05 | -1.83 |
| 5.75_226.0185m/z | CAY10562 | Unclassified | 3.29E-02 | -1.85 |
| 5.40_221.0816m/z | 5,6,7,8-tetrahydro-2-Naphthoic Acid | Unclassified | 4.16E-03 | -1.85 |
| 220 | Methyltetrahydrophenanthrenone |  | 4.38E-06 | -1.86 |
| 9.57_301.2982n | Sphinganine | Organonitrogen compounds | 2.59E-04 | -1.87 |
| 0.72_300.1654m/z | Thiazinamium | Benzothiazines | 4.10E-02 | -1.91 |
| 7.04_190.1263m/z | S-(3-Methyl-2-butenyl) 2-methylpropanethioate | Thiocarboxylic acids and derivatives | 8.20E-03 | -1.95 |
| 9.62_281.1759m/z | Sirenin | Unclassified | 3.66E-02 | -1.98 |
| 4.49_228.1363n | 5-Heptyltetrahydro-2-oxo-3-furancarboxylic acid | Lactones | 4.63E-03 | -2.02 |
| 4.42_218.1541m/z | 4'-Methyl-alpha-pyrrolidinopropiophenone | Unclassified | 3.11E-02 | -2.09 |
| 7.02_306.2429m/z | trans-Dehydroandrosterone | Steroids and steroid derivatives | 4.66E-02 | -2.23 |
| 2.96_198.0794m/z | 5-Methylthioribose | Organooxygen compounds | 2.05E-02 | -2.24 |
| 9.71_281.1759m/z | Limonen-6-ol-pivalate | Unclassified | 2.25E-02 | -2.27 |
| 6.57_188.2011m/z | Undecanal | Organooxygen compounds | 5.00E-02 | -2.33 |
| 10.60_336.2509m/z | N-palmitoyl glycine | Carboxylic acids and derivatives | 1.89E-03 | -2.37 |
| 8.37_283.1914m/z | 2-ene-Valproic acid | Fatty Acyls | 2.45E-02 | -2.42 |
| 4.55_182.0815m/z | 4-Hydroxy-4-(3-pyridyl)-butanoic acid | Pyridines and derivatives | 4.41E-02 | -2.49 |
| 9.75_353.1946m/z | 14,14,14-Trifluoro-11E-tetradecenyl acetate | Fatty Acyls | 3.49E-02 | -2.55 |
| 4.49_274.2014m/z | (6R,8Z)-6-Hydroxy-3-oxotetradecenoic acid | Unclassified | 6.80E-03 | -2.59 |
| 5.29_310.0916n | N,N',N",N'"-Tetraacetylglycoluril | Unclassified | 7.99E-03 | -2.93 |
| 4.26_178.0501m/z | 5-Hydroxyindol-2-carboxylic acid | Unclassified | 2.59E-02 | -3.38 |
| 8.96_247.1329m/z | Abscisic acid | Prenol lipids | 1.67E-02 | -3.51 |
| 1.15_176.0781m/z | (S)(+)-Allantoin | Unclassified | 7.44E-03 | -3.75 |
| 6.04_211.0971m/z | 3,4-Methyleneazelaic acid | Fatty Acyls | 3.67E-02 | -3.77 |
| 1.29_243.0379m/z | 3-(Phosphoacetylamido)-L-alanine | Unclassified | 2.81E-02 | -3.78 |
| 3.43_228.0357m/z | Dimethipin | Unclassified | 8.02E-03 | -4.50 |
| 1.00_177.0395m/z | Ascorbic acid | Dihydrofurans | 2.98E-02 | -5.39 |
| 1.14_314.0832m/z | Norchelerythrine | Unclassified | 9.30E-03 | -5.99 |
| 8.97_245.1181m/z | 2-(2-Methylpropoxy)naphthalene | Naphthalenes | 3.10E-02 | -6.23 |
| 3.44_230.0327m/z | Carbendazim | Benzimidazoles | 1.09E-02 | -6.28 |
| 9.34_463.2221m/z | 1-heptadecanoyl-sn-glycerol 3-phosphate | Unclassified | 2.86E-02 | -6.43 |
| 4.26_380.0955m/z | cyclo-dopa 5-O-glucoside | Unclassified | 3.73E-02 | -6.61 |
| 0.99_337.0778m/z | Acaciabiuronic acid | Organooxygen compounds | 3.10E-02 | -11.06 |
| 0.82_337.0778m/z | 2-O-beta-D-Glucopyranuronosyl-D-mannose | Organooxygen compounds | 4.42E-02 | -11.13 |
| 0.99_473.0527m/z | S-Cysteinosuccinic acid | Carboxylic acids and derivatives | 2.91E-02 | -32.23 |
