# Supplementary material for: Investigating the impact of long-term bristlegrass coverage on rhizosphere microbiota, soil metabolites, and carbon–nitrogen dynamics for pear agronomic traits in orchards
Source: Front Microbiol. 2024 Sep 5;15:1461254. doi: 10.3389/fmicb.2024.1461254 (PMC11411186; doi:10.3389/fmicb.2024.1461254)
Supplement: Supplementary file 6 [file Table_6.docx]

**Table S6.** DMs of between SC mode and CC mode in 20-40cm rhizosphere soil layer in pear orchard by UHPLC-MS and GC-MS.

| **Alignment ID** | **Metabolite name** | **Class** | **P-value** | **log2(FCSC20-40vsCC20-40)** |
| --- | --- | --- | --- | --- |
| 11.82_773.5334m/z | PA(14:1(9Z)/24:1(15Z)) | Glycerophospholipids | 7.21E-03 | 34.80 |
| 3.78_264.9755m/z | Bis(2-furanylmethyl) disulfide | Heteroaromatic compounds | 3.77E-02 | 31.46 |
| 4.78_567.1695m/z | D-Vacciniin | Benzene and substituted derivatives | 1.31E-02 | 31.17 |
| 4.60_394.1977m/z | Diacetylfusarochromanone | Benzopyrans | 4.22E-02 | 31.15 |
| 2.87_279.0590m/z | Trifluridine | Pyrimidine nucleosides | 1.72E-02 | 30.56 |
| 1.19_237.0626m/z | Xanthopterin-B2 | Unclassified | 4.42E-02 | 29.98 |
| 13.68_206.0206m/z | Perflutren | Organofluorides | 4.37E-07 | 29.96 |
| 3.86_344.1074m/z | E3040 | Unclassified | 4.89E-02 | 29.80 |
| 0.82_178.0713m/z | Fructosamine | Organooxygen compounds | 3.87E-02 | 29.39 |
| 10.03_473.1605m/z | N-Glycolyl-D-glucosamine | Unclassified | 2.99E-02 | 29.37 |
| 1.73_249.0626m/z | Ara-HX | Purine nucleosides | 3.85E-03 | 29.06 |
| 1.29_213.0488m/z | Metsulfovax | Unclassified | 1.42E-02 | 28.39 |
| 1.16_269.1609m/z | Histidylleucine | Carboxylic acids and derivatives | 1.44E-02 | 28.09 |
| 5.17_345.1062m/z | S 26191 | Unclassified | 1.36E-02 | 28.06 |
| 1.28_147.0767m/z | Pyrrolidonecarboxylic acid | Carboxylic acids and derivatives | 2.58E-02 | 27.70 |
| 0.77_173.0923m/z | Valylglycine | Carboxylic acids and derivatives | 1.02E-02 | 27.32 |
| 10.59_762.5277m/z | PS(16:0/18:1(9Z))[U] | Unclassified | 5.93E-03 | 10.77 |
| 4.63_358.1264n | Sweroside | Unclassified | 2.25E-02 | 9.84 |
| 11.53_739.5148n | PE(P-16:0/20:4(5Z,8Z,10E,14Z)(12OH[S])) | Glycerophospholipids | 5.79E-03 | 9.32 |
| 5.23_331.1078m/z | Camptothecin | Unclassified | 3.64E-02 | 8.25 |
| 11.24_599.5034m/z | DG(16:0/20:4(8Z,11Z,14Z,17Z)/0:0) | Glycerolipids | 1.31E-04 | 7.94 |
| 1.57_267.0726m/z | Xanthosine | Purine nucleosides | 2.30E-02 | 7.82 |
| 0.79_523.0893m/z | METHYL 7-DESHYDROXYPYROGALLIN-4-CARBOXYLATE | Unclassified | 2.91E-02 | 6.88 |
| 1.10_264.0738m/z | 8-hydroxy-2'-deoxy Guanosine | Unclassified | 9.17E-03 | 6.75 |
| 2.09_277.0862m/z | Glutamylmethionine | Carboxylic acids and derivatives | 4.64E-02 | 6.68 |
| 0.94_265.1382m/z | Valinopine | Unclassified | 2.75E-02 | 6.58 |
| 9.96_762.5276m/z | PG(16:0/18:3(6Z,9Z,12Z)) | Glycerophospholipids | 9.79E-03 | 6.47 |
| 13.53_812.5193m/z | PS(O-16:0/20:2(11Z,14Z)) | Glycerophospholipids | 2.47E-02 | 6.37 |
| 7.33_318.0620m/z | Adefovir | Imidazopyrimidines | 2.66E-02 | 6.27 |
| 3.86_339.0703m/z | Aesculin | Coumarins and derivatives | 4.21E-02 | 6.24 |
| 1.13_347.0107m/z | Phenolic phosphate | Unclassified | 1.83E-02 | 6.15 |
| 11.85_944.4679m/z | Krm 1657 | Unclassified | 1.36E-02 | 6.12 |
| 2.09_299.0684m/z | 2-Acetylaminophenoxazin-3-one | Benzoxazines | 3.59E-02 | 6.09 |
| 1.53_280.0687m/z | 6-Succinoaminopurine | Imidazopyrimidines | 2.99E-02 | 5.97 |
| 1.53_309.0340m/z | Ethidimuron | Unclassified | 1.20E-02 | 5.86 |
| 12.94_810.5994m/z | PC(20:3(6,8,11)-OH(5)/P-18:1(9Z)) | Unclassified | 1.61E-02 | 5.82 |
| 12.92_793.5968n | PC(20:3(5Z,8Z,11Z)/P-18:1(11Z)) | Glycerophospholipids | 3.27E-02 | 5.72 |
| 9.53_484.2682m/z | LysoPE(0:0/15:0) | Glycerophospholipids | 1.48E-02 | 5.63 |
| 4.51_237.0764m/z | Allyl phenoxyacetate | Benzene and substituted derivatives | 4.33E-02 | 5.51 |
| 345 | D-mannose | Organooxygen compounds | 8.58E-03 | 5.50 |
| 0.77_177.0396m/z | 2-Keto-3-deoxy-D-gluconic acid | Keto acids and derivatives | 8.57E-03 | 5.45 |
| 10.02_723.4410m/z | PI(12:0/15:0) | Glycerophospholipids | 1.25E-02 | 5.41 |
| 11.26_788.5433m/z | PS(18:0/18:2(9Z,12Z)) | Glycerophospholipids | 1.93E-02 | 5.38 |
| 6.29_310.1757m/z | Isopropalin | Unclassified | 4.05E-02 | 5.33 |
| 1.08_243.0982m/z | Metharbital | Diazines | 8.38E-03 | 5.32 |
| 6.10_329.0999m/z | Cimifugin | Unclassified | 2.53E-02 | 5.29 |
| 2.06_291.0929m/z | 2-p-Tolyl-5,6,7,8-tetrahydrobenzo[d]imidazo[2,1-b]thiazole | Unclassified | 2.84E-02 | 4.90 |
| 1.05_201.0762m/z | 2,5-Dimethyl-4-ethoxy-3(2H)-furanone | Dihydrofurans | 1.28E-02 | 4.57 |
| 2.81_323.0496m/z | Dazomet | Unclassified | 1.44E-02 | 4.53 |
| 2.11_279.1011m/z | gamma-Glutamylmethionine | Carboxylic acids and derivatives | 4.64E-02 | 4.49 |
| 1.06_244.1061n | Hydroxyprolylhydroxyproline | Carboxylic acids and derivatives | 3.92E-02 | 4.48 |
| 1.65_267.0734m/z | S,S-Dimethyl-beta-propiothetin | Unclassified | 4.65E-02 | 4.47 |
| 2.87_257.0769m/z | Glutamyl-Glutamate | Carboxylic acids and derivatives | 1.27E-02 | 4.46 |
| 7.46_185.0600m/z | Odyssic acid | Fatty Acyls | 4.37E-02 | 4.46 |
| 13.20_781.5606n | PC(16:0/20:4(5Z,8Z,11Z,14Z)) | Glycerophospholipids | 1.68E-02 | 4.45 |
| 5.85_265.0715m/z | TUBAIC ACID | Unclassified | 4.05E-02 | 4.41 |
| 4.92_237.0766m/z | Carpacin | Unclassified | 4.97E-02 | 4.32 |
| 6.09_313.1259m/z | Glutaminyllysine | Carboxylic acids and derivatives | 2.66E-02 | 4.31 |
| 0.73_341.1086m/z | D-Maltose | Organooxygen compounds | 3.58E-02 | 4.28 |
| 0.79_208.1005m/z | Ethyl 4-(acetylthio)butyrate | Fatty Acyls | 4.15E-02 | 4.27 |
| 7.27_205.0864m/z | 2-Phenyl-4-pentenal | Benzene and substituted derivatives | 1.17E-02 | 4.17 |
| 1.16_233.1132m/z | Succinylproline | Unclassified | 4.54E-02 | 4.17 |
| 12.97_785.5919n | PE(17:0/22:2(13Z,16Z)) | Glycerophospholipids | 2.23E-03 | 4.14 |
| 1.36_264.0738m/z | 8-Hydroxy-deoxyguanosine | Purine nucleosides | 2.18E-02 | 4.13 |
| 0.75_591.1773m/z | 4-Carboxynevirapine | Steroids and steroid derivatives | 5.00E-02 | 4.09 |
| 2.02_335.0837m/z | Flumioxazin | Benzoxazines | 3.76E-02 | 4.04 |
| 0.71_711.2197m/z | Glycogen | Organooxygen compounds | 9.24E-04 | 4.00 |
| 8.50_335.0523m/z | 1-[4,9-Dihydro-2-(methylthio)-1,3-thiazino[6,5-b]indol-4-yl]-2-propanone | Indoles and derivatives | 2.19E-02 | 3.99 |
| 4.89_408.2133m/z | PC(5:0/5:0) | Glycerophospholipids | 4.78E-02 | 3.99 |
| 3.76_244.1180m/z | 3-Hydroxy-N6,N6,N6-trimethyl-L-lysine | Carboxylic acids and derivatives | 4.01E-02 | 3.99 |
| 8.44_485.1993m/z | Patrinoside | Unclassified | 2.40E-02 | 3.98 |
| 1.28_283.0676m/z | beta-D-3-Ribofuranosyluric acid | Imidazopyrimidines | 1.49E-02 | 3.98 |
| 1.61_283.0683m/z | 5-Methylbarbiturate | Unclassified | 3.41E-02 | 3.94 |
| 3.86_304.1011n | Vicine | Organooxygen compounds | 4.72E-02 | 3.87 |
| 1.26_281.0527m/z | 9-Riburonosylhypoxanthine | Unclassified | 1.25E-02 | 3.86 |
| 4.51_277.1556m/z | Albine | Unclassified | 1.95E-02 | 3.82 |
| 1.36_231.0982m/z | Prolyl-Alanine | Carboxylic acids and derivatives | 2.60E-02 | 3.82 |
| 0.75_306.0953n | Starch acetate | Carboxylic acids and derivatives | 3.35E-02 | 3.81 |
| 9.33_485.3276m/z | 24a,24b-Dihomo-9,10-secocholesta-5,7,10(19),24a-tetraen-1alpha,3,25-triol | Sterol Lipids | 9.07E-02 | 3.75 |
| 0.72_433.0195m/z | 6-Hydroxyluteolin 6,3'-dimethyl ether 7-sulfate | Polyketides | 8.02E-03 | 3.73 |
| 1.13_188.0557m/z | N-Acetyl-L-glutamic acid | Carboxylic acids and derivatives | 3.24E-02 | 3.72 |
| 10.73_367.2457m/z | DG(8:0/0:0/8:0) | Glycerolipids | 3.84E-04 | 3.65 |
| 0.73_323.0790m/z | Quindoxin | Diazanaphthalenes | 2.56E-02 | 3.60 |
| 2.81_255.0620m/z | N-Carbamoyl-2-amino-2-(4-hydroxyphenyl)acetic acid | Carboxylic acids and derivatives | 1.45E-02 | 3.57 |
| 0.73_267.0952n | Neuraminic acid | Organooxygen compounds | 4.85E-02 | 3.57 |
| 10.30_455.2418m/z | PA(0:0/16:0) | Glycerophospholipids | 2.89E-02 | 3.55 |
| 0.72_349.1318m/z | O-Desmethylquinidine | Unclassified | 1.94E-02 | 3.51 |
| 11.02_703.5734m/z | SM(d18:0/16:1(9Z)) | Sphingolipids | 2.72E-02 | 3.48 |
| 4.93_374.2288m/z | N-3-oxo-hexadec-11(Z)-enoyl-L-Homoserine lactone | Unclassified | 3.43E-02 | 3.48 |
| 3.70_298.0969m/z | 5'-Methylthioadenosine | 5'-deoxyribonucleosides | 4.14E-02 | 3.46 |
| 5.76_259.1232m/z | Sudan II | Unclassified | 4.46E-02 | 3.42 |
| 0.72_425.0609m/z | O-Desmethyloxotolrestat sulfate | Unclassified | 2.95E-03 | 3.42 |
| 0.65_316.9478m/z | beta-D-fructose 2,6-bisphosphate | Organooxygen compounds | 1.70E-04 | 3.42 |
| 4.44_302.1496m/z | Febrifugine | Unclassified | 3.48E-02 | 3.40 |
| 6.00_313.1259m/z | Lysylglutamine | Carboxylic acids and derivatives | 2.28E-02 | 3.40 |
| 3.99_255.0508m/z | 2-(Methylthiomethyl)furan | Heteroaromatic compounds | 1.48E-02 | 3.38 |
| 0.79_150.0411m/z | 2-Amino-3-hydroxypropanoic acid | Carboxylic acids and derivatives | 2.11E-03 | 3.37 |
| 11.87_730.5985n | SM(d18:0/18:1(11Z)) | Sphingolipids | 1.61E-02 | 3.36 |
| 0.73_445.0725m/z | Chlorbufam | Unclassified | 6.98E-03 | 3.35 |
| 5.98_153.0057m/z | 1,4-Dithiothreitol | Organooxygen compounds | 2.57E-02 | 3.21 |
| 4.95_223.0972m/z | 4-(4-Methoxyphenyl)-2-butanone | Phenol ethers | 1.62E-02 | 3.17 |
| 12.99_817.5579m/z | PI(O-18:0/17:2(9Z,12Z)) | Glycerophospholipids | 3.45E-02 | 3.16 |
| 2.02_267.0961m/z | 3-Hydroxynorpromazine | Unclassified | 4.46E-02 | 3.16 |
| 0.70_179.0795n | Kanosamine | Unclassified | 2.87E-02 | 3.15 |
| 0.70_452.1377m/z | 6-HydroxyKetanserinol | Unclassified | 5.00E-02 | 3.14 |
| 2.02_246.1212n | Valylglutamic acid | Carboxylic acids and derivatives | 4.12E-02 | 3.13 |
| 2.80_146.0602m/z | 4-formyl Indole | Unclassified | 4.72E-02 | 3.06 |
| 0.75_397.0420m/z | Furo[3,4-b]pyridine-3-carboxylic acid, 5,7-dihydro-2-methyl-4-(3-nitrophenyl)-5-oxo-, 2-hydroxyethyl | Unclassified | 3.99E-02 | 3.05 |
| 1.13_257.0414m/z | N-phosphocreatinate(2-) | Carboxylic acids and derivatives | 1.75E-02 | 3.05 |
| 315 | Udp-glucuronic acid | Lactones | 2.48E-03 | 3.03 |
| 1.03_173.0922m/z | (5S,6S)-6-Amino-5-hydroxycyclohexane-1,3-diene-1-carboxyate | Unclassified | 1.61E-02 | 3.02 |
| 12.71_506.1986m/z | O-1,4-α-L-Dihydrostreptosyl-streptidine 6-phosphate | Unclassified | 3.93E-02 | 3.01 |
| 7.05_515.1745m/z | 5''-Phosphoribostamycin | Unclassified | 1.98E-02 | 2.98 |
| 1.05_242.1145m/z | Nitrilacarb | Unclassified | 9.59E-03 | 2.96 |
| 1.46_273.0533m/z | (S)-2,3-Dihydro-6-hydroxy-5-(hydroxyacetyl)-2-isopropenylbenzofuran | Coumarans | 1.66E-02 | 2.92 |
| 0.73_431.1044m/z | Bisnorbiotin | Carboxylic acids and derivatives | 2.60E-02 | 2.91 |
| 1.06_231.0978m/z | N,N-Dihydroxy-L-tyrosine | Unclassified | 1.60E-02 | 2.91 |
| 256 | L-threonic acid | Organooxygen compounds | 1.93E-02 | 2.90 |
| 1.32_241.0803m/z | CAY10554 | Unclassified | 1.50E-02 | 2.86 |
| 0.72_271.0813m/z | Phlorin | Organooxygen compounds | 2.58E-02 | 2.85 |
| 0.72_290.0847m/z | Miserotoxin | Unclassified | 3.59E-02 | 2.85 |
| 11.53_618.3998m/z | Phorbol 12-tiglate 13-decanoate | Unclassified | 1.64E-04 | 2.83 |
| 7.10_503.3379m/z | Sugetriol | Unclassified | 4.40E-02 | 2.80 |
| 1.14_306.0128m/z | 5-Azacytidine 5'-monophosphate | Unclassified | 9.72E-03 | 2.79 |
| 3.36_277.1193m/z | Melatonin | Indoles and derivatives | 2.64E-02 | 2.77 |
| 1.12_212.0531m/z | L-2-Amino-6-oxoheptanedioate | Unclassified | 2.59E-02 | 2.77 |
| 0.71_559.0255m/z | Endothion | Unclassified | 5.75E-03 | 2.74 |
| 0.72_366.1132n | 2-hydroxyestrone | Steroids and steroid derivatives | 3.50E-03 | 2.71 |
| 0.71_181.0709m/z | Galactitol | Organooxygen compounds | 4.58E-02 | 2.70 |
| 2.05_246.1217n | gamma-Glutamylvaline | Carboxylic acids and derivatives | 4.81E-02 | 2.70 |
| 3.16_281.1496m/z | Tyrosyl-Valine | Carboxylic acids and derivatives | 3.76E-02 | 2.69 |
| 1.35_266.0885m/z | 5'-Dehydroadenosine | Unclassified | 3.46E-02 | 2.68 |
| 1.18_231.1346m/z | Spermic acid 2 | Carboxylic acids and derivatives | 1.84E-02 | 2.67 |
| 4.19_568.2658m/z | PS(20:4(5Z,8Z,11Z,14Z)/0:0) | Glycerophospholipids | 3.67E-02 | 2.66 |
| 1.93_283.1290m/z | 2-(2-Phenylacetoxy)propionylglycine | Carboxylic acids and derivatives | 6.67E-04 | 2.63 |
| 10.87_539.3356m/z | LysoPA(22:0/0:0) | Glycerophospholipids | 2.78E-02 | 2.60 |
| 3.86_260.1372n | Leucyl-Glutamate | Carboxylic acids and derivatives | 4.57E-02 | 2.59 |
| 1.37_245.1141m/z | 2,4-Bis(acetamido)-2,4,6-trideoxy-beta-L-altropyranose | Unclassified | 6.62E-03 | 2.59 |
| 0.75_231.0978m/z | Droxidopa | Carboxylic acids and derivatives | 1.15E-02 | 2.59 |
| 1.52_201.1238m/z | Leucylalanine | Carboxylic acids and derivatives | 2.37E-02 | 2.57 |
| 5.17_479.0761m/z | ALIZARIN | Unclassified | 2.19E-02 | 2.56 |
| 1.18_260.0234m/z | 2-Methoxyacetaminophen sulfate | Organic sulfuric acids and derivatives | 3.86E-02 | 2.56 |
| 0.72_342.1163n | Sucrose | Organooxygen compounds | 1.28E-02 | 2.52 |
| 9.66_263.1653m/z | Nootkatone | Unclassified | 4.70E-03 | 2.51 |
| 9.27_499.2152m/z | Iridodial glucoside tetraacetate | Prenol lipids | 3.06E-02 | 2.50 |
| 1.17_280.0687m/z | 8-Hydroxyguanosine | Purine nucleosides | 3.67E-02 | 2.49 |
| 4.12_243.1713m/z | Cycluron | Unclassified | 3.31E-02 | 2.49 |
| 4.04_294.1217n | Tyrosylhydroxyproline | Carboxylic acids and derivatives | 4.60E-02 | 2.48 |
| 4.91_221.0816m/z | Prenylbenzoquinone | Unclassified | 2.04E-02 | 2.48 |
| 3.85_349.0993m/z | DIAZINON | Organic thiophosphoric acids and derivatives | 4.92E-02 | 2.48 |
| 4.86_365.1239m/z | PR-toxin | Unclassified | 3.55E-04 | 2.46 |
| 1.05_235.0487n | 3,4-Dihydro-7-methoxy-2-methylene-3-oxo-2H-1,4-benzoxazine-5-carboxylic acid | Unclassified | 2.66E-02 | 2.46 |
| 1.27_182.9787m/z | Succimer | Fatty Acyls | 1.42E-02 | 2.42 |
| 7.38_301.2020m/z | 4-keto pentadecanoic acid | Fatty Acyls | 1.56E-02 | 2.40 |
| 4.93_174.0552m/z | Phenylacetylglycine | Carboxylic acids and derivatives | 1.08E-02 | 2.40 |
| 0.81_174.0876m/z | 2-Oxoarginine | Keto acids and derivatives | 8.32E-03 | 2.38 |
| 1.33_179.0705m/z | Coniferaldehyde | Phenols | 8.19E-05 | 2.38 |
| 1.05_251.0772m/z | Diethyl tartrate | Hydroxy acids and derivatives | 2.16E-02 | 2.37 |
| 4.12_306.1449m/z | 1H-1,2,4-Triazole-1-propanoic acid, 3-ethyl-4,5-dihydro-5-oxo-4-(2-phenoxyethyl)- | Unclassified | 2.33E-02 | 2.36 |
| 1.65_254.1610m/z | Arginylproline | Carboxylic acids and derivatives | 2.38E-02 | 2.36 |
| 1.27_263.0439m/z | Niflumic Acid | Benzene and substituted derivatives | 2.19E-02 | 2.31 |
| 5.17_411.0886m/z | Zileuton O-glucuronide | Organooxygen compounds | 1.92E-02 | 2.31 |
| 7.98_533.2239m/z | Acarbose (M7) | Unclassified | 1.73E-02 | 2.30 |
| 5.01_235.0609m/z | Austdiol | Azaphilones | 4.71E-02 | 2.28 |
| 10.10_496.3397m/z | PC(0:0/16:0)[U] | Unclassified | 1.19E-02 | 2.28 |
| 10.24_496.3398m/z | PC(16:0/0:0)[S] | Unclassified | 2.71E-02 | 2.26 |
| 10.88_472.3553n | Corosolic acid | Unclassified | 2.72E-02 | 2.26 |
| 0.72_182.0792n | D-Mannitol | Organooxygen compounds | 3.83E-02 | 2.23 |
| 0.71_549.1672m/z | D-(+)-Raffinose | Organooxygen compounds | 1.89E-03 | 2.23 |
| 1.42_547.9674m/z | Gemcitabine (dFdCTP) | Unclassified | 8.53E-03 | 2.19 |
| 7.02_281.0997m/z | 1,1'-(Tetrahydro-6a-hydroxy-2,3a,5-trimethylfuro[2,3-d]-1,3-dioxole-2,5-diyl)bis-ethanone | Organooxygen compounds | 1.63E-02 | 2.18 |
| 4.11_276.1346m/z | 1,2,3,4-Tetrahydro-2-[(isopropylamino)methyl]-7-nitro-6-quinolinecarboxylic acid | Unclassified | 4.76E-02 | 2.17 |
| 5.27_212.0196m/z | o-Nitrobenzoate | Unclassified | 1.39E-02 | 2.17 |
| 11.01_607.3232m/z | OOV-PG | Glycerophospholipids | 4.39E-02 | 2.16 |
| 8.32_413.1784m/z | (-)-11-hydroxy-9,10-dihydrojasmonic acid 11-beta-D-glucoside | Fatty Acyls | 3.81E-02 | 2.14 |
| 2.41_219.1341m/z | Serinyl-Leucine | Carboxylic acids and derivatives | 2.34E-03 | 2.13 |
| 1.14_520.0039m/z | 2',3'-Dideoxyadenosine-5-triphosphate | Purine nucleotides | 2.65E-02 | 2.13 |
| 1.58_221.0922m/z | Indolepyruvate | Indoles and derivatives | 2.43E-02 | 2.13 |
| 0.70_207.0979m/z | AminoDHQ | Unclassified | 4.03E-02 | 2.12 |
| 5.54_561.2165m/z | 1-Methyladenosine | Purine nucleosides | 4.98E-02 | 2.10 |
| 5.07_119.0706m/z | Methyl(S)-3-hydroxybutyrate | Unclassified | 2.95E-02 | 2.08 |
| 0.72_390.1967m/z | Clavamycin F | Unclassified | 1.84E-02 | 2.07 |
| 0.70_689.2111m/z | Stachyose | Organooxygen compounds | 6.77E-06 | 2.07 |
| 0.72_180.0633n | D-Glucose | Organooxygen compounds | 6.55E-03 | 2.07 |
| 0.90_150.0583m/z | THTC | Unclassified | 1.94E-02 | 2.07 |
| 7.05_447.1874m/z | D-Linalool 3-(6''-malonylglucoside) | Fatty Acyls | 1.59E-02 | 2.06 |
| 0.71_439.0761m/z | 5'-Butyrylphosphouridine | Unclassified | 2.07E-02 | 2.05 |
| 10.87_611.3185m/z | OS-PG | Glycerophospholipids | 2.65E-05 | 2.03 |
| 5.17_275.1137m/z | Ethyl (S)-3-hydroxybutyrate glucoside | Fatty Acyls | 2.30E-02 | 2.02 |
| 4.24_327.1086m/z | Paeonoside | Flavonoids | 1.53E-02 | 2.02 |
| 5.18_191.0915m/z | Dambonitol | Organooxygen compounds | 1.64E-02 | 1.99 |
| 353 | Palmitoleic acid | Fatty Acyls | 8.44E-04 | 1.95 |
| 0.68_334.0666n | 2-(alpha-D-Galactosyl)-sn-glycerol 3-phosphate | Unclassified | 3.65E-02 | 1.94 |
| 1.45_297.0570m/z | N-Acetyldjenkolic acid | Carboxylic acids and derivatives | 2.64E-02 | 1.94 |
| 0.73_180.0625n | D-Fructose | Organooxygen compounds | 4.15E-03 | 1.93 |
| 11.01_539.3356m/z | Leptomycin B | Unclassified | 4.09E-02 | 1.92 |
| 249 | Pyroglutamic acid | Carboxylic acids and derivatives | 4.10E-04 | 1.91 |
| 146 | Ciliatine | Organic phosphonic acids and derivatives | 1.87E-05 | 1.90 |
| 1.12_213.1234m/z | Hydroxyprolyl-Valine | Carboxylic acids and derivatives | 2.41E-02 | 1.90 |
| 4.27_243.1712m/z | N-(6-aminohexanoyl)-6-aminohexanoic acid | Fatty Acyls | 3.82E-02 | 1.90 |
| 0.71_217.0477m/z | Sodium (±)-2-(4-methoxyphenoxy)propionate | Unclassified | 4.39E-02 | 1.88 |
| 3.79_295.1274m/z | Methdilazine | Benzothiazines | 3.92E-02 | 1.88 |
| 1.29_292.1039m/z | Linamarin | Organooxygen compounds | 4.55E-02 | 1.86 |
| 0.71_379.0823m/z | 19-Hydroxy-8-O-methyltetrangulol | Unclassified | 1.00E-02 | 1.86 |
| 12.84_298.3469m/z | 9Z-Eicosene | Fatty Acyls | 1.51E-04 | 1.85 |
| 0.72_335.0949m/z | Vicianose | Unclassified | 2.25E-02 | 1.85 |
| 2.69_187.1080m/z | Leucyl-Glycine | Carboxylic acids and derivatives | 7.44E-03 | 1.84 |
| 1.06_365.1057m/z | Trehalulose | Organooxygen compounds | 4.10E-02 | 1.84 |
| 1.37_233.1133m/z | Aspartyl-Valine | Carboxylic acids and derivatives | 1.65E-02 | 1.83 |
| 0.71_519.0168m/z | S-Methyl-5-thio-D-ribose 1-phosphate | Organooxygen compounds | 2.89E-03 | 1.83 |
| 0.75_190.1075m/z | 3-Isopropenylpentanedioic acid | Fatty Acyls | 3.96E-02 | 1.82 |
| 0.72_666.2219n | Cellotetraose | Unclassified | 1.19E-02 | 1.82 |
| 0.73_365.0393m/z | SC-58125 | Unclassified | 3.77E-03 | 1.82 |
| 0.75_221.0901n | N-Acetyl-D-glucosamine | Organooxygen compounds | 1.57E-02 | 1.81 |
| 10.92_357.1884m/z | trans-p-Menthane-7,8-diol 7-glucoside | Prenol lipids | 3.70E-06 | 1.81 |
| 10.53_363.1604m/z | 1,2-Di(2-pyridyl)ethylene | Unclassified | 1.32E-02 | 1.81 |
| 4.12_245.1861m/z | Leucyl-Leucine | Carboxylic acids and derivatives | 2.08E-02 | 1.81 |
| 1.33_217.1189m/z | Meprobamate | Carboxylic acids and derivatives | 4.66E-03 | 1.81 |
| 2.76_233.1496m/z | Ethylenediamine-N,N'-di-a-butyric acid | Unclassified | 5.41E-03 | 1.80 |
| 0.71_763.1815m/z | Phytosulfokine b | Fatty Acyls | 2.58E-06 | 1.80 |
| 1.17_203.1032m/z | N6-Acetyl-N6-hydroxy-L-lysine | Unclassified | 3.73E-03 | 1.79 |
| 3.34_191.0808n | Dihydro-6-isopropyl-2,4-dimethyl-4H-1,3,5-dithiazine | Azacyclic compounds | 4.50E-02 | 1.78 |
| 0.82_133.0131m/z | Malic acid | Hydroxy acids and derivatives | 2.49E-02 | 1.78 |
| 0.72_147.0653m/z | Rhamnose | Organooxygen compounds | 7.40E-03 | 1.77 |
| 4.60_309.1446m/z | 2-Carboxy-1-[5-(2-carboxy-1-pyrrolidinyl)-2-hydroxy-2,4-pentadienylidene]pyrrolidinium | Carboxylic acids and derivatives | 3.53E-02 | 1.77 |
| 0.70_851.2637m/z | Maltopentaose | Organooxygen compounds | 3.57E-02 | 1.77 |
| 1.12_164.0569m/z | 8-Hydroxy-7-methylguanine | Imidazopyrimidines | 4.32E-02 | 1.77 |
| 0.70_104.0710m/z | TROMETHAMINE | Organonitrogen compounds | 2.25E-02 | 1.75 |
| 0.72_504.1693n | 1-Kestose | Organooxygen compounds | 8.06E-03 | 1.75 |
| 1.42_456.9902m/z | DIMETHOATE | Unclassified | 3.88E-02 | 1.75 |
| 2.97_281.1497m/z | Valyltyrosine | Carboxylic acids and derivatives | 4.25E-02 | 1.75 |
| 5.54_493.2292m/z | Ethyl 3-hydroxyoctanoate O-[glucosyl-(1->6)-glucoside] | Fatty Acyls | 4.95E-02 | 1.74 |
| 12.21_623.3190m/z | PI(20:3(8Z,11Z,14Z)/0:0) | Glycerophospholipids | 6.34E-05 | 1.74 |
| 1.35_231.0196n | Benzeneacetamide-4-O-sulphate | Organic sulfuric acids and derivatives | 4.26E-02 | 1.73 |
| 1.23_486.0467m/z | Tizoxanide glucuronide | Benzazepines | 3.08E-02 | 1.71 |
| 1.05_151.0251m/z | Xanthine | Imidazopyrimidines | 2.19E-02 | 1.71 |
| 11.45_623.3189m/z | PKOHA-PG | Glycerophospholipids | 4.42E-04 | 1.71 |
| 3.59_240.0743n | Dinoterb | Unclassified | 9.27E-03 | 1.71 |
| 2.29_310.0020m/z | Flurochloridone | Unclassified | 2.38E-02 | 1.70 |
| 5.07_204.1000n | Diethyl (2R,3R)-2-methyl-3-hydroxysuccinate | Unclassified | 2.91E-02 | 1.70 |
| 2.52_246.1450m/z | (S)-ATPA | Unclassified | 3.96E-03 | 1.69 |
| 0.71_435.0441m/z | 4-iodo-SAHA | Unclassified | 7.45E-03 | 1.69 |
| 1.20_495.1833m/z | Sudan I | Unclassified | 5.00E-02 | 1.69 |
| 0.71_743.0606m/z | 4-(N-Maleimido)benzyltrimethylammonium iodide | Unclassified | 1.25E-02 | 1.69 |
| 0.75_218.1138m/z | N-alpha-Acetyl-L-citrulline | Carboxylic acids and derivatives | 7.16E-03 | 1.68 |
| 6.40_196.0247m/z | 3-Hydroxy-2-methylpyridine-4,5-dicarboxylate | Pyridines and derivatives | 4.12E-02 | 1.66 |
| 7.98_585.3532m/z | PA(12:0/14:1(9Z)) | Glycerophospholipids | 3.73E-03 | 1.66 |
| 5.07_243.0632m/z | 2,6-Dioxo-6-phenylhexanoate | Unclassified | 3.61E-02 | 1.66 |
| 6.07_361.1505m/z | Glucosyl (E)-2,6-Dimethyl-2,5-heptadienoate | Organooxygen compounds | 4.92E-02 | 1.66 |
| 4.28_325.0518m/z | tiopronin | Unclassified | 3.20E-02 | 1.66 |
| 0.81_233.1134m/z | N2-Succinyl-L-ornithine | Carboxylic acids and derivatives | 4.33E-02 | 1.65 |
| 1.57_239.0670m/z | (±)-2-(1-Methylpropyl)-4,6-dinitrophenol | Phenols | 2.97E-02 | 1.65 |
| 1.28_293.1113n | N-Acetylmuramate | Organooxygen compounds | 4.60E-02 | 1.65 |
| 11.53_343.1574m/z | CP 339818 | Unclassified | 8.37E-06 | 1.64 |
| 12.46_382.1453m/z | Meropenem Metabolite (2H-Pyrrole-2-acetic acid, 5-carboxy-4-[[(3S,5S)-5-[(dimethylamino)carbonyl]-3- | Unclassified | 1.41E-02 | 1.64 |
| 0.72_255.1073m/z | Galactosylglycerol | Glycerolipids | 1.40E-02 | 1.64 |
| 7.05_448.1951n | 22-dimethylarsinoyl-(5Z,8Z, 11Z,14Z,17Z,20Z)-docosahexaenoic acid | Fatty Acyls | 2.65E-02 | 1.63 |
| 10.48_391.2456m/z | Cortol | Steroids and steroid derivatives | 4.09E-02 | 1.63 |
| 2.10_186.1126m/z | 1-Ipomeanol | Heteroaromatic compounds | 2.18E-03 | 1.62 |
| 1.14_260.1606m/z | Isoleucyl-Glutamine | Carboxylic acids and derivatives | 2.38E-02 | 1.61 |
| 0.70_147.0533n | L-Glutamate | Carboxylic acids and derivatives | 4.34E-02 | 1.61 |
| 6.70_213.0148m/z | 1,2-Dinitrobenzene | Unclassified | 2.26E-02 | 1.61 |
| 6.07_429.1378m/z | Macrozamin | Unclassified | 4.03E-02 | 1.60 |
| 0.72_342.1397m/z | Bis-D-fructose 2',1:2,1'-dianhydride | Unclassified | 1.68E-02 | 1.59 |
| 9.93_316.1079m/z | Flusilazole | Benzene and substituted derivatives | 4.51E-02 | 1.59 |
| 4.38_304.1658m/z | Pilosine | Unclassified | 4.80E-02 | 1.58 |
| 0.77_255.1375m/z | 8,9,10,11-Tetrafluoro-8E,10E-dodecadien-1-ol | Fatty Acyls | 3.96E-02 | 1.57 |
| 1.27_320.1816m/z | Istamycin AO | Unclassified | 7.06E-03 | 1.56 |
| 1.23_174.1239m/z | Indospicine | Unclassified | 2.63E-02 | 1.55 |
| 1.46_319.0659m/z | Oxoadipic acid | Keto acids and derivatives | 1.50E-02 | 1.55 |
| 1.46_321.9962m/z | 2-Methyl-4-amino-5-hydroxymethylpyrimidine diphosphate | Unclassified | 3.82E-02 | 1.54 |
| 1.24_244.1301m/z | Vinyl-L-NIO | Unclassified | 3.00E-02 | 1.54 |
| 3.38_279.1341m/z | Tyrosyl-Proline | Carboxylic acids and derivatives | 3.75E-02 | 1.54 |
| 2.15_265.1176n | N6-Methyl-2'-deoxyadenosine | Unclassified | 2.91E-02 | 1.54 |
| 1.21_272.9605m/z | Ribose-1-arsenate | Organooxygen compounds | 2.74E-02 | 1.53 |
| 4.03_292.1293m/z | (1xi,3S)-1,2,3,4-Tetrahydro-1-methyl-beta-carboline-1,3-dicarboxylic acid | Harmala alkaloids | 1.63E-02 | 1.53 |
| 1.44_127.0504m/z | Imidazoleacetic acid | Azoles | 2.05E-02 | 1.52 |
| 12.54_623.3552m/z | Amphibine H | Carboxylic acids and derivatives | 6.04E-05 | 1.51 |
| 3.79_227.1397m/z | Isoleucylproline | Carboxylic acids and derivatives | 4.60E-02 | 1.51 |
| 11.29_625.3346m/z | PI(20:2(11Z,14Z)/0:0) | Glycerophospholipids | 1.73E-04 | 1.51 |
| 10.67_657.3249m/z | POB-PI | Glycerophospholipids | 8.39E-06 | 1.51 |
| 0.51_520.5074m/z | Linolenyl palmitate | Fatty Acyls | 2.18E-03 | 1.51 |
| 2.82_247.1290m/z | Aspartyl-Leucine | Carboxylic acids and derivatives | 1.32E-02 | 1.50 |
| 4.25_235.0556m/z | 1-(2-Thienyl)-1-heptanone | Organooxygen compounds | 4.29E-02 | 1.49 |
| 1.42_479.9800m/z | Sodium picosulfate | Unclassified | 4.58E-02 | 1.48 |
| 10.98_417.2096m/z | Spironolactone | Steroids and steroid derivatives | 6.33E-04 | 1.47 |
| 5.18_276.1211n | Triethyl citrate | Carboxylic acids and derivatives | 1.95E-02 | 1.46 |
| 1.45_252.0859n | Nebularine | Purine nucleosides | 3.33E-02 | 1.46 |
| 4.28_245.1860m/z | NH-DVal(NMe)-Val-OMe | Unclassified | 4.78E-02 | 1.46 |
| 1.46_251.0783m/z | Deoxyinosine | Purine nucleosides | 1.31E-02 | 1.46 |
| 1.06_132.0247n | 3-Methyl sulfolene | Dihydrothiophenes | 1.80E-02 | 1.45 |
| 7.84_289.0695m/z | Catechin | Flavonoids | 3.93E-02 | 1.45 |
| 1.27_228.0742n | 2'-Deoxyuridine | Pyrimidine nucleosides | 1.42E-02 | 1.45 |
| 1.30_276.0174m/z | HPAA sulfate | Organic sulfuric acids and derivatives | 3.93E-02 | 1.45 |
| 7.18_235.1337m/z | trans-beta-damascenone | Organooxygen compounds | 1.53E-02 | 1.45 |
| 3.71_229.1554m/z | Valylleucine | Carboxylic acids and derivatives | 2.30E-02 | 1.44 |
| 1.24_418.0594m/z | Sulfoxotolrestat | Unclassified | 4.73E-02 | 1.44 |
| 12.46_352.1781m/z | Pirenzepine | Benzodiazepines | 3.56E-02 | 1.44 |
| 1.45_136.0386n | Hypoxanthine | Imidazopyrimidines | 2.16E-02 | 1.43 |
| 1.39_246.1216n | Glutamylvaline | Carboxylic acids and derivatives | 2.65E-03 | 1.42 |
| 1.14_180.0658m/z | L-Tyrosine | Carboxylic acids and derivatives | 2.18E-02 | 1.41 |
| 2.69_286.1762m/z | Dihydroxymelphalan | Unclassified | 4.58E-02 | 1.40 |
| 10.96_387.1990m/z | Fortimicin B | Unclassified | 5.39E-04 | 1.40 |
| 7.98_585.5201m/z | N-(15-methyl-3-(13-methyl-tetradecanoyloxy)-hexadecanoyl)-glycine | Fatty Acyls | 3.43E-02 | 1.40 |
| 1.05_199.0217m/z | cis-1,2-Dihydroxy-1,2-dihydrodibenzothiophene | Unclassified | 1.20E-02 | 1.39 |
| 5.04_207.0658m/z | 1,2-Dihydronaphthalene-1,2-diol | Naphthalenes | 5.32E-03 | 1.39 |
| 9.27_331.2491m/z | 17-hydroxy-heptadecanoic acid | Fatty Acyls | 1.91E-02 | 1.39 |
| 5.18_322.1863m/z | Arginyl-Phenylalanine | Carboxylic acids and derivatives | 1.61E-02 | 1.39 |
| 72 | Benzylalcohol | Benzene and substituted derivatives | 1.73E-02 | 1.38 |
| 4.26_189.0762m/z | (S)-2-aceto-2-hydroxy-butanoic acid | Unclassified | 2.89E-02 | 1.38 |
| 0.72_144.0425n | Dimethyl fumarate | Fatty Acyls | 2.04E-02 | 1.38 |
| 11.02_477.2308m/z | Methyl cellulose | Organooxygen compounds | 5.75E-05 | 1.38 |
| 1.20_233.1496m/z | Isoleucyl-Threonine | Carboxylic acids and derivatives | 3.39E-02 | 1.37 |
| 6.75_542.3218m/z | SLF | Unclassified | 6.52E-03 | 1.36 |
| 2.34_144.0809m/z | Tryptophol | Indoles and derivatives | 3.17E-02 | 1.35 |
| 6.09_198.0402m/z | 4-Nitroanisole | Unclassified | 4.21E-02 | 1.34 |
| 3.51_229.1554m/z | Isoleucyl-Valine | Carboxylic acids and derivatives | 2.10E-02 | 1.34 |
| 0.77_158.0580n | 2-Isopropylmaleate | Fatty Acyls | 5.16E-03 | 1.34 |
| 12.69_298.3469m/z | 8-Isoprostane | Saturated hydrocarbons | 1.61E-04 | 1.33 |
| 11.53_621.3200m/z | Desmethylergometrine | Unclassified | 4.86E-05 | 1.32 |
| 4.90_206.0817m/z | 3,4-Dihydro-4-[(5-methyl-2-furanyl)methylene]-2H-pyrrole | Heteroaromatic compounds | 3.31E-02 | 1.32 |
| 3.85_293.1144m/z | 6-Hydroxymelatonin | Indoles and derivatives | 4.03E-03 | 1.32 |
| 7.80_619.1685m/z | Pelargonidin 3-(6''-acetylglucoside)-5-glucoside | Unclassified | 2.36E-03 | 1.31 |
| 1.46_135.0298m/z | L-Lactic acid | Hydroxy acids and derivatives | 5.13E-03 | 1.31 |
| 8.29_636.3856m/z | PHOHA-PC | Glycerophospholipids | 2.17E-02 | 1.30 |
| 0.84_169.0861m/z | (+/-)-Methyl 5-acetoxyhexanoate | Fatty Acyls | 1.54E-04 | 1.29 |
| 221 | Cytidine-5-monophosphate |  | 8.38E-05 | 1.28 |
| 0.71_601.1291m/z | 1-Guanidino-1-deoxy-scyllo-inositol 4-phosphate | Unclassified | 1.59E-02 | 1.28 |
| 1.24_283.0916n | Guanosine | Purine nucleosides | 4.84E-02 | 1.27 |
| 10.48_345.1320m/z | N-Acetyl-L-glutamate 5-semialdehyde | Carboxylic acids and derivatives | 5.90E-03 | 1.27 |
| 3.66_217.1077m/z | 4-hydroperoxynonenal | Unclassified | 9.57E-03 | 1.26 |
| 1.42_276.0175m/z | DOPA sulfate | Carboxylic acids and derivatives | 3.13E-02 | 1.26 |
| 4.26_213.0734m/z | Diethyl L-malate | Hydroxy acids and derivatives | 3.84E-02 | 1.25 |
| 8.52_331.1879m/z | Soraphen O | Unclassified | 1.71E-02 | 1.24 |
| 1.03_219.1340m/z | Valyl-Threonine | Carboxylic acids and derivatives | 1.49E-02 | 1.24 |
| 10.78_625.3346m/z | PHOHA-PG | Glycerophospholipids | 8.81E-05 | 1.22 |
| 9.94_375.1780m/z | [4]-Gingerdiol 3,5-diacetate | Phenols | 1.28E-04 | 1.22 |
| 9.64_529.2786m/z | PG(16:0/0:0)[U] | Unclassified | 3.11E-02 | 1.21 |
| 0.71_539.1382m/z | 7,8,4'-Trihydroxy-3',5'-dimethoxyflavanone 4'-O-glucoside | Unclassified | 1.48E-02 | 1.21 |
| 1.60_187.0577m/z | L-Fucose | Organooxygen compounds | 1.15E-02 | 1.20 |
| 1.06_166.0866m/z | 3,4-Dihydro-2H-1-benzopyran-2-one | 3,4-dihydrocoumarins | 4.97E-02 | 1.20 |
| 7.57_646.3790m/z | Hydroxyhomodestruxin B | Peptidomimetics | 5.12E-04 | 1.20 |
| 1.04_153.0408m/z | 6,8-Dihydroxypurine | Imidazopyrimidines | 3.79E-02 | 1.20 |
| 11.53_531.2953m/z | Physapubescin | Steroids and steroid derivatives | 6.78E-05 | 1.19 |
| 3.73_231.1704m/z | 6-nonenoylglycine | Carboxylic acids and derivatives | 2.12E-02 | 1.19 |
| 7.54_631.3689m/z | Goshonoside F4 | Prenol lipids | 1.06E-04 | 1.19 |
| 9.58_531.2931m/z | DAMGO | Unclassified | 2.75E-02 | 1.18 |
| 8.05_614.5369m/z | Glycerol triundecanoate | Glycerolipids | 3.00E-04 | 1.18 |
| 12.46_353.1426m/z | O-Ureidohomoserine | Carboxylic acids and derivatives | 6.40E-03 | 1.18 |
| 10.32_299.2228m/z | 2,5-Dimethyl-3-propylpyrazine | Diazines | 1.61E-02 | 1.17 |
| 1.69_269.1133m/z | N-Feruloylglycine | Unclassified | 1.52E-02 | 1.16 |
| 11.53_632.4157m/z | Synaptolepis factor K1 | Unclassified | 1.18E-03 | 1.16 |
| 0.70_203.0795n | N2-Acetyl-L-aminoadipate | Unclassified | 2.51E-03 | 1.16 |
| 6.97_621.0304n | ADP-ribose 1″-2″ cyclic phosphate | Unclassified | 2.07E-04 | 1.15 |
| 0.71_541.1350m/z | Resveratrol 4'-(6-galloylglucoside) | Stilbenes | 2.79E-02 | 1.14 |
| 11.53_475.2326m/z | rac-5,6-Epoxy-retinoyl-beta-D-glucuronide | Prenol lipids | 7.61E-06 | 1.13 |
| 372 | Elaidic acid | Fatty Acyls | 1.27E-02 | 1.13 |
| 196 | Serine | Carboxylic acids and derivatives | 1.24E-02 | 1.13 |
| 0.98_203.1029m/z | Proclavaminic acid | Unclassified | 4.63E-02 | 1.13 |
| 12.09_298.3466m/z | 10,14-Dimethyl-1-octadecene | Fatty Acyls | 3.38E-04 | 1.13 |
| 10.97_625.3346m/z | Physapruin B | Benzopyrans | 7.81E-04 | 1.11 |
| 0.77_268.0797n | 3-Deoxy-D-glycero-D-galacto-2-nonulosonic acid | Organooxygen compounds | 5.09E-03 | 1.11 |
| 10.26_357.3476m/z | AM3102 | Unclassified | 6.44E-03 | 1.09 |
| 8.38_325.1454m/z | 7-(4-Hydroxyphenyl)-1-phenyl-4-hepten-3-one | Diarylheptanoids | 3.00E-02 | 1.09 |
| 1.39_302.0661m/z | N-Benzoyl-4-hydroxyanthranilate | Unclassified | 4.66E-02 | 1.07 |
| 0.70_176.1031m/z | Citrulline | Carboxylic acids and derivatives | 3.50E-02 | 1.07 |
| 6.02_541.3030m/z | p-hydroxyfosinoprilat | Unclassified | 1.04E-04 | 1.07 |
| 6.33_456.2256m/z | NBD-FTY720 phenoxy | Unclassified | 4.46E-03 | 1.07 |
| 5.40_221.0816m/z | 5,6,7,8-tetrahydro-2-Naphthoic Acid | Unclassified | 1.37E-02 | 1.06 |
| 7.80_619.3691m/z | 3'-N-Acetyl-4'-O-(10,12-octadecadienoyl)fusarochromanone | Fatty Acyls | 8.47E-05 | 1.06 |
| 197 | Tartaric acid | Organooxygen compounds | 3.26E-06 | 1.06 |
| 3.81_217.1077m/z | 4-hydroperoxy 2-Nonenal | Unclassified | 4.37E-02 | 1.05 |
| 7.57_646.0441m/z | UDP-GlcNAc | Pyrimidine nucleotides | 8.60E-04 | 1.05 |
| 11.85_298.3468m/z | 10-Eicosene | Unsaturated hydrocarbons | 1.76E-06 | 1.05 |
| 5.39_353.1998n | PIPENZOLATE | Unclassified | 2.36E-04 | 1.05 |
| 4.19_271.1199m/z | Trigonellinamide | Pyridines and derivatives | 3.85E-02 | 1.04 |
| 11.53_207.1016m/z | Dhelwangin | Organooxygen compounds | 9.67E-06 | 1.04 |
| 3.54_231.1704m/z | Leucyl-Valine | Carboxylic acids and derivatives | 4.93E-02 | 1.03 |
| 6.64_519.3093m/z | PG(20:2(11Z,14Z)/0:0) | Glycerophospholipids | 1.52E-05 | 1.03 |
| 6.17_796.4539m/z | PS(14:0/20:3(8Z,11Z,14Z)) | Glycerophospholipids | 1.74E-04 | 1.03 |
| 1.35_219.1340m/z | Threoninyl-Valine | Carboxylic acids and derivatives | 1.24E-02 | 1.03 |
| 225 | Erythrose | Organooxygen compounds | 3.85E-07 | 1.02 |
| 6.16_407.2436m/z | Annoglabasin C | Prenol lipids | 1.75E-05 | 1.01 |
| 188 | Uracil | Diazines | 1.71E-02 | 1.01 |
| 11.53_586.3505n | PI(O-18:0/0:0) | Glycerophospholipids | 1.30E-05 | 1.01 |
| 1.39_153.0408m/z | Oxypurinol | Imidazopyrimidines | 3.83E-02 | 1.00 |
| 4.21_265.1547m/z | Valyl-Phenylalanine | Carboxylic acids and derivatives | 4.65E-02 | 0.99 |
| 11.07_298.3469m/z | 10S,14S-Dimethyl-1-octadecene | Fatty Acyls | 9.49E-06 | 0.99 |
| 11.04_623.3190m/z | LysoPI(0:0/18:0) | Glycerophospholipids | 5.78E-05 | 0.98 |
| 1.04_186.1006n | Alanyl-Proline | Carboxylic acids and derivatives | 3.32E-02 | 0.98 |
| 4.23_263.1392m/z | Phenylalanylproline | Carboxylic acids and derivatives | 4.92E-02 | 0.96 |
| 7.61_680.4108m/z | OHDdiA-PA | Glycerophospholipids | 1.93E-02 | 0.96 |
| 6.84_578.0111m/z | 3-(ADP)-2-phosphoglycerate | Unclassified | 1.43E-05 | 0.95 |
| 5.98_708.4013m/z | PE(13:0/18:4(6Z,9Z,12Z,15Z)) | Glycerophospholipids | 1.28E-05 | 0.95 |
| 3.29_247.1289m/z | L-beta-aspartyl-L-leucine | Peptidomimetics | 6.96E-03 | 0.94 |
| 1.06_179.0705m/z | COUMARINIC ACID METHYL ETHER | Unclassified | 6.02E-03 | 0.94 |
| 1.20_205.1186m/z | N5-(L-1-Carboxyethyl)-L-ornithine | Unclassified | 2.44E-03 | 0.94 |
| 160 | L-proline | Carboxylic acids and derivatives | 4.31E-02 | 0.94 |
| 1.16_164.0475n | 2-Hydroxycinnamic acid | Cinnamic acids and derivatives | 1.11E-02 | 0.94 |
| 2.68_217.1188m/z | Leucyl-Serine | Carboxylic acids and derivatives | 8.38E-04 | 0.93 |
| 0.68_106.0503m/z | (R)-2-Amino-3-hydroxypropanoic acid | Carboxylic acids and derivatives | 3.19E-02 | 0.93 |
| 11.53_411.0939m/z | Indolylmethylthiohydroximate | Unclassified | 3.99E-02 | 0.92 |
| 0.75_201.0871m/z | 4-Pyridoxic acid | Pyridines and derivatives | 2.42E-02 | 0.92 |
| 8.09_413.9748m/z | Quinoline yellow | Indanes | 4.98E-02 | 0.92 |
| 6.52_489.9587m/z | Zalcitabine triphosphate | Unclassified | 1.29E-04 | 0.92 |
| 5.49_362.2227m/z | Celabenzine | Unclassified | 4.00E-03 | 0.91 |
| 5.52_257.1394m/z | (-)-9,10-dihydrojasmonic acid | Fatty Acyls | 1.03E-02 | 0.91 |
| 5.39_724.4322m/z | PE(18:3(9Z,12Z,15Z)/14:0) | Glycerophospholipids | 1.72E-04 | 0.90 |
| 7.46_602.0182m/z | UDP-L-Ara4FN | Unclassified | 4.87E-04 | 0.90 |
| 8.21_329.2334m/z | 5,8,12-Trihydroxy-9-octadecenoic acid | Fatty Acyls | 3.13E-02 | 0.90 |
| 0.77_216.1233m/z | cis-2,3-Dihydroxy-2,3-dihydro-p-cumate | Unclassified | 5.37E-03 | 0.90 |
| 331 | Pentadecanoic acid | Fatty Acyls | 1.30E-02 | 0.90 |
| 7.41_604.3533m/z | Presqualene diphosphate | Prenol lipids | 1.10E-02 | 0.89 |
| 2.99_231.0841m/z | Ethyl glucoside | Organooxygen compounds | 9.48E-03 | 0.89 |
| 1.27_135.0298m/z | Hydroxypropionic acid | Hydroxy acids and derivatives | 2.96E-03 | 0.89 |
| 1.23_152.0568m/z | 8-Hydroxyadenine | Imidazopyrimidines | 2.78E-02 | 0.89 |
| 0.67_284.8968m/z | Trimetaphosphoric acid | Non-metal oxoanionic compounds | 5.13E-03 | 0.89 |
| 3.35_259.1298m/z | d-Dethiobiotin | Fatty Acyls | 2.30E-03 | 0.89 |
| 5.55_384.2356m/z | 6alpha,9-Difluoro-11beta-hydroxypregn-4-ene-3,20-dione | Unclassified | 3.97E-05 | 0.89 |
| 5.30_354.1756m/z | Dihydrozeatin riboside | Unclassified | 2.06E-04 | 0.88 |
| 0.77_256.0818m/z | beta-D-ribosylnicotinate | Organooxygen compounds | 1.33E-02 | 0.87 |
| 0.79_219.1109n | trans-Zeatin | Imidazopyrimidines | 6.87E-03 | 0.85 |
| 396 | Arachidic acid | Fatty Acyls | 2.62E-05 | 0.85 |
| 2.00_215.1396m/z | Valylvaline | Carboxylic acids and derivatives | 1.82E-02 | 0.84 |
| 14.27_407.1871m/z | Dimethylenetriurea | Unclassified | 1.61E-02 | 0.84 |
| 11.53_584.3349n | PI(P-18:0/0:0) | Glycerophospholipids | 2.48E-05 | 0.84 |
| 411 | Behenic acid | Fatty Acyls | 8.80E-04 | 0.83 |
| 9.26_318.2075m/z | gamma-Sanshool | Fatty Acyls | 5.36E-03 | 0.83 |
| 1.39_152.0568m/z | Guanine | Imidazopyrimidines | 4.62E-02 | 0.82 |
| 7.34_582.3403m/z | PHOOA-PA | Glycerophospholipids | 4.64E-03 | 0.82 |
| 6.41_462.2595m/z | PC(O-6:0/6:0)[U] | Unclassified | 3.92E-04 | 0.81 |
| 1.16_118.0422n | Benzofuran | Benzofurans | 9.03E-03 | 0.80 |
| 1.81_253.1183m/z | 3-Hydroxyhexobarbital | Unclassified | 2.84E-02 | 0.80 |
| 4.68_585.2038m/z | Oxaprozin | Azoles | 9.49E-04 | 0.80 |
| 4.05_175.0603m/z | (-)-Bornesitol | Organooxygen compounds | 1.86E-02 | 0.80 |
| 38 | Resorcinol |  | 1.70E-03 | 0.79 |
| 11.53_263.1641m/z | Methyl (3b,11x)-3-Hydroxy-8-oxo-6-eremophilen-12-oate | Prenol lipids | 2.45E-05 | 0.77 |
| 6.56_499.2760n | Aconine | Unclassified | 2.46E-05 | 0.77 |
| 5.87_497.2766m/z | Fumitremorgin B | Indoles and derivatives | 1.51E-03 | 0.77 |
| 5.48_768.4588m/z | PS(P-16:0/17:2(9Z,12Z)) | Glycerophospholipids | 1.33E-04 | 0.77 |
| 6.44_177.0549m/z | SAFROLGLYCOL | Unclassified | 1.70E-02 | 0.76 |
| 2.89_218.1029m/z | N-Acetylleucine | Carboxylic acids and derivatives | 3.09E-02 | 0.76 |
| 5.20_182.0087m/z | 5-Nitrosalicylate | Unclassified | 1.15E-02 | 0.76 |
| 0.81_219.0977m/z | Serinyl-Hydroxyproline | Carboxylic acids and derivatives | 4.25E-02 | 0.76 |
| 11.53_585.3435m/z | PG(20:0/0:0) | Glycerophospholipids | 2.28E-05 | 0.75 |
| 0.70_134.0449m/z | L-Aspartic acid | Carboxylic acids and derivatives | 1.59E-02 | 0.75 |
| 11.54_583.3278m/z | Trichodermin | Unclassified | 2.16E-04 | 0.74 |
| 8.89_368.2199n | PGG2 | Fatty Acyls | 1.09E-04 | 0.74 |
| 1.27_246.1449m/z | Isoleucyl-Asparagine | Carboxylic acids and derivatives | 4.44E-02 | 0.74 |
| 6.78_563.3358m/z | PA-PA | Glycerophospholipids | 3.08E-05 | 0.73 |
| 4.34_241.1191m/z | 2-Isopropyl-3,5-dimethoxy-6-methylpyrazine | Diazines | 2.96E-02 | 0.73 |
| 2.75_189.1237m/z | N-butanoyl-lhomoserine lactone | Fatty Acyls | 2.21E-02 | 0.72 |
| 0.79_202.1439m/z | (4S)-7-Hydroxy-4-isopropenyl-7-methyl-2-oxo-oxepanone | Unclassified | 2.77E-02 | 0.72 |
| 10.32_231.1751m/z | 3-Methyl-alpha-ionyl acetate | Prenol lipids | 9.14E-03 | 0.72 |
| 10.09_327.2178m/z | 7-hydroxy-10-heptadecen-8-ynoic acid | Fatty Acyls | 2.20E-02 | 0.72 |
| 4.22_282.9629m/z | Dimethylthiophosphate | Organic thiophosphoric acids and derivatives | 2.69E-03 | 0.71 |
| 2.05_217.1548m/z | 5-octenoylglycine | Carboxylic acids and derivatives | 4.59E-02 | 0.71 |
| 1.33_175.1080m/z | Glycyl-Valine | Carboxylic acids and derivatives | 4.79E-02 | 0.70 |
| 9.05_432.3105m/z | 6-keto Testosterone Enanthate | Unclassified | 4.91E-02 | 0.70 |
| 5.45_470.2366n | (R)-1-O-[b-D-Glucopyranosyl-(1->6)-b-D-glucopyranoside]-1,3-octanediol | Fatty Acyls | 5.84E-05 | 0.68 |
| 0.84_129.0181m/z | Itaconic acid | Fatty Acyls | 2.30E-04 | 0.68 |
| 5.33_295.1652m/z | Tyrosyl-Leucine | Carboxylic acids and derivatives | 2.60E-03 | 0.68 |
| 3.37_260.1373n | gamma-Glutamylleucine | Carboxylic acids and derivatives | 1.13E-02 | 0.68 |
| 14.13_453.3336m/z | PA(O-20:0/0:0) | Glycerophospholipids | 8.84E-03 | 0.67 |
| 6.86_403.1889m/z | Hydroquinine, 10,11-dihydroxy- | Unclassified | 2.25E-02 | 0.66 |
| 15.20_547.0205m/z | Isoferulic acid 3-sulfate | Cinnamic acids and derivatives | 1.04E-02 | 0.66 |
| 5.20_382.1842n | 1,2,10-Trihydroxydihydro-trans-linalyl oxide 7-O-beta-D-glucopyranoside | Organooxygen compounds | 1.40E-03 | 0.66 |
| 1.06_126.0664m/z | 2-O-Methylcytosine | Diazines | 3.71E-02 | 0.65 |
| 11.53_121.0650m/z | 4-Hydroxystyrene | Benzene and substituted derivatives | 6.16E-04 | 0.65 |
| 3.74_146.0602m/z | 1H-Indole-3-carboxaldehyde | Indoles and derivatives | 1.63E-02 | 0.64 |
| 1.86_131.0337m/z | Methylsuccinic acid | Fatty Acyls | 9.91E-04 | 0.64 |
| 216 | 3,6-anhydro-d-galactose |  | 3.57E-03 | 0.63 |
| 9.27_327.2178m/z | Sorbitan laurate | Fatty Acyls | 2.62E-02 | 0.63 |
| 0.52_145.0971m/z | 1-Piperideine-2-carboxylic acid | Pyridines and derivatives | 4.04E-03 | 0.62 |
| 1 | 2-picolinic acid | Pyridines and derivatives | 1.58E-03 | 0.61 |
| 367 | Octadecanol | Fatty Acyls | 4.83E-04 | 0.61 |
| 11.27_275.1654m/z | Onchidal | Unclassified | 9.70E-03 | 0.60 |
| 2.75_219.1340m/z | Serinyl-Isoleucine | Carboxylic acids and derivatives | 1.75E-02 | 0.59 |
| 4.77_261.1342m/z | Butyl butyryllactate | Fatty Acyls | 4.82E-02 | 0.59 |
| 7.05_693.4142m/z | Fumonisin C4 | Sphingolipids | 2.98E-04 | 0.59 |
| 10.29_191.1795m/z | 2-Methyl-2-phenyl-undecane | Benzene and substituted derivatives | 9.18E-03 | -0.59 |
| 4.51_156.0422m/z | 5-amino-pentanoic acid | Fatty Acyls | 2.75E-04 | -0.59 |
| 305 | Terephthalic acid | Benzene and substituted derivatives | 3.76E-03 | -0.59 |
| 11.35_130.1592m/z | (E)-3-Octene | Unsaturated hydrocarbons | 4.42E-05 | -0.59 |
| 6.24_441.0826m/z | APIGENIN TRIACETATE | Unclassified | 3.98E-04 | -0.59 |
| 9.40_283.1373m/z | TBS-Corey Lactone Aldehyde | Unclassified | 2.02E-02 | -0.59 |
| 7.16_237.1486m/z | 4-Heptyloxybenzoic acid | Unclassified | 1.12E-02 | -0.60 |
| 0.52_271.2280m/z | 3-Ethyltridecan-2-one | Fatty Acyls | 3.42E-02 | -0.60 |
| 15.40_297.2422m/z | Coriolic acid | Fatty Acyls | 8.00E-03 | -0.62 |
| 0.52_226.1555m/z | Pilocarpine | Unclassified | 2.50E-02 | -0.62 |
| 0.62_168.0291m/z | 2-Amino-3-carboxymuconic acid 6-semialdehyde | Unclassified | 1.27E-03 | -0.62 |
| 6.25_548.1166n | Cyanidin 3-O-(6''-O-succinyl-beta-glucopyranoside) | Unclassified | 8.06E-04 | -0.63 |
| 4.40_156.0422m/z | 2-Amino-2-methylbutanoate | Unclassified | 6.12E-05 | -0.64 |
| 8.40_239.0594m/z | AG-370 | Unclassified | 1.26E-05 | -0.65 |
| 1.65_156.0422m/z | N-Methylethanolaminium phosphate | Organic phosphoric acids and derivatives | 3.46E-03 | -0.65 |
| 1.06_163.1230m/z | L-Nicotine | Pyridines and derivatives | 7.58E-03 | -0.65 |
| 6.78_309.1321m/z | L-Histidine | Carboxylic acids and derivatives | 1.93E-02 | -0.65 |
| 8.75_244.2637m/z | 7-Ethyltridecan-6-one | Unclassified | 2.54E-05 | -0.67 |
| 3.67_160.0792m/z | 2-[(Ethylthio)methyl]furan | Heteroaromatic compounds | 1.47E-02 | -0.68 |
| 12.52_235.1317m/z | xi-4,5-Dihydro-2,4(5)-dimethyl-1H-imidazole | Imidolactams | 2.96E-03 | -0.68 |
| 9.00_254.0935m/z | Carotamine | Benzene and substituted derivatives | 6.45E-03 | -0.68 |
| 17 | Diethylcarbamic acid |  | 6.20E-05 | -0.69 |
| 8.55_475.1577m/z | 21-dimethylarsinoyl-(7Z, 10Z,13Z,16Z,19Z)-heneicosapentaenoic acid | Fatty Acyls | 1.51E-02 | -0.70 |
| 8.93_288.2898m/z | Margaric acid | Fatty Acyls | 2.61E-03 | -0.70 |
| 11.21_214.2166m/z | 7-Ethyl-4E-undecen-6-one | Unclassified | 9.20E-05 | -0.70 |
| 0.91_294.9533m/z | (2R)-O-Phospho-3-sulfolactate | Unclassified | 3.10E-06 | -0.70 |
| 5.43_206.1153m/z | Methyprylon | Piperidines | 2.49E-02 | -0.70 |
| 7.94_313.1987m/z | Glycerol 1-(5-hydroxydodecanoate) | Fatty Acyls | 2.62E-03 | -0.70 |
| 6.25_575.1043m/z | Myricetin 3-(2'',3'',4''-triacetylxyloside) | Polyketides | 8.41E-04 | -0.72 |
| 0.77_178.1075m/z | Anthopleurine | Unclassified | 1.70E-05 | -0.72 |
| 11.56_648.4316m/z | Kurilensoside F | Sterol Lipids | 5.50E-05 | -0.72 |
| 9.00_489.2266m/z | Laserpitin | Unclassified | 1.78E-02 | -0.74 |
| 0.62_175.0027m/z | 4-aminobenzoate | Benzene and substituted derivatives | 1.82E-05 | -0.75 |
| 0.88_226.9655m/z | 5-Sulfo-1,3-benzenedicarboxylic acid | Benzene and substituted derivatives | 4.58E-08 | -0.76 |
| 8.59_403.3064m/z | 2-Hydroxyundecanoate | Fatty Acyls | 1.77E-02 | -0.77 |
| 0.62_103.0507m/z | Cycloserine | Azolines | 5.60E-07 | -0.77 |
| 1.35_174.1490m/z | (+)-Muscarine | Organooxygen compounds | 7.79E-03 | -0.78 |
| 11.07_459.3558m/z | Perindopril erbumine | Carboxylic acids and derivatives | 2.85E-03 | -0.79 |
| 10.90_453.1543m/z | Epimedokoreanin A | Polyketides | 3.06E-02 | -0.79 |
| 0.52_130.1589m/z | Dibutylamine | Carboxylic acids and derivatives | 9.12E-07 | -0.79 |
| 7.47_266.1519m/z | Chalciporone | Azepines | 9.02E-03 | -0.79 |
| 239 | N-acetylornithine | Carboxylic acids and derivatives | 3.57E-08 | -0.79 |
| 3.89_181.0835m/z | cis-4-Hydroxycyclohexylacetic acid | Organooxygen compounds | 1.83E-02 | -0.82 |
| 0.52_139.1231m/z | 2,6-Dimethylaniline | Benzene and substituted derivatives | 7.81E-06 | -0.82 |
| 0.51_283.2646m/z | 1-Heptadecene | Fatty Acyls | 1.33E-02 | -0.83 |
| 0.62_198.0316n | Naphthalic anhydride | Unclassified | 3.02E-04 | -0.83 |
| 0.52_457.4360m/z | Pentacosanoylglycine | Carboxylic acids and derivatives | 8.13E-05 | -0.84 |
| 0.62_143.0108m/z | (S)-3-Hydroxyisobutyrate | Hydroxy acids and derivatives | 1.86E-05 | -0.84 |
| 4.59_345.1310m/z | 1-(beta-D-Glucopyranosyloxy)-3-octanone | Fatty Acyls | 2.51E-02 | -0.84 |
| 5.44_283.1154m/z | Glycerol tripropanoate | Glycerolipids | 2.88E-02 | -0.84 |
| 11.63_396.1461m/z | Ochotensine | Unclassified | 3.29E-02 | -0.85 |
| 0.60_148.9643m/z | Phosphoenolpyruvic acid | Organic phosphoric acids and derivatives | 3.17E-02 | -0.85 |
| 0.60_606.3433m/z | PHOOA-PE | Glycerophospholipids | 3.32E-04 | -0.85 |
| 163 | Glucosaminic acid |  | 5.13E-06 | -0.85 |
| 0.60_169.0089m/z | Desflurane | Organofluorides | 1.90E-07 | -0.87 |
| 4.76_321.2177m/z | 1-O-Desmethyltetrabenazine | Unclassified | 2.18E-02 | -0.87 |
| 4.90_131.1068m/z | α,α-dimethyl valeric acid | Unclassified | 5.09E-04 | -0.88 |
| 178 | Citraconic acid | Fatty Acyls | 3.83E-05 | -0.89 |
| 0.81_143.1180m/z | 6-Acetyl-1,2,3,4-tetrahydropyridine | Pyridines and derivatives | 2.73E-02 | -0.89 |
| 0.77_258.8724m/z | Zinc dithionite | Non-metal oxoanionic compounds | 7.33E-06 | -0.89 |
| 0.75_340.1232m/z | Dinitramine | Unclassified | 4.27E-02 | -0.91 |
| 6.73_516.1561m/z | Furcelleran | Cinnamic acids and derivatives | 3.81E-02 | -0.91 |
| 0.62_106.0292m/z | Picolinic acid | Pyridines and derivatives | 5.36E-06 | -0.92 |
| 0.62_118.0472m/z | ETF | Halohydrins | 4.93E-06 | -0.94 |
| 1.04_130.0170m/z | Monomethyl sulfate | Unclassified | 2.14E-04 | -0.94 |
| 0.60_102.0342m/z | 1,2-Diacylglycerol-LD-PI-pool | Benzene and substituted derivatives | 1.65E-07 | -0.94 |
| 9.00_234.0820m/z | Bis(1-aziridinyl)morpholinophosphine sulfide | Unclassified | 3.73E-02 | -0.94 |
| 7.57_347.0936m/z | D-Fructofuranose 1,2':2,3'-dianhydride | Unclassified | 5.33E-06 | -0.95 |
| 0.51_255.2331m/z | 6-Methylheptan-2-one | Unclassified | 1.47E-02 | -0.98 |
| 6.90_186.2218m/z | (+/-)-N,N-Dimethyl menthyl succinamide | Unsaturated hydrocarbons | 4.71E-03 | -0.99 |
| 0.52_516.4255m/z | TG(10:0/8:0/8:0) | Glycerolipids | 1.15E-02 | -1.00 |
| 0.52_464.3731m/z | 2β-methoxy-1α,25-dihydroxyvitamin D3 | Unclassified | 7.58E-03 | -1.00 |
| 0.60_980.7382m/z | Galabiosylceramide (d18:1/26:1(17Z)) | Sphingolipids | 1.95E-04 | -1.02 |
| 11.87_205.0171m/z | 4-Methylcatechol 1-sulfate | Organic sulfuric acids and derivatives | 5.74E-04 | -1.03 |
| 5.68_199.0969m/z | cis-4-Decenedioic acid | Fatty Acyls | 3.83E-02 | -1.03 |
| 4.57_375.1156m/z | Succinylaminoimidazole carboxamide riboside | Imidazole ribonucleosides and ribonucleotides | 4.03E-02 | -1.04 |
| 0.52_144.1383m/z | (Z)-2-Octenal | Unclassified | 2.72E-08 | -1.05 |
| 0.77_430.8284m/z | 3,5-Diiodo-4-hydroxyphenylpyruvate | Benzene and substituted derivatives | 2.18E-06 | -1.05 |
| 4.51_221.1054m/z | N-(Phenylmethyl)-N-methyl-2-pyridinamine | Unclassified | 5.36E-07 | -1.05 |
| 0.69_728.6443m/z | Tetrac | Unclassified | 1.73E-03 | -1.06 |
| 2.11_187.1442m/z | Homoarecoline | Unclassified | 7.81E-07 | -1.07 |
| 10.02_360.3108m/z | Medica 16 | Unclassified | 4.27E-02 | -1.08 |
| 0.62_536.3591m/z | Perulactone | Steroids and steroid derivatives | 1.60E-03 | -1.09 |
| 0.60_736.3010m/z | Rifamycin B | Polyketides | 5.42E-07 | -1.09 |
| 3.49_187.1442m/z | Piperidione | Unclassified | 4.67E-03 | -1.10 |
| 4.55_182.0815m/z | 4-Hydroxy-4-(3-pyridyl)-butanoic acid | Pyridines and derivatives | 1.28E-02 | -1.11 |
| 1.06_158.1541m/z | (E)-2-nonen-1-al | Unclassified | 1.42E-02 | -1.11 |
| 11.50_205.0172m/z | 3-Methylcatechol 1-sulfate | Organic sulfuric acids and derivatives | 3.44E-05 | -1.11 |
| 1.36_156.1384m/z | 9-amino-nonanoic acid | Fatty Acyls | 8.91E-03 | -1.13 |
| 0.62_203.0340m/z | Demethylmethoxsalen | Unclassified | 4.53E-06 | -1.16 |
| 6.04_211.0971m/z | 3,4-Methyleneazelaic acid | Fatty Acyls | 3.89E-02 | -1.17 |
| 0.68_106.0867m/z | Diethanolamine | Organonitrogen compounds | 5.30E-06 | -1.18 |
| 0.62_146.0011m/z | alpha-Fluoro-beta-alanine | Carboxylic acids and derivatives | 7.01E-03 | -1.21 |
| 0.60_1045.7169m/z | NeuAcalpha2-3Galbeta-Cer(d18:1/20:0) | Unclassified | 4.55E-04 | -1.22 |
| 6.93_547.1095m/z | 6''-O-Malonyldaidzin | Isoflavonoids | 6.55E-03 | -1.24 |
| 0.62_456.3798n | DG(8:0/16:0/0:0) | Glycerolipids | 2.56E-05 | -1.24 |
| 309 | Pinitol | Organooxygen compounds | 1.85E-03 | -1.25 |
| 0.62_120.0446m/z | 3-Pyridylacetic acid | Pyridines and derivatives | 3.79E-09 | -1.30 |
| 0.62_233.9540n | 2-Iodophenol methyl ether | Unclassified | 1.22E-08 | -1.31 |
| 0.52_163.0975m/z | 1-(5-Hydroxy-2-pyrimidinyl)piperazine | Unclassified | 4.32E-02 | -1.31 |
| 0.60_687.3001m/z | Diphenylphosphine isopropyl ester | Unclassified | 1.63E-03 | -1.31 |
| 0.60_679.3110m/z | OS-PI | Glycerophospholipids | 1.22E-04 | -1.32 |
| 6.15_167.1069m/z | Epomediol | Unclassified | 3.67E-02 | -1.33 |
| 10.10_358.2719n | 13,14-Dihydro PGF-1a | Fatty Acyls | 1.44E-01 | -1.35 |
| 0.62_158.0124m/z | Sulfoacetic acid | Unclassified | 1.38E-06 | -1.37 |
| 0.60_744.2898m/z | aclacinomycin S | Unclassified | 3.67E-04 | -1.39 |
| 1.42_199.1806m/z | N-Ethyl trans-2-cis-6-nonadienamide | Fatty Acyls | 1.05E-02 | -1.42 |
| 11.24_228.2322m/z | 8E,10E-Tetradecadien-1-ol | Fatty Acyls | 1.99E-03 | -1.45 |
| 0.60_622.3211m/z | (3a,5b,7a,12a)-24-[(carboxymethyl)amino]-1,12-dihydroxy-24-oxocholan-3-yl-b-D-Glucopyranosiduronic a | Unclassified | 8.75E-04 | -1.45 |
| 6.72_667.1048m/z | Beta-Nicotinamide mononucleotide | Pyridine nucleotides | 6.11E-03 | -1.47 |
| 0.68_239.0184m/z | RONIDAZOLE | Unclassified | 2.60E-07 | -1.47 |
| 2.97_200.0763m/z | 1,3,7-Trimethyluric Acid - 13C4,15N3 (2,4,5,6-13C4, 1,3,9-15N3) | Unclassified | 6.55E-05 | -1.49 |
| 6.72_565.1642m/z | 4-NAPHTHALIMIDOBUTYRIC ACID | Unclassified | 3.62E-03 | -1.49 |
| 0.72_208.0422n | (+)-1-Methylpropyl 3-(methylthio)-2-propenyl disulfide | Organic disulfides | 9.77E-05 | -1.51 |
| 0.52_102.1281m/z | Nervonyl carnitine | Organonitrogen compounds | 6.79E-09 | -1.53 |
| 0.77_130.9910m/z | Potassium sorbate | Fatty Acyls | 2.79E-06 | -1.56 |
| 9.57_301.2982n | Sphinganine | Organonitrogen compounds | 6.08E-04 | -1.61 |
| 9.66_390.3578m/z | MG(19:0/0:0/0:0) | Glycerolipids | 8.73E-03 | -1.67 |
| 0.77_127.0157m/z | Ethylphosphate | Organic phosphoric acids and derivatives | 4.74E-07 | -1.70 |
| 0.69_314.8644m/z | Dimethyl tetrasulfide | Sulfenyl compounds | 4.08E-07 | -1.70 |
| 0.81_246.1451m/z | Prolyl-Hydroxyproline | Unclassified | 1.76E-02 | -1.81 |
| 0.60_931.2375m/z | Malvidin 3-O-(6-O-(4-O-malonyl-alpha-rhamnopyranosyl)-beta-glucopyranoside)-5-O-beta-glucopyranoside | Unclassified | 5.32E-04 | -1.81 |
| 0.83_142.0351m/z | Dimercaprol | Thiols | 1.79E-06 | -1.83 |
| 1.57_190.1440m/z | 5-Pentyl-1,4-dioxan-2-one | Dioxanes | 4.06E-02 | -1.83 |
| 0.95_183.9259m/z | iodoacetamide | Unclassified | 9.74E-05 | -1.90 |
| 220 | Methyltetrahydrophenanthrenone |  | 2.09E-08 | -1.90 |
| 1.04_157.1337m/z | Supinidine | Unclassified | 2.16E-02 | -1.96 |
| 0.86_418.9158m/z | Calcium glycerophosphate | Unclassified | 1.36E-07 | -1.99 |
| 6.71_528.1358m/z | (S)-2,3-Dihydro-3,5-dihydroxy-2-oxo-3-indoleacetic acid 5-[glucosyl-(1->4)-b-D-glucoside] | Organooxygen compounds | 1.84E-02 | -2.03 |
| 4.42_218.1541m/z | 4'-Methyl-alpha-pyrrolidinopropiophenone | Unclassified | 2.29E-05 | -2.08 |
| 8.95_307.1917m/z | CARYOPHYLLENYL ACETATE | Unclassified | 2.29E-02 | -2.09 |
| 5.68_221.0789m/z | Cytosine | Diazines | 2.89E-02 | -2.12 |
| 7.02_306.2429m/z | trans-Dehydroandrosterone | Steroids and steroid derivatives | 4.33E-04 | -2.16 |
| 1.15_176.0781m/z | (S)(+)-Allantoin | Unclassified | 9.26E-06 | -2.20 |
| 1.14_314.0832m/z | Norchelerythrine | Unclassified | 4.50E-04 | -2.45 |
| 2.96_198.0794m/z | 5-Methylthioribose | Organooxygen compounds | 1.85E-04 | -2.51 |
| 1.09_185.1650m/z | 4-Ethyl-5-pentyloxazole | Azoles | 1.03E-02 | -2.61 |
| 1.12_153.1155n | 4-Ethyl-2-methyl-5-propyloxazole | Azoles | 4.48E-02 | -2.64 |
| 7.71_251.1287m/z | Ibuprofen | Phenylpropanoic acids | 1.71E-02 | -2.66 |
| 1.12_153.1387m/z | (R)-Amphetamine | Benzene and substituted derivatives | 4.84E-02 | -2.66 |
| 6.34_179.1070m/z | (+)-Myrtenyl formate | Prenol lipids | 3.10E-02 | -2.82 |
| 1.04_187.1443m/z | 3-[(3-Methylbutyl)nitrosoamino]-2-butanone | Organonitrogen compounds | 1.94E-02 | -2.91 |
| 4.26_178.0501m/z | 5-Hydroxyindol-2-carboxylic acid | Unclassified | 1.51E-02 | -2.93 |
| 4.61_195.1020m/z | 11-oxo-undeca-5,8-dienoic acid | Fatty Acyls | 3.88E-02 | -2.94 |
| 5.29_310.0916n | N,N',N",N'"-Tetraacetylglycoluril | Unclassified | 4.04E-03 | -3.00 |
| 8.96_247.1329m/z | Abscisic acid | Prenol lipids | 4.34E-03 | -3.13 |
| 0.72_176.0547n | Allantoic acid | Carboxylic acids and derivatives | 5.67E-03 | -3.18 |
| 13.20_790.5367m/z | PE(18:0/22:6(4Z,7Z,10Z,12E,16Z,19Z)(14OH)) | Glycerophospholipids | 5.91E-06 | -3.27 |
| 6.53_179.1069m/z | 7E,10-undecadien-4-olide | Fatty Acyls | 4.86E-02 | -3.41 |
| 8.43_274.2615m/z | 9-Decenoylcholine | Organonitrogen compounds | 4.46E-02 | -3.45 |
| 8.65_281.1758m/z | (2E,6E)-1-Hydroxy-2,6,10-farnesatrien-9-one | Prenol lipids | 2.25E-02 | -3.53 |
| 3.43_228.0357m/z | Dimethipin | Unclassified | 3.57E-03 | -3.59 |
| 9.10_437.2910m/z | Chenodeoxycholic acid | Steroids and steroid derivatives | 2.84E-02 | -4.04 |
| 13.32_790.5706m/z | PE(19:1(9Z)/22:4(7Z,10Z,13Z,16Z)) | Glycerophospholipids | 2.27E-06 | -4.10 |
| 6.53_245.0792m/z | Pyrazinamide | Diazines | 4.70E-02 | -4.36 |
| 3.44_230.0327m/z | Carbendazim | Benzimidazoles | 1.33E-03 | -4.59 |
| 7.05_261.1131m/z | NAPROXOL | Unclassified | 2.07E-02 | -5.52 |
| 8.97_245.1181m/z | 2-(2-Methylpropoxy)naphthalene | Naphthalenes | 3.00E-03 | -5.95 |
| 0.54_716.5227m/z | PE(14:0/20:2(11Z,14Z)) | Glycerophospholipids | 4.99E-02 | -24.09 |
| 1.12_121.0888m/z | Phenylethanolaminium | Organonitrogen compounds | 2.68E-02 | -26.95 |
| 9.06_263.1653m/z | Germacra-1(10),4,11(13)-trien-12-al | Unclassified | 3.38E-02 | -27.38 |
| 0.72_134.0561m/z | N-(Carboxyaminomethyl)urea | Unclassified | 4.15E-03 | -29.33 |
| 4.26_356.0989m/z | p-Glucosyloxymandelonitrile | Unclassified | 4.33E-02 | -30.32 |
