# Supplementary material for: Investigating the impact of long-term bristlegrass coverage on rhizosphere microbiota, soil metabolites, and carbon–nitrogen dynamics for pear agronomic traits in orchards
Source: Front Microbiol. 2024 Sep 5;15:1461254. doi: 10.3389/fmicb.2024.1461254 (PMC11411186; doi:10.3389/fmicb.2024.1461254)
Supplement: Supplementary file 7 [file Table_7.docx]

**Table S7.** 50 DMs involved in metabolic pathways from SC vs CC in different rhizosphere soil layers

| **Class** | **Metabolites** | **P-value** | **log_2_(FC_SC0-20cm vs CC0-20cm_)** | **P-value** | **log_2_(FC_SC20-40cmvsCC20-40cm_)** |
| --- | --- | --- | --- | --- | --- |
| **Carbohydrates and carbohydrate conjugates** | Sucrose | 1.33E-02 | 1.48 | 1.28E-02 | 2.52 |
|  | D-(+)-Raffinose | 1.98E-01 | 0.99 | 1.89E-03 | 2.23 |
|  | Stachyose | 2.22E-01 | 0.84 | 6.77E-06 | 2.07 |
|  | D-Mannitol | 1.25E-01 | 3.01 | 3.83E-02 | 2.23 |
|  | N-Acetyl-D-glucosamine | 4.85E-02 | 0.75 | 1.57E-02 | 1.81 |
|  | D-Maltose | 7.35E-02 | 2.29 | 3.58E-02 | 4.28 |
|  | 5-Methylthioribose | 2.05E-02 | -2.24 | 1.85E-04 | -2.51 |
|  | Rhamnose | 3.34E-02 | 3.21 | 7.40E-03 | 1.77 |
|  | beta-D-fructose 2,6-bisphosphate | 3.95E-01 | 0.53 | 1.70E-04 | 3.42 |
| **Lipids and lipid-like molecules** | SM(d18:0/16:1(9Z)) | 1.39E-01 | 7.55 | 2.72E-02 | 3.48 |
|  | PE(14:0/22:2(13Z,16Z)) | 6.45E-05 | 10.15 | 1.49E-01 | 6.59 |
|  | PC(16:0/20:4(5Z,8Z,11Z,14Z)) | 8.04E-02 | 7.75 | 1.68E-02 | 4.45 |
|  | PC(16:0/16:0) | 7.08E-04 | 6.93 | 5.71E-02 | 6.05 |
|  | PGG2 | 2.86E-06 | 0.84 | 1.09E-04 | 0.74 |
|  | Abscisic acid | 1.67E-02 | -3.51 | 4.34E-03 | -3.13 |
|  | 2-Isopropylmaleate | 3.34E-03 | 0.94 | 5.16E-03 | 1.34 |
|  | (S)-Carvone | 9.03E-07 | -0.21 | 2.46E-06 | -0.23 |
|  | PE(18:3(9Z,12Z,15Z)/14:0) | 4.23E-04 | 0.66 | 1.72E-04 | 0.90 |
|  | Galabiosylceramide (d18:1/26:1(17Z)) | 4.56E-01 | -0.25 | 1.95E-04 | -1.02 |
|  | 2-hydroxyestrone | 1.88E-02 | 2.89 | 3.50E-03 | 2.71 |
|  | Presqualene diphosphate | 6.73E-05 | 1.08 | 1.10E-02 | 0.89 |
|  | PA(0:0/16:0) | 1.61E-02 | 1.75 | 2.89E-02 | 3.55 |
| **Nucleosides, nucleotides, and analogues** | Deoxyguanosine | 1.22E-02 | 0.70 | 5.47E-02 | 0.84 |
|  | Deoxyinosine | 1.98E-02 | 0.85 | 1.31E-02 | 1.46 |
|  | 5'-Methylthioadenosine | 1.53E-02 | 2.28 | 4.14E-02 | 3.46 |
|  | Xanthosine | 1.58E-03 | 2.79 | 2.30E-02 | 7.82 |
|  | 8-Hydroxy-deoxyguanosine | 2.88E-03 | 2.03 | 2.18E-02 | 4.13 |
|  | UDP-GlcNAc | 2.12E-04 | 1.05 | 8.60E-04 | 1.05 |
| **Organic acids and derivatives** | N-Acetyl-L-glutamic acid | 2.62E-03 | 2.21 | 3.24E-02 | 3.72 |
|  | 2-Keto-3-deoxy-D-gluconic acid | 6.41E-03 | 4.70 | 8.57E-03 | 5.45 |
|  | Oxoadipic acid | 2.09E-02 | 0.85 | 1.50E-02 | 1.55 |
|  | (S)-3-Hydroxyisobutyrate | 1.30E-02 | -0.66 | 1.86E-05 | -0.84 |
|  | Pyrrolidonecarboxylic acid | 2.59E-02 | 4.59 | 2.58E-02 | 27.70 |
| **Organic nitrogen compounds** | Sphinganine | 2.59E-04 | -1.87 | 6.08E-04 | -1.61 |
|  | Phytosphingosine | 1.74E-03 | -0.63 | 8.61E-02 | -0.41 |
|  | Diethanolamine | 1.54E-02 | -0.59 | 5.30E-06 | -1.18 |
| **Organoheterocyclic compounds** | Guanine | 4.69E-02 | 0.47 | 4.62E-02 | 0.82 |
|  | L-Nicotine | 2.73E-02 | -0.91 | 7.58E-03 | -0.65 |
|  | Hypoxanthine | 1.10E-02 | 0.89 | 2.16E-02 | 1.43 |
|  | Imidazoleacetic acid | 8.28E-03 | 1.99 | 2.05E-02 | 1.52 |
|  | 1-Piperideine-2-carboxylic acid | 1.06E-02 | 2.30 | 4.04E-03 | 0.62 |
|  | Picolinic acid | 3.28E-02 | -0.59 | 5.36E-06 | -0.92 |
|  | 3-Hydroxy-2-methylpyridine-4,5-dicarboxylate | 3.02E-03 | 1.49 | 4.12E-02 | 1.66 |
|  | 4-Hydroxy-4-(3-pyridyl)-butanoic acid | 4.41E-02 | -2.49 | 1.28E-02 | -1.11 |
|  | Cycloserine | 4.16E-06 | -0.72 | 5.60E-07 | -0.77 |
| **Phenylpropanoids and polyketides** | 2-Hydroxycinnamic acid | 7.62E-01 | -0.18 | 1.11E-02 | 0.94 |
|  | 5-Hydroxyferulic acid | 6.62E-04 | -0.17 | 8.28E-05 | -0.22 |
| **Benzenoids** | Phthalic acid | 1.44E-05 | -0.58 | 2.29E-05 | -0.31 |
|  | 4-aminobenzoate | 1.49E-02 | -0.66 | 1.82E-05 | -0.75 |
|  | 4-Hydroxystyrene | 8.94E-04 | 0.70 | 6.16E-04 | 0.65 |
