# Supplementary figures and images for: Investigating the impact of long-term bristlegrass coverage on rhizosphere microbiota, soil metabolites, and carbon–nitrogen dynamics for pear agronomic traits in orchards
Source: Front Microbiol. 2024 Sep 5;15:1461254. doi: 10.3389/fmicb.2024.1461254 (PMC11411186; doi:10.3389/fmicb.2024.1461254)

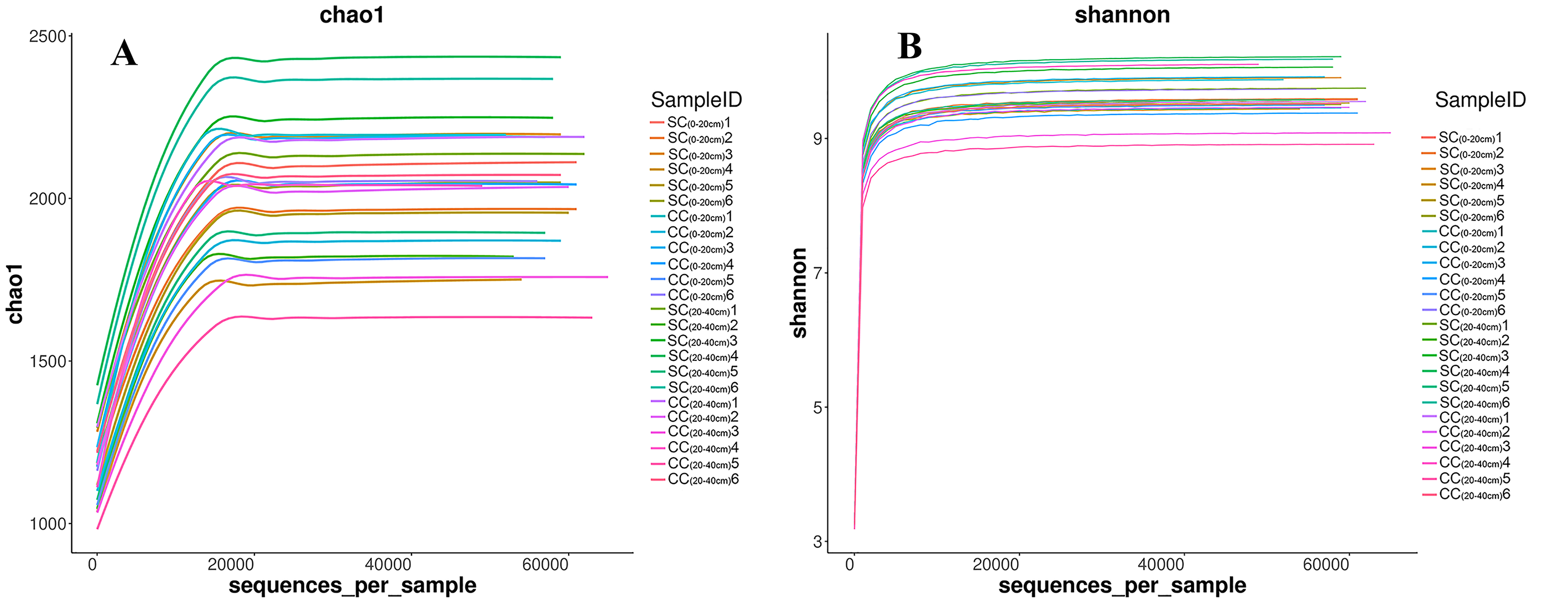

Supplement: Supplementary file 8 [file Image_1.tif]

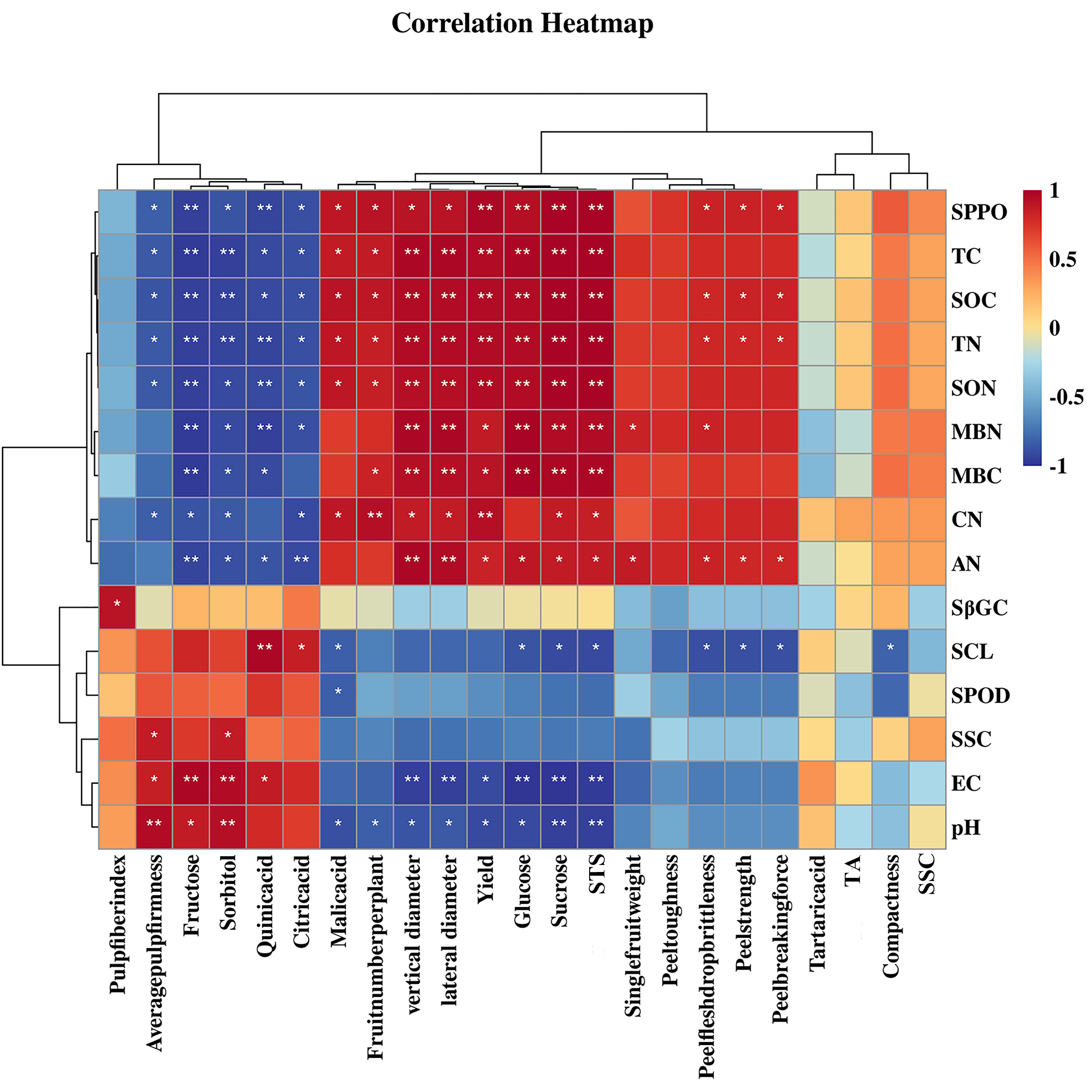

Supplement: Supplementary file 9 [file Image_2.tif]

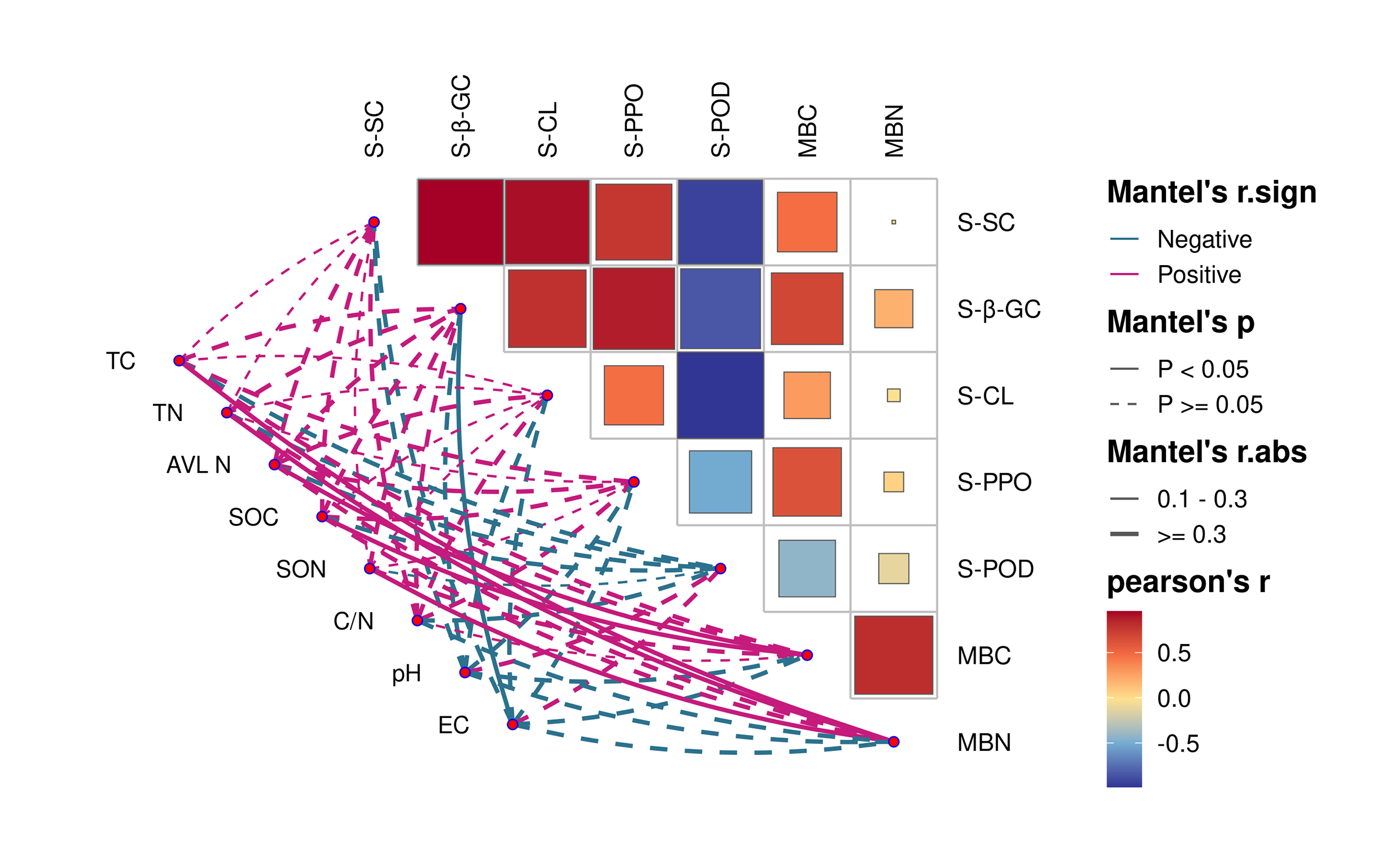

Supplement: Supplementary file 10 [file Image_3.tif]
